# Integrative Plasma Metabolic and Lipidomic Modelling of SARS-CoV-2 Infection in Relation to Clinical Severity and Early Mortality Prediction

**DOI:** 10.3390/ijms241411614

**Published:** 2023-07-18

**Authors:** Samantha Lodge, Nathan G. Lawler, Nicola Gray, Reika Masuda, Philipp Nitschke, Luke Whiley, Sze-How Bong, Bu B. Yeap, Girish Dwivedi, Manfred Spraul, Hartmut Schaefer, Rubén Gil-Redondo, Nieves Embade, Oscar Millet, Elaine Holmes, Julien Wist, Jeremy K. Nicholson

**Affiliations:** 1 Australian National Phenome Center, Health Futures Institute, Murdoch University, Harry Perkins Building, Perth, WA 6150, Australia; sam.lodge@murdoch.edu.au (S.L.); nathan.lawler@murdoch.edu.au (N.G.L.); nicola.gray@murdoch.edu.au (N.G.); reika.masuda@murdoch.edu.au (R.M.); philipp.nitschke@murdoch.edu.au (P.N.); luke.whiley@murdoch.edu.au (L.W.); s.bong@murdoch.edu.au (S.-H.B.); elaine.holmes@murdoch.edu.au (E.H.); 2 Center for Computational and Systems Medicine, Health Futures Institute, Murdoch University, Harry Perkins Building, Perth, WA 6150, Australia; 3 Medical School, University of Western Australia, Perth, WA 6150, Australia; bu.yeap@uwa.edu.au (B.B.Y.); girish.dwivedi@perkins.uwa.edu.au (G.D.); 4 Department of Endocrinology and Diabetes, Fiona Stanley Hospital, Perth, WA 6150, Australia; 5 Department of Cardiology, Fiona Stanley Hospital, Perth, WA 6150, Australia; 6 Bruker Biospin GmbH, 76287 Rheinstetten, Germanyhartmut.schaefer@bruker.com (H.S.); 7 Precision Medicine and Metabolism Laboratory, CIC bioGUNE, Parque Tecnológico de Bizkaia, Bld. 800, 48160 Derio, Spain; rgil@cicbiogune.es (R.G.-R.); nembade@cicbiogune.es (N.E.); omillet@cicbiogune.es (O.M.); 8 Department of Metabolism, Digestion and Reproduction, Faculty of Medicine, Imperial College London, Sir Alexander Fleming Building, South Kensington, London SW7 2AZ, UK; 9 Chemistry Department, Universidad del Valle, Cali 76001, Colombia; 10 Institute of Global Health Innovation, Faculty of Medicine, Imperial College London, Faculty Building, South Kensington Campus, London SW7 2NA, UK

**Keywords:** COVID-19, SARS-CoV-2, NMR spectroscopy, mass spectrometry, plasma IVDr, metabolomics, lipidomics, metabolic phenotyping, diagnostic modelling, lipoproteins, lipids, mortality prediction, patient stratification

## Abstract

An integrative multi-modal metabolic phenotyping model was developed to assess the systemic plasma sequelae of SARS-CoV-2 (rRT-PCR positive) induced COVID-19 disease in patients with different respiratory severity levels. Plasma samples from 306 unvaccinated COVID-19 patients were collected in 2020 and classified into four levels of severity ranging from mild symptoms to severe ventilated cases. These samples were investigated using a combination of quantitative Nuclear Magnetic Resonance (NMR) spectroscopy and Mass Spectrometry (MS) platforms to give broad lipoprotein, lipidomic and amino acid, tryptophan-kynurenine pathway, and biogenic amine pathway coverage. All platforms revealed highly significant differences in metabolite patterns between patients and controls (*n* = 89) that had been collected prior to the COVID-19 pandemic. The total number of significant metabolites increased with severity with 344 out of the 1034 quantitative variables being common to all severity classes. Metabolic signatures showed a continuum of changes across the respiratory severity levels with the most significant and extensive changes being in the most severely affected patients. Even mildly affected respiratory patients showed multiple highly significant abnormal biochemical signatures reflecting serious metabolic deficiencies of the type observed in Post-acute COVID-19 syndrome patients. The most severe respiratory patients had a high mortality (56.1%) and we found that we could predict mortality in this patient sub-group with high accuracy in some cases up to 61 days prior to death, based on a separate metabolic model, which highlighted a different set of metabolites to those defining the basic disease. Specifically, hexosylceramides (HCER 16:0, HCER 20:0, HCER 24:1, HCER 26:0, HCER 26:1) were markedly elevated in the non-surviving patient group (Cliff’s delta 0.91–0.95) and two phosphoethanolamines (PE.O 18:0/18:1, Cliff’s delta = −0.98 and PE.P 16:0/18:1, Cliff’s delta = −0.93) were markedly lower in the non-survivors. These results indicate that patient morbidity to mortality trajectories is determined relatively soon after infection, opening the opportunity to select more intensive therapeutic interventions to these “high risk” patients in the early disease stages.

## 1. Introduction

The coronavirus disease 2019 (COVID-19) pandemic continues to present global challenges to individuals, health systems, and economies and the long-term consequences of the disease are poorly understood. Our ability to develop effective therapeutic management strategies remains reliant on improved understanding of the pathogenic mechanisms associated with severe acute respiratory syndrome coronavirus 2 (SARS-CoV-2) infection [1,2]. Spectroscopic measurements to establish metabolic consequences of human disease have proved to be a powerful tool for exploring existing clinical challenges and can also readily be applied to help understand emergent diseases such as COVID-19 [1].

COVID-19 has spread worldwide infecting over 767 million and killing more than 6.9 million as of July 2023. We have previously shown that, upon SARS-CoV-2 infection, a metabolic “phenoconversion” from healthy through different stages of infection is readily detectable by both Nuclear Magnetic Resonance (NMR) and mass spectrometry [1,3,4,5,6], impacting multiple metabolic pathways and different organ systems. To understand the acute and long-term effects of COVID-19, we and others have compared the plasma of COVID-19 patients with healthy controls using a variety of analytical platforms [7,8,9]. In addition to damaging the respiratory system, SARS-CoV-2 infection affects multiple organs [10,11,12,13], which creates a continuum of emergent metabolic phenotypes some of which appear to relate to respiratory severity. In a meta-analysis of 57 studies, more than 50% of previously hospitalized SARS-CoV-2 survivors were found to have persistent post-acute pathological sequelae including neurologic disorders, general functional impairment, fatigue, and cardiac abnormalities [14]. At the molecular level, infection-related signatures have been found across a range of molecular groups and pathways, some of which persist for several months post-infection. For instance, perturbations in glutamine, glutamate, and taurine are indicative of disruption of hepatic metabolism and, whereas elevated levels of α1-acid Glycoprotein (GlycA) are associated with inflammation, disrupted tryptophan metabolism in some individuals may relate to a neurological impact [2,3,15]. Given the potential socioeconomic impact of SARS-CoV-2 infection, it is important to understand whether respiratory severity of the acute infection is indicative of downstream impact on other organs and systems. If the metabolic dysregulation associated with Post-Acute COVID-19 Syndrome (PACS) [2,16,17] is not closely related to the respiratory infection severity, then follow up and monitoring of patients who experienced mild symptoms may be as important as those that experienced severe respiratory symptoms in their acute infection phase, particularly if the biochemical profile indicates the involvement of multiple systems or organs.

The aim of this study was to comprehensively map the metabolic signature of each severity class to determine if a patient with SARS-CoV-2 infection who experiences mild respiratory symptoms is metabolically distinct from a patient with severe symptoms using a wide range of quantitative parameters (*n* = 1034) derived from targeted profiling using a combination of NMR spectroscopy and ultra-performance high resolution mass spectrometry (UPLC-MS) based on prior knowledge of disrupted molecular pathways [5,6,18,19]. Here, we explore further metabolic data from a previously reported Spanish COVID-19 cohort [5,20] to measure the impact of respiratory disease severity on the systemic metabolic signatures. SARS-CoV-2 participants were stratified into four classes ranging from mild respiratory symptoms to hospitalized with severe respiratory symptoms requiring ventilation in ICU. Serum from a further group of participants collected pre-pandemic were included as non-infected controls. This system for stratifying severity has been widely used in studies on the effects of COVID-19 [21,22]. In the most severely affected category, a 56.1% mortality rate due to an immunological cytokine storm was observed consistent with other similar studies [23,24,25,26]. We derived a predictive model of mortality for the most severe class using multiple combined panels of molecules to achieve a broad phenotype of SARS-CoV-2 infection.

## 2. Results and Discussion

### 2.1. SARS-CoV-2 Infection Induces Reproducible Metabolic and Lipidomic Consequences across Severity Classes Reflective of Systemic Multi-Organ Effects

**Figure 1:**
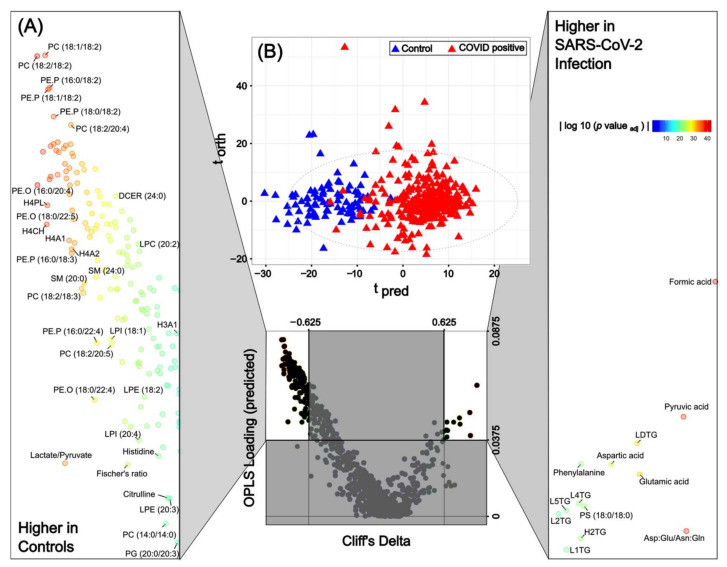
The integrative pan-metabolic model compares the controls and SARS-CoV-2 positive patients. (**A**) Eruption plot of the controls vs. SARS-CoV-2 positive patients for the combined model. (**B**) O-PLS-DA of the control samples (blue triangles) and SARS-CoV-2 positive patients (red triangles) for all four integrated assays, R_2_X = 0.13, AUROC = 0.99. *p*-values for all metabolites/lipids/lipoproteins can be found in Table S5.

As reported in numerous articles, the impact of SARS-CoV-2 infection on the serum metabolite profile causes a disruption of multiple metabolites reflecting dysregulation of pulmonary, cardiovascular, hepatic, and neurological processes [27]. Many of these pathways are immunologically driven via complex cytokine fluctuations [4,6]. Using the integrated metabolite panel from the combined NMR and UPLC-MS assays to compare infected versus non-infected individuals, regardless of respiratory severity class, it was apparent that the differential molecular signature of SARS-CoV-2 infection included reduced levels of phosphocholine, phosphoethanolamine, lysophosphocholine, hexosylceramide, glutamine, Fischer’s ratio (sum of the branched chain amino acids/sum of the aromatic amino acids), histidine, high density lipoprotein parameters, and lactate:pyruvate ratio with higher levels of ABA1, LDL triglycerides, formate, pyruvate, phenylalanine, glutamate, aspartate, neopterin:tryptophan ratio, and the (aspartic acid + glutamic acid)/(asparagine + glutamine) ratio (Figure 1). This profile is consistent with previous studies [28,29,30], including our analysis previously carried out for a subset of this study containing 75 SARS-CoV-2 positive patients [20], and reflects the multi-organ impact of SARS-CoV-2 infection with differentially altered parameters indicating increased cardiovascular risk [31], e.g., apolipoprotein B100/A1 ratio, liver damage, e.g., the Fischer’s ratio and taurine [32] and cellular immune activation e.g., neopterin and kynurenine:tryptophan ratio [3,33].

In the combined O-PLS-DA model calculated using 1034 fully quantified metabolic variables from all four assays: lipids; lipoproteins, and low molecular weight metabolites (MS derived amino acids and tryptophan pathway metabolites and the NMR derived small molecules), an AUROC of 0.99 differentiating infected from non-infected participants was achieved (Table 1). Of the 1034 variables in this model, 598 were significant after correcting for multiple testing. All the significant metabolites in the combined model and the associated Cliff’s delta and adjusted *p*-values can be found in Table S5. The level of statistical significance attached to many of the COVID-19 biomarkers is striking. For instance, pyruvic acid (4.04 fold higher than control; *p*-value 2.67 × 10^−38^), formate (3.71 fold higher than control; adjusted *p*-value of 1.18 × 10^−43^), PC 18:2/18:2 (0.18 fold higher than control; *p*-value of 4.48 × 10^−39^), PE.O 16:0/20:4 (0.18 fold higher than control; *p*-value 4.48 × 10^−39^), Asp+Glu/Asn+Gln ratio (2.71 fold higher than control; *p*-value 1.21 × 10^−38^) and the PE.P 18:1/20:4 (0.17 fold higher than control; *p*-value 2.67 × 10^−38^) were the strongest directly associated markers of SARS-CoV-2 infection (Figure S1).

As shown in Table S1, the average age of the controls is significantly different for each of the COVID-19 severity groups. The 1034 fully quantified metabolic variables from the four assays were therefore corrected for age and the controls versus SARS-CoV-2 positive patients were modelled using O-PLS-DA (Figure S2). It can clearly be seen that the most significant metabolites which are elevated in the non-age corrected model (Figure 1) are also present and the most highly significant in the age corrected model (Figure S2). These include formic acid (*p*-value of age corrected model = 3.16 × 10^−29^), pyruvic acid (*p* = 2.46 × 10^−16^), H2TG (*p* = 1.61 × 10^−14^), LDTG (*p* = 3.67 × 10^−12^), aspartic acid (*p* = 2.65 × 10^−14^), and glutamic acid (*p* = 2.37 × 10^−13^). While the same lipids are still most significantly decreased in the COVID-19 patients, PC 18:1/18:2 (*p*-value of age corrected model = 5.51 × 10^−25^), PC 18:2/18:2 (*p* = 7.07 × 10^−24^), PE.P 18:1/18:2 (*p* = 4.58 × 10^−22^) and PE.P 16:0/18:2 (*p* = 5.48 × 10^−22^).

The observation of exceptionally high serum concentrations of pyruvate in COVID-19 patients is concordant with existing literature on SARS-CoV-2 infected individuals, with some studies showing a correlation with severity [34,35]. This strong correlation of pyruvate with SARS-CoV-2 infection is consistent with reported altered mitochondrial function (failure to utilize pyruvate as an energy source) following viral infection, which can trigger an immune response that shifts towards aerobic glycolysis to increase production of fatty acids, amino acids, and nucleotides [29]. Increased circulating hypoxia-inducible factor-1α (HIF-1α), which induces glycolysis [36], has been reported in SARS-CoV-2 infected patients, as has lactate [37]. Disruption of oxidative phosphorylation has been independently observed via transcriptomic measurements in COVID-19 [38] and further studies on the dynamics of mitochondrial disruption during COVID-19 are warranted.

Whilst increased serum pyruvate could result from a dysregulation of mitochondrial metabolism or hepatic central carbon metabolism [35], another theory is that the increase in pyruvate concentrations is driven by an increase in lactate dehydrogenase activity [39]. Zhou et al. [39] reported that the lactate to pyruvate ratio, which reflects lactate dehydrogenase activity, was markedly lower in the infected group in comparison to the non-infected group. Marin-Corral et al. [34] published similar findings and proposed that since the lower lactate to pyruvate ratio in SARS-CoV-2 infection was not associated with a concomitant increase in serum lactate concentrations, the high pyruvate concentrations may rather reflect an imbalance of nicotinamide adenine dinucleotide (NAD) metabolism, which is required to convert pyruvate into lactate. In support of this hypothesis, they found evidence of alteration in other metabolite ratios that require NAD+ as a cofactor in SARS-CoV-2 patients including the transformation of cortisol into cortisone by 11β-hydroxysteroid dehydrogenase type 2 [34]. The synthesis of NAD+ is dependent on the kynurenine arm of the tryptophan pathway [40], which is also disrupted following SARS-CoV-2 infection. We also found a significantly lower lactate to pyruvate ratio in infected patients (*p*-value = 6.10 × 10^−35^). However, although the infection-related increase in pyruvate and decrease in the lactate:pyruvate ratio was amongst the strongest differentiators of non-infected and infected samples, we did not find a direct relationship with severity indicating a quantized shift of metabolic state associated with infection.

We found glutamate and aspartate to be directly associated with SARS-CoV-2 infection, whereas glutamine and asparagine were moderately but significantly (Table S5) inversely associated. Therefore, we calculated the ratio Asp:Glu/Asn:Gln to summarize these observations and found it to be strongly associated with the severity of infection. Other studies have also shown that SARS-CoV-2 infection results in significantly enriched aspartate and glutamate metabolism [3,28,41], and that impaired glutamate and glutamine pathways were the strongest metabolic indices of SARS-CoV-2 infection [42], with some proposing glutamine supplementation as part of the therapeutic management of the infection [43]. However, some research groups have reported lower plasma glutamate concentrations in SARS-CoV-2 infected individuals [44]. On balance the literature and our results from the current study indicate that the glutamate to glutamine ratio is strongly associated with SARS-CoV-2 infection [45]. Both aspartate and glutamate are major anaplerotic carbon sources for the citric acid cycle, and they may be another indication of reduced mitochondrial efficiency during SARS-CoV-2 infection. Krishnan et al. reported higher levels of serum glutamate and found that the surface expression of the glutamate transporter xCT (SLC7A11) was increased in monocytes in SARS-CoV-2 infected patients and showed that glutaminolysis was essential for replication of the SARS-CoV-2 virus [46]. There has been considerable concern about reports of new onset diabetes caused by SARS-CoV-2 infections [3,47,48]. It is of note that high plasma glutamate and low plasma glutamine observed here and in earlier work [3] is a strong feature in the plasma during the acute phase of the disease. We previously reported that the glu/gln ratio remained persistently high in patients following COVID-19 and was one of the least reversible of the metabolic features measured in “long COVID” patients [2]. This indicates a persistent driver for type 2 diabetes in post COVID-19 patients and warrants further investigation in relation to long-term diabetic risks.

We also found elevated plasma Ornithine in infected patient samples which may indicate upregulation of the urea cycle, possibly driven by the increase in serum aspartic acid. Upregulation of urea cycle metabolites was also reported by Costanzo et al. [6,28]. Elevated ornithine levels have been associated with an increase in ammonia burden due to a metabolic block in the urea cycle. In such cases, the ammonia burden normally shifts the glutamate:glutamine ratio towards glutamine. However, this is the reverse of what we observed here with a highly significant shift towards glutamate. Recent large-scale epidemiology studies have shown that a high glu:gln ratio is associated with type 2 diabetes and risk of metabolic diseases [48,49]. Higher plasma concentrations of glutamate, lower glutamine concentrations, and the associated higher glutamate:glutamine ratio have been associated with increased risk of type 2 diabetes in the PREDIMED trial. Proposed mechanisms by which this altered glutamate to glutamine ratio impacts diabetes risk includes the fact that glutamine can bring about lowering blood glucose levels by stimulating insulin secretion via release of the glucagon-like peptide (GLP-1) [50]. Conversely, high circulating concentrations of glutamate can increase oxidative damage in pancreatic cells [51]. This is of interest because of the diabetogenic properties of the SARS-CoV-2 infection and the increased diabetes risk that is now recognized as a problem associated with long COVID [48].

Of all the metabolites in the predictive molecular panel for infection, formate demonstrated the strongest association with SARS-CoV-2 infection [9,52,53]. Formate is formed as a by-product of the conversion of tryptophan to *N*-formyl-kynurenine and one of the metabolic hallmarks of SARS-CoV-2 infection is the reduction of the bioavailability of tryptophan through the activation of Indoleamine 2,3-dioxygenase (IDO), in-turn reducing serotonin levels and elevating production of kynurenine and quinolinic acid products [6,54]. However, formate is also formed as part of gut microbial metabolism, and it is known that the gut microbiome can be significantly impacted by SARS-CoV-2 infection and so this potential biomarker is likely to have multiple origins directly and indirectly related to the virus infection.

The parameters were ranked according to their significance in the overarching multi-class severity model, and the top 50 parameters, mostly but not exclusively, belong to the lipid and lipoprotein classes (Figure S1). Radar plots were constructed to show the fold-changes between the control samples and the SARS-CoV-2 positive patients for the 50 most significant molecules by *p*-value; the radar plots were ordered clockwise by decreasing fold change (Fold change is defined as (B − A)/A, so 0 means no change). Pyruvate shows the largest fold-change between the controls and the SARS-CoV-2 positive patients. The lipids that were most significant in differentiating infected from non-infected samples were the phosphatidylcholines (PC 18:2/18:2, PC 18:1/18:2, PC 18:2/20:4) and phosphatidylethanolamines (PEP 18:2/18:2, PEP 18:1/18:2, PEP 16:0/18:2). This has been reported previously but was based on a study with a smaller number of participants [22]. Several of the HDL subclass-4 lipoprotein parameters (H4A1, H4A2, H4CH, and H4PL) were markedly reduced and featured in the most significant list. Previous studies have found an inverse correlation between levels of HDL particles and severity of SARS-CoV-2 infection, with some studies showing binding between the spike protein of the virus and HDL [4,55]. In contrast, other studies have proposed that HDL facilitates infection of host cells by binding to angiotensin-converting enzyme 2 (ACE2) [56]. In one study, low pre-covid levels of HDL cholesterol was found to be correlated with the severity of SARS-CoV-2 infection [57].

In previous research on pulmonary arterial hypertension, low plasma levels of HDL4 were found to be associated with mortality [58]. Plasma concentrations of HDL4 levels were directly associated with several proteins involved in fibrinolysis and indeed small HDL particles such as HDL4 are known to transport proteins such as prekallikrein [58]. Prekallikrein is the precursor of the serine protease kallikrein which acts to release kinins such as bradykinin, which are involved in fibrinolysis, blood pressure control, and vascular inflammation [59]. In addition to atheroprotective properties and a role in fibrinolysis, HDL4 (also referred to as HDL3b and HDL3c in older nomenclature) has been shown to have antioxidant and antiinflammatory properties, with the ability to stimulate production of nitric oxide, mainly due to the effect of Apo-A1 [60]. The highest ranked lipoprotein parameter in differentiating SARS-CoV-2 infection and control and which was also lower in the participants who died was H4A1 (HDL4-Apolipoprotein-1), the main protein carried by the small, dense HDL4 particles. Apo-A1 is inversely correlated to cardiovascular disease and is arguably a better predictor of cardiovascular disease [61].

Stratification of the data by molecular class or assay type allows a more detailed assessment of the impact of infection. The models for each assay type are provided in Figure S3. While the model built using all four assays yielded a AUROC of 0.99 for differentiating infected from non-infected samples, the models generated for lipoproteins, lipids, and low molecular weight metabolites independently also showed excellent classification predictivity (AUROC 0.95–1.00), indicating that any one of these assays on its own was capable of accurately classifying SARS-CoV-2 infection (Table 1 and Figure S3).

### 2.2. Metabolite Classes Based on Severity of Infection

**Figure 2:**
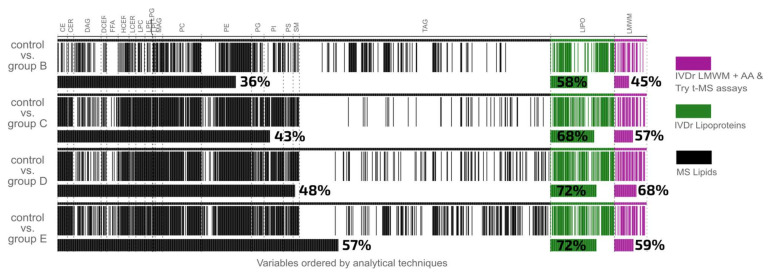
Metabolic Barcode Diagram illustrating the Pan-Metabolic statistically significant lipids, lipoproteins, low molecular weight metabolites, amino acids, and tryptophan pathway intermediates for each respiratory severity group versus the controls. The 1034 quantitative variables are ordered by analytical technique, lipids (black), lipoproteins (green), and low molecular weight metabolites and MS amino acids and tryptophan (magenta). White spaces on the plot indicate metabolites that are not significant.

**Figure 3:**
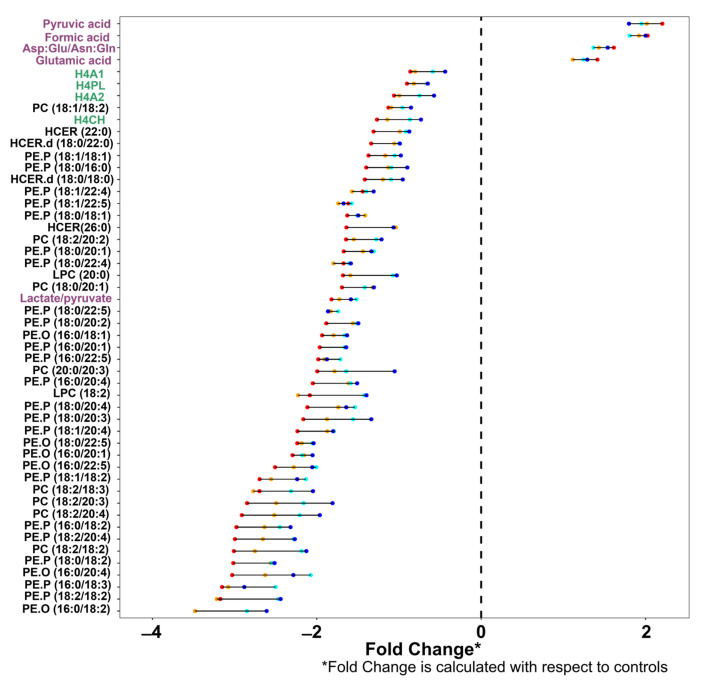
Pan Metabolic Severity Mapping of COVID-19 Severity—the top 50 most significant differentiating metabolites in the controls versus each severity group models for the integrated data set. The top 50 most significant metabolites by *p*-value of the integrated data set using the controls vs. SARS-CoV-2 positive patients, ordered by log_2_ fold change. Fold changes with respect to controls of each severity class are shown: group B (blue), group C (cyan), group D (orange), and group E (red). The metabolite axis is colored according to the assay with which the metabolite is measured: lipids (black), lipoproteins (green), and the low molecular weight metabolites (magenta).

The key question to consider is whether the metabolite classes that are most significantly perturbed within a mild case of COVID-19 disease are the same as those in an individual with a severe infection. To obtain a more comprehensive view of all the 1034 variables in the integrated model and how the significant metabolites change with increasing respiratory severity, significant metabolites were clustered and colored by assay in a pan-metabolic plot (Figure 2): lipids in black; lipoproteins in green; low molecular weight metabolites in magenta. For all three molecular panels, the core differential metabolites remained the same across severity categories, with the ranked order of significant metabolites being similar, but not identical across the severity levels (Figure 2 and Figure 3, Tables S6–S9). The similarity of the core metabolites across all severity models indicates that the metabolic changes within the severity group B are similar to those in group E, at least within the top 50 most significant metabolites. However, it can be clearly observed that group E severity log_2_ fold change is greater than group B, C, and D (Figure 3 and Figure S1B). Although the core set of metabolites were stable as severity progressed, as the severity level of respiratory symptoms increased, the number of discriminatory metabolites in the O-PLS-DA models tended to increase incrementally (Table 2). Thus, the model for the mildest severity (group B) contained the lowest number of statistically significant parameters (404 in Group B, 483 in Group C, 537 in Group D, and 608 in Group E, respectively).

Expressed as a percentage of the total measured parameters, it is evident that the greatest increment in percentage of differential parameters is observed between SARS-CoV-2 participants who did not require hospitalization (Group B) and those who required hospitalization but with no requirement for oxygen (Group C). For lipids, the percentage of statistically significant differential parameters ranged from 36% of all lipids measured being discriminatory in the non-hospitalized category to 57% in the most severe category, indicating that even at the lowest severity there is substantial metabolic dysregulation. To display the pan-metabolic responses to different severity levels, we have introduced a “Metabolic Barcode” model (Figure 2). Here, each line of the barcode represents an individual statistically significant parameter organized according to molecular class providing a means for rapid visualization of similarities between the models, in this case relating to increasing severity. Thus, it can be seen that the lipoproteins and most of the lipid classes share strong similarities across severity levels but that the triacylglycerides are somewhat different between the least and most severe class comparisons with control. For the low molecular weight metabolites and the lipoproteins, the number of significant metabolites increased most sharply between categories B (non-hospitalized) and C (hospitalized but not requiring oxygen) but plateaued at the more severe symptom categories. Since the decision whether to administer oxygen can be partially dependent on the caregivers, there is a certain amount of subjectivity in the distinction between hospitalized patients who did or did not receive additional oxygen, and therefore the similarities between categories C and D is not unduly surprising.

Another type of pan-metabolic response graph is shown in Figure 3 where the top 50 metabolites differentiating control from SARS-CoV-2 infected classes are ranked by order of the variables in terms of their adjusted *p*-values in each of the pairwise comparisons of the severity groups with non-infected controls for the integrated parameter set, and shows the log_2_ fold change with respect to the non-infected control class, with the different severity classes defined by the color of the coordinate (red for the greatest severity and blue for the least severe). Thus, the longer the bar, the greater the difference in fold change from the least to most severe class. The top 25 parameters differentiating infected and control samples on a class-by-class basis are provided in Table 2, which shows that in general, while there is some reordering in the rank of significant metabolites (based on adjusted *p*-value) level of significance of the metabolites as severity increases (Table 2), the same metabolites remain upregulated in the disease state. Formate is the most significant metabolite across all severities, except for Group E, where it falls to second place behind the neopterin:tryptophan ratio. Pyruvate and the Asp:Glu/Asn:Gln are consistently ranked in the top four places across all severity levels. It should also be noted that for the control vs. B group (Table 2), most of the top 25 significant metabolites comprise mainly of lipids but as severity increases a number of ranked HDL subparticle four lipoprotein classes are involved. This is of note because we have previously shown that HDL subclass four (the smallest high density HDL) is significantly reduced in COVID-19 [4,20]. HDL is also reduced in pulmonary hypertension and carries several fibrinolytic proteins, such as alpha-2-antiplasmin, prekallikrein, and coagulation factor XI [58], which potentially reflects a predisposition towards micro blood clotting, a known problem of SARS-CoV2 infection. In the higher severity groups, TPA1 and TPA2 (total Apolipoprotein-A1 and -A2) were significant in differentiating between cases and controls in addition to H4A1 and H4A2, emphasizing the roles of Apolipoprotein-A1 and -A2 in response to SARS-CoV-2 infection.

Inspection of the extended list of the top 50 most significant parameters (Figure 3 and Table 2) shows that lipids dominate the ranked lists with lower levels of multiple phosphatidylcholines and phosphatidylethanolamines in the infected group being a defining feature. Although the core molecular signature of SARS-CoV-2 infection was similar in character regardless of the severity, the main exception lay in the impact on the tryptophan pathway, which was differentially impacted in the most severely infected group (Table 2).

For most parameters, the difference in magnitude of fold change between the least (Group B, blue) and most (Group E, red) severe infection groups are not substantial and many parameters do not demonstrate a linear progression with severity, again reinforcing the observation that the molecular signature of the infection is similar regardless of respiratory severity. For example, in severity groups B and C, pyruvic acid, Asp:Glu/Asn:Gln, and glutamic acid manifest the largest fold change from non-infected but the fold change does not increase substantially as the respiratory severity of infection increases. This suggests changes in these metabolites and ratios may be more indicative of the presence of infection. The exception is for serum levels of tryptophan, neopterin, and quinolinic acid (Figure S5), which show an abrupt concentration change with the transition from groups B and C to groups D and E thereby associating with severity rather than presence of infection. Nevertheless, with the exception of the contribution from the tryptophan pathway metabolites, the severity of respiratory infection has little impact on the core metabolic parameters differentiating infected from non-infected individuals. To note, within severity group E, the assisted ventilation patients, 22 were admitted into ICU while 35 were not. Comparison of the 1034 fully quantified metabolic variables of those who were admitted into ICU versus those who were not where all patients were subsequently discharged from hospital resulted in all adjusted *p*-values being non-significant. Figure 3 and Table 2 shows some of the same metabolites that are highly significant are the same between controls versus mild cases and controls versus assisted ventilation patients. This suggests the shifts in metabolomic profile seen are highly dependent on the presence of infection with minimal contributions from assisted ventilation and admission into ICU. These findings would indicate that the metabolic monitoring of patients with mild acute phase respiratory disease may be equally important as a patient that was severely ill in the acute phase. Indeed, it has been shown previously that even with mild symptoms, metabolic perturbations are still present many months after the acute phase of the disease is resolved [2], and several of these metabolic perturbations may be associated with altered long-term disease risk.

When the datasets for the assays were modelled independently and stratified for respiratory severity, all four infected categories ranging from non-hospitalized but symptomatic (Group B) to hospitalized and requiring ventilation (Group E) when compared with the non-infected group delivered robust models (Table 1). O-PLS-DA scores plots, eruption plots, and variable importance plots of the variables of each model can be found in the Supplementary Materials (Figures S6–S12 and Tables S10–S29). In most cases, the AUROC values were as high for the model comparing the mild severity disease with the non-infected group (AUROC= 0.98–1.00) as for the model of the most severe disease group versus the non-infected group (AUROC > 0.99). Although the metabolic plasma profiles showed a continuum of changes across the respiratory severity levels, even mildly affected respiratory patients showed multiple highly significant abnormal biochemical signatures reflecting serious metabolic deficiencies of the type observed in Post-acute COVID-19 syndrome patients.

For the lipoprotein dataset, strong models were produced for each severity classification, which gives insights into cardio-metabolic complications of COVID-19 (Figures S6–S8). Seven of the top ten lipoproteins appear as the most significant in each of the four severity comparisons, namely: H4CH (high density cholesterol subfraction 4), H4PL (high density phospholipid subfraction 4), TPA2 (Apolipoprotein-A2), H4A1 (high density lipoprotein Apolipoprotein-A1 subfraction 4), H4A2 (high density lipoprotein Apolipoprotein-A2 subfraction 4), HDA1 (high density lipoprotein Apolipoprotein-A1), and H4FC (high density free cholesterol subfraction 4), which are all decreased in the SARS-CoV-2 infected individuals. As before, the number of statistically significant metabolites increased as the respiratory severity of the patient worsened (Table 1 and Table 2). Low Density Triglyceride (LDTG) had the greatest statistical significance across all severity classes. Interestingly, ABA1 (Apolipoprotein-B100/Apolipoprotein-A1), a known cardiovascular risk lipoprotein marker [1,31,62,63], demonstrated an increase in all severity classes in comparison to the controls with a Cliff’s delta ranging from 0.91–1.00, which indicates that even with mild disease the potential for detrimental cardiovascular effects is present.

Pairwise comparison of SARS-CoV-2 infected groups indicated that severity classes B and C could not be significantly differentiated for any of the molecular panels with AUROC < 0.6 for all single increment models (Table 1). Similarly, groups C and D and groups D and E could not be differentiated based on their lipid or lipoprotein parameters, although weak models were obtained for the low molecular weight parameter set for the comparison of group C (hospitalized patients with no oxygen supplementation) versus severity group D (hospitalized patients with low flow oxygen) and for group D versus E. As a result, the number of differential metabolites for each assay was vastly reduced when comparing between severity levels in comparison with comparing any of the infected classes to those non-infected. This lack of ability to robustly differentiate sequential severity classes supports the suggestion that metabolically the impact of SARS-CoV-2 infection is much greater than differences between mild and severe infection.

As shown in the Eruption plots constructed from the lipid data (Figure S9), the majority of the lipid parameters are reduced in SARS-CoV-2 infection from the mildest infection to the most severe. However, from the lipids that increase with infection, monoacylglycerol 20:3 is the most significantly upregulated lipid in severity group B vs. controls and severity group C vs. controls and remains highly significant in the more severe disease states. Phosphoserine 18:0/18:0 is also highly significant in all models, while ceramide 18:0 increases in significance as respiratory severity increases. Interestingly, of the lipids that decrease with COVID-19 infection, the most significant in the control vs. B group comparison is also the most significant in the control vs. E group: phosphocholine 18:1/18:2. In all the severity classes vs. controls the phosphocholine, phosphoethanolamine, lysophosphocholine, and hexosylceramide remain the most significant markers of COVID-19 infection, regardless of severity class, all of which are present in reduced concentration compared to the controls and have been reported to have been reduced previously in COVID-19 infections [5,64].

### 2.3. Tryptophan Pathway Metabolism Is Substantially Disrupted in Severe SARS-CoV-2 Infection

Although most of the low molecular weight metabolites, including many of the amino and organic acids, did not associate with infection severity, we and others have reported the tryptophan pathway to be disrupted by SARS-CoV-2 infection [6] and also influenced by the severity of infection: quinolinic acid (positively associated), tryptophan (negatively associated), and 3-hydroxykynurenine (positively associated), correlated with severity (See Figures S11–S13). Indole-2,3–dioxygenase modulates the production of kynurenines and is a known regulator of inflammation in the event of infection, including SARS-CoV-2 [65]. In the integrated set of all metabolites stratified by severity, no tryptophan pathway metabolites ranked in the top 50 most discriminatory parameters when comparing either group B or C severity with control, whereas the neopterin to tryptophan ratio (positively associated) ranks in the top 50 for Groups D versus control and both the neopterin to tryptophan and quinolinic acid to tryptophan (positively associated) ratios rank in the top 50 most significant parameters for the comparison of group E versus controls (Table S9). Low tryptophan levels together with high neopterin concentrations have been associated with cardiovascular disease and cancer [66] and are related to inflammation. Analysis of the low molecular weight parameter set in isolation additionally finds higher serum concentrations of quinolinic acid, neopterin, and 3-hydroxykynurenine, plus lower levels of tryptophan and serotonin (Tables S21–S24). Lower serum serotonin levels are only apparent in the models for the two highest severity categories.

Disruption of the tryptophan pathway following SARS-CoV-2 infection has been reported in multiple studies [2,6]. Both quinolinic acid and 3-hydroxykynurenine, together with glutamate, which is also directly associated with infection across all severity classes (Tables S21–S24), are excitatory neurotoxins [67]. Increasing reports of associations between quinolinic acid and neurodegeneration include conditions such as Huntington’s disease, AIDS, dementia, Alzheimer’s disease, and Parkinson’s disease [67,68,69]. In the current study, changes in serum concentrations of the kynurenine:tryptophan ratio and picolinic acid were found to track with the severity of SARS-CoV-2 infection. Consistent with this observation, kynurenine significantly differentiates classes C to E from controls but not B whereas picolinic acid is only significant on the model comparing the most severe class E with non-infected (Tables S21–S24). Upregulation of the kynurenine pathway occurs due to proinflammatory cytokines including IL-1, TNF-α and IL-6 [70] and has been noted previously in COVID-19 and other chronic diseases [71].

These data indicate that as the severity increases, catabolism of tryptophan via the kynurenine pathway increases [33]. Picolinic acid, a metabolite downstream in the pathway, is the most significant metabolite in the D vs. E model while it was not significant in the C vs. D model and has been previously shown to have a role in inflammatory disorders within the central nervous system [72] and to be increased in cases of children with malaria [73]. It has also been found to be significant in severity classification in COVID-19 patients. Cihan et al. reported an association between SARS-CoV-2 infection severity and picolinic acid and the kynurenine:tryptophan ratio and showed a correlation between KYN:TRP and the inflammatory marker IL-6 [33]. It has been shown previously that decreases in tryptophan concentrations become significant in severely ill COVID-19 patients in comparison to mild or moderately ill patients [38,74].

### 2.4. Mortality Prediction in SARS-CoV-2 Positive Patients

This study was conducted early in the pandemic with the Wuhan sub-variant dominant in an exclusively unvaccinated population with consequent high mortality. Within the severely ill SARS-CoV-2 patients (group E severity), 56.1% died. No patients in severity groups B, C, or D died. In terms of demographics, the subset of group E that did not survive were significantly older than those that survived. In order to remove the effect of the age disparity and compare the two groups, only participants that were between the ages 65–80 were selected, resulting in 13 people who survived (median age = 73) and 13 who did not (median age = 75). The demographics of these two groups are found in Table S30.

**Figure 4:**
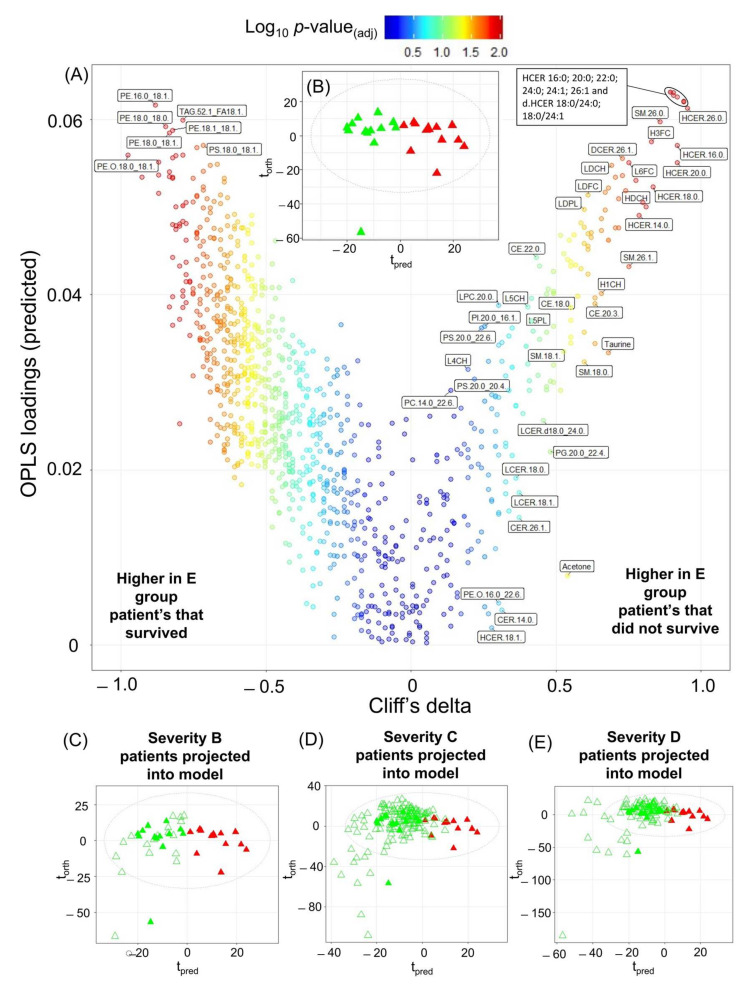
Modelling Pharmaco Metabonomic COVID-19 mortality prediction on the integrated dataset. (**A**) Eruption plot of the severity group E patients who survived vs. the severity group E patients who died. (**B**) O-PLS-DA of severity group E patients who survived (green triangles) and severity group E patients who died (red triangles), R_2_X = 0.26, AUROC = 0.96. Cliff’s delta, OPLS loadings values and the adjusted *p*-values for this model can be found in Table S31. Model was built using 1034 parameters. (**C**) O-PLS-DA of severity group E patients who survived (green closed triangles) and severity group E patients who died (red closed triangles) with group B severity patients projected onto the model (green open triangles). (**D**) O-PLS-DA of severity group E patients who survived (green closed triangles) and severity group E patients who died (red closed triangles) with group C severity patients projected onto the model (green open triangles). (**E**) O-PLS-DA of severity group E patients who survived (green closed triangles) and severity group E patients who died (red closed triangles) with group D severity patients projected onto the model (green open triangles).

For the 13 SARS-CoV-2 infected patients, it should be noted that the time between blood collection to the patient dying ranged from 8–61 days. The resulting model (Figure 4), which used all the 1034 variables from all four assays, had an AUROC of 0.96 indicating that the retrospective model was able to predict mortality at a median of 25.5 days prior to death. All significant metabolites can be found in Table S31. The severity classification prediction model was then cross validated. Projection of the COVID-19 patients from this study into the trained model provided high specificity. For the group B severity group (Figure 4C), all were classified as survivors, so the specificity = 1.00. For group C severity (Figure 4D), the specificity = 0.87, McNemar’s Test *p* = 6.15 × 10^−5^. For group D severity (Figure 4E), the specificity = 0.89, McNemar’s Test *p* = 4.43 × 10^−3^.

Effectively this means that on hospitalization, the high mortality patients had a pharmaco-metabonomic serum signature that was predictive of the outcome of the disease for up to 25 days prior to death. The concept of pharmaco-metabonomic prediction was proposed to define the ability to predict metabolic outcomes based on pre-intervention or pre-disease metabolic profiles [75,76], and has previously been applied to retrospectively predict survival in acute-on-chronic liver disease patients [77]. Knowledge of such prognostic data could be applied to beneficially influence selecting the therapy of the individual patient.

As shown in Figure 4A, the lipid family hexosylceramides were key indices of survival and several ceramides were present in significantly higher levels (Cliff’s delta > 0.5) in the patients who did not survive (hexosylceramide 16:0; 20:0, 22:0; 24:0; 24:1; 26:0; 26:1 and dihydrohexosylceramide 18:0/24:0; 18:0/24:1). An increase in ceramide species in those patients that did not survive has previously been shown in a study predicting the 7-day mortality outcome [78,79]. It has also been demonstrated that ceramides could predict death in patients with stable coronary artery disease and acute coronary syndromes, where it was postulated that the ceramides are associated with lipoprotein aggregation and uptake, superoxide anion production, apoptosis, and inflammation [80]. More specifically, hexosylceramides have been found in higher concentrations in patients with multiorgan dysfunction syndrome than sedated controls in an intensive care unit [81] and have also been linked to viral load in hepatitis C infection [82].

In addition to the elevated hexosylceramides, three sphingomyelins with sidechain lengths 20:1, 26:0, and 26:1 (Cliff’s delta values 0.70, 0.86, and 0.75, respectively) were also elevated in the patients that did not survive. Increases in sphingomyelins have been shown previously in COVID-19 infection in humans and in animal models [83]. In contrast to hexosylceramides and sphingomyelins, phosphoethanolamines with chain lengths 16 and 18 and the triacylglycerides are decreased in the patients who did not survive. Correlation plots were completed (Figure S14) and showed differing patterns between the two groups in the model.

Using the combined panel of lipids, lipoproteins, and small molecules, the AUROC for predicting survival was 0.96. This compares well with other previously published predictive biomarkers and biomarker panels. In terms of single predictive clinical markers of survival, placental growth factor (P1GF) returned an AUROC of 77.2% for predicting survival at a median of 14 days after hospital admission (range 2 to 57 days) [84] and CRP concentrations correlated with 14-day mortality with a sensitivity of 0.88 and specificity of 0.56 [85], chromogranin A [86], and D-dimer [87]. In particular, D-dimer levels along with high sensitivity CRP, ferritin, and IL-6 have been reported to be correlated with the severity of SARS-CoV-2 infection with D-dimer demonstrating the best ability for predictions of mortality [88]. Other research groups have proposed ratios of biomarkers for predicting SARS-CoV-2 mortality such as (kynurenine/tryptophan)/(cirulline/ornithine), which returned an AUROC of 0.95 [89]. Although various biomarker panels have been proposed with relatively high sensitivity and specificity, many of these have not been validated in other cohorts. One example is a panel of lactate dehydrogenase, CRP, and lymphopenia which achieved >90% accuracy in predicting mortality in a Chinese cohort but was not replicated in a cohort of Caucasian Dutch individuals [90].

In the current cohort, we found altered serum lipoproteins also contributed to the model differentiating patients who did and did not survive (Figure 4 and Figure S15). Key changes were observed in predominantly cholesterol and free cholesterol components. These include HDCH (Cliff’s delta = 0.80), LDCH (Cliff’s delta = 0.69), H3FC (Cliff’s delta = 0.83), HDFC (Cliff’s delta = 0.70). Total Apoprotein A1 (TPA1, Cliff’s delta = 0.60), HDL Apoprotein A1 (HDA1, Cliff’s delta = 0.61), and HDL subfraction 4 Apolipoprotein A1 (H4A1, Cliff’s delta = 0.61) are all increased in the patients who did not survive. The very low density lipoprotein fractions were present in higher concentrations in those who survived compared to those who did not. These observations are in concordance with the work from Masana et al. [91] who noted that low plasma HDL cholesterol and high triglyceride concentrations were correlated with infection severity. Similarly decreased plasma concentrations of several lipid classes has been reported as a feature of SARS-CoV-2 with lysophosphocholine (LPC) 18:0 and LPC 18:2 being inversely correlated with mortality. Although we did not find a specific correlation between these lipids and survival in the current cohort, LPC 18:0 and LPC 18:2 were associated with both the presence of SARS-CoV-2 infection and severity (Tables S5, S17 and S19) [92]. Given the predictive strength of the lipid data, we assessed the ability of the top lipid species defining the SARS-CoV-2 positive and control groups according to the adjusted *p*-value (HCER 16:0 and PE.O 18:0/18:1), respectively, to predict mortality. The AUROC based on these two lipid species was 0.99 (Supplementary Figure S16) suggesting that this may be a good diagnostic for SARS-CoV-2 mortality and that the diagnostic value of these lipids warrants validation in independent datasets.

The only low molecular weight metabolite with a Cliff’s delta above 0.6 in the model predicting mortality was taurine which was higher in the patients that did not survive. The Mann-Whitney test showed the significance between the two groups to be 2.3 × 10^−3^. Elevated taurine levels have previously been associated with liver injury and hepatotoxicity [93]. However, taurine is also present in large quantities in skeletal and cardiac muscle [94], and as COVID-19 causes skeletal muscle loss it may be a result of muscle breakdown [95]. Other groups have previously achieved accurate mortality prediction using clinical [96,97] and metabolomic [38,89,98] data only. Here we present a model which contains a larger patient cohort, therefore adding statistical power, and measuring considerably more variables facilitating deeper understanding of mechanistic pathways involved in SARS-CoV-2 infection.

## 3. Materials and Methods

### 3.1. Participant Enrolment and Sample Collection

The cohort consisted of non-infected control participants (*n* = 89) and patients who tested positive for SARS-CoV-2 infection from upper and/or lower respiratory tract swabs by RT-PCR (*n* = 306). These samples were collected early in the pandemic with the Wuhan sub-variant dominant in an exclusively unvaccinated population. The infected participants were divided into four categories based on severity of respiratory symptoms: severity group B, symptomatic but no hospitalization; group C hospitalized, no oxygen required; group D hospitalized supplemental oxygen required; and group E hospitalized, assisted ventilation [21]. No asymptomatic patients were in this cohort (group A). The cohort demographics are provided in Table S1.

All serum samples were provided by the Basque Biobank for research (BIOEF). Control serum samples were collected prior to the COVID-19 pandemic by Osarten Kooperativa Elkartea from an apparently healthy population (employees of the Mondragon Cooperative [Basque Country], during the annual medical test). For the control samples only the participant gender, age, and BMI were provided for this study (Table S1). No information was provided upon the possible presence of any other diseases such as diabetes or cardiovascular disease. For this reason, within this study, they are not referred to as healthy controls but controls of a normal population.

The COVID-19 samples were collected at the Cruces University Hospital (Barakaldo, Spain) from patients who presented compatible symptoms, confirmed by a RT-PCR assay on nasal swab samples. All blood was collected in BD vacutainer serum tubes with clot activator with the same pre-analytical handling procedures for the controls and patients. All participants provided informed consent, according to the Declaration of Helsinki, and data were anonymized to protect their confidentiality. The sample handling protocol was evaluated and approved by the ethics committee of Basque Country (Report of the ethics committee for research on medicinal products in the Basque Country, CEIm-E, PI+CES-BIOEF 2020-04, and PI219130). Shipment of human samples to the ANPC had the approval of the Ministry of Health of the Spanish Government and were imported under Import Permit 0004275122 issued by the Australian Government Department of Agriculture, Water, and the Environment. Upon receipt samples were stored at −80 °C. Samples were approved for analysis as part of the International Severe Acute Respiratory and Emerging Infection Consortium (ISARIC)/World Health Organization (WHO) pandemic trial framework (SMHS Research governance office PRN:3976). Research was conducted in accordance with the Murdoch University Human Ethics Committee approval (no. 2020/052 and 2020/053).

### 3.2. ^*1*^H NMR Spectroscopy Sample Preparation

All sample preparation and processing followed the guidelines recommended by Loo et al. [99]. Samples were defrosted at room temperature for 1 h prior to preparation for analysis. NMR samples were prepared in a SamplePro Tube (Bruker Biospin, GmbH, Ettlingen, Germany) robot system for liquid handling with integrated temperature control. Every sample was automatically prepared as a mixture of phosphate buffer (75 mM Na_2_HPO_4_, 2 mM NaN_3_, 4.6 mM sodium trimethylsilyl propionate-[2,2,3,3-2H_4_] (TSP) in H_2_O/D_2_O 4:1, pH 7.4 ± 0.1) and serum at a 1:1 ratio for a final volume of 600 μL into the 5 mm SampleJet^TM^ NMR tubes. Samples were then manually shaken for several seconds and stored at 5 °C inside the SampleJet^TM^ automatic sample changer until measurement (<24 h). All methods were validated for COVID-19 samples as previously reported [99].

### 3.3. ^*1*^H NMR Spectroscopy Data Acquisition and Processing Parameters

NMR spectroscopic analyses were performed on a 600 MHz Bruker Avance III HD spectrometer, equipped with a 5 mm BBI probe and fitted with the Bruker SampleJet^TM^ robot cooling system set to 5 °C. A full quantitative calibration was completed prior to the analysis using a protocol described elsewhere [100]. All experiments were acquired using the Bruker In Vitro Diagnostics research (IVDr) methods. For each sample prepared, a standard 1D experiment with solvent pre-saturation (32 scans, 98K data points, spectral width of 30 ppm) amounting to a total experiment time of 4 min 3 secs was generated and a total of 112 lipoprotein parameters were measured [18] using the Bruker IVDr Lipoprotein Subclass Analysis (B.I.-LISA^TM^) method whereby the –(CH_2_)n at δ = 1.25 and –CH_3_ at δ = 0.80 peaks of the 1D spectrum after normalization to the Bruker QuantRef^TM^ manager within Topspin^TM^ were quantified using a PLS-2 regression model. B.I.LISA. Parameters measured consisted of total serum lipid analytes cholesterol, free cholesterol, phospholipids, triglycerides, Apolipoproteins A1/A2/B100 and the B100/A1 ratio, and analytes distributions in different density classes of serum-lipoproteins: high-density lipoprotein (HDL, density 1.063–1.210 kg/L), intermediate-density lipoprotein (IDL, density 1.006–1.019 kg/L), low-density lipoprotein (LDL, density 1.09–1.63 kg/L), and very low-density lipoprotein (VLDL, 0.950–1.006 kg/L). The main lipoprotein classes HDL, LDL, VLDL were subdivided into different density sub-classes. LDL subdivisions included: LDL-1: 1.019–1.031 kg/L, LDL-2: 1.031–1.034 kg/L, LDL-3: 1.034–1.037 kg/L, LDL-4: 1.037–1.040 kg/L, LDL-5: 1.040–1.044 kg/L, LDL-6: 1.044–1.063 kg/L). HDL sub-fractions were also assigned to 4 density classes: HDL-1 1.063–1.100 kg/L, HDL-2 1.100–1.125 kg/L, HDL-3 1.125–1.175 kg/L, and HDL-4 1.175–1.210 kg/L and the VLDL sub-fractions were divided into five density classes. A list of all the 112 lipoprotein subfractions and parameter annotations are shown in Table S2. In addition to the 112 lipoprotein parameters, 11 low molecular weight metabolite concentrations were obtained from the Bruker IVDr Quantification in Plasma/Serum B.I.Quant-PS (acetic acid, acetoacetic acid, acetone, citric acid, creatine, creatinine, formic acid, glucose, D-3-hydroxybutyric acid, lactic acid, pyruvic acid) (Table S3).

### 3.4. Liquid Chromatography Mass Spectrometry (LC-MS)

Biogenic amines, amino acids, and tryptophan metabolites were measured using two LC-MS quantification methods following previously reported methods for tryptophan and associated catabolites [101] and amino acids [19,102], which were used to measure forty-five parameters (thirty-six individual metabolite concentrations and nine ratios, Table S2). In brief, samples were thawed at 4 °C and prepared for analysis. For the quantification of the biogenic amines and amino acid metabolites, a Bruker Impact II QToF mass spectrometer (Bruker, Daltonics, Billerica, MA, USA) coupled to a Waters Acquity I-class UPLC system (Waters Corp, Milford, MA, USA) was used. Full scan mass spectrometry data in high resolution were acquired using electrospray ionisation positive in a mass range of *m*/*z* 30–1000. Tandem mass spectrometry (MS/MS) were collected on all acquired samples using Bruker broadband collision-induced dissociation (bbCID) function. Resulting data files were processed for peak integration and quantification using the Target Analysis for Screening Quantification (TASQ; v2.2) software (Bruker Daltonics, Bremen, Germany) where calibration curves were linearly fitted with a weighting factor of 1/*x*. For the measurement of tryptophan and associated catabolites, a Waters TQ-XS triple quadrupole (QQQ) coupled to a Waters Acquity I-class UHPLC system (Waters, Wilmslow, UK) was used. The QQQ was operated in positive electrospray ionisation using multiple reaction monitoring (MRM). Raw files were processed for peak integration and metabolite quantification using the the TargetLynx package within MassLynx v4.2 (Waters Corp., Milford, MA) where calibration curves were linearly fitted using a weighting factor of 1/*x*. Resulting data matrices were combined and quality control checked prior to statistical analysis.

### 3.5. LC-MS Lipid Analysis

Serum lipid analysis was performed by ultra-high performance liquid chromatography-tandem mass spectrometry (UHPLC-MS/MS) using predefined MRM transitions Sciex sMRM Pro Builder, Framingham, MA, USA) and in-house chromatographic retention time windows [5]. In brief, serum samples (10 µL) were thawed at 4 °C and prepared for analysis. For quality control (QC), an independent serum pool was prepared as per the samples and aliquots were then injected following each block of nine experimental samples throughout the analytical sequence, which were used for the assessment of analytical precision. Obtained raw files were pre-processed using SkylineMS [103], and quality control random forest signal correction (QC-RFSC) from the statTarget package was used to correct for analytical drift [104]. Feature filtering, RSDQC > 30%, and feature intensity threshold filtering <5000 in >50% of the QCs were applied and metabolites were removed from further statistical analysis based on their failure to meet acceptable analytical precision. The comprehensive analysis covered 865 different lipid species across 19 subclasses (Table S4) of lipids including cholesterol esters (CEs), ceramides (CERs), diacylglycerides (DAGs), dihydroceramides (DCERs), free fatty acids (FFAs), hexosylceramides (HCERs), lactosylceramides (LCERs), lysophosphocholines (LPCs), lysophosphoethanolamines (LPEs), lysophosphoglycerols (LPGs), and lysophosphoinositols (LPIs), monoacylglycerols (MAGs), phosphocholines (PCs), phosphoethanolamines (PEs), phosphoglycerols (PGs), phosphoinositols (PIs), phosphoserines (PSs), sphingomyelins (SMs), and triacylglycerides (TAGs).

### 3.6. Data Analysis

All computation and data visualization was performed using R and RStudio IDE with the open-source R package metabom8 (version 0.2), available from GitHub (github.com/tkimhofer/metabom8 (accessed on 1 June 2022). Orthogonal projection to latent structures-discriminant analysis (O-PLS-DA) [105] was used to model the respiratory symptom variance in the data and to extract discriminating features. An O-PLS-DA model was calculated to differentiate between infected and non-infected samples for the low molecular weight metabolites, lipids, and lipoproteins. In addition, each severity class was modelled against the control group, and the different severity classes were modelled against each other. In order to balance the numbers for severity group B, which contained fewer samples than the other severity groups, only 25 controls were modelled, selected randomly.

The optimal number of orthogonal components for each model was determined using the area under the receiver operator characteristic curve (AUROC) calculated from predictive component scores, generated using an internal sevenfold cross-validation (CV) procedure. The Cliff’s delta statistic was calculated for all the O-PLS-DA models to assess the overall effect size for the intergroup differences [106].

## 4. Conclusions

COVID-19 is a heterogeneous disease with strong patient-to-patient variability of symptoms and severity. We used a multi-platform approach to determine the metabolic signature of SARS-CoV-2 severity across a moderately to severely infected cohort. Whilst stratification of the datasets by metabolite class allowed for deeper insight into the metabolic consequences of SARS-CoV-2 infection, the combined multi-modal dataset delivered a stronger model for predicting infection presence, severity, and ultimate mortality.

Although the number of significant metabolites, lipids, and lipoproteins increased as respiratory severity increased, the core metabolic signature of infection was the same for lipids, lipoproteins, and most low molecular weight metabolites regardless of severity level indicating multiorgan involvement of the disease even in mild cases where no hospitalization was required. This raises the question as to the necessity of long-term monitoring of these patients in relation to PACS to establish their long-term recovery and potentially modified disease risks. Marked alterations on pyruvate, formate, and the lactate to pyruvate ratio indicate perturbation of the tricarboxylic acid cycle and energy metabolism at all levels of infection, whereas the disparity of the Asp:Glu/Asn:Gln indicates liver involvement and the increase in the Apolipoprotein-B100/Apolipoprotein-A1 ratio (ABA1) in combination with changes in other lipid and lipoprotein parameters suggests increased cardiovascular disease risk.

Tryptophan pathway metabolism was heavily disrupted by SARS-CoV-2 infection but in contrast to the majority of metabolites, we find that the disruption of this pathway was associated with infection severity and that the pathway was only substantially disrupted in the hospitalized patients requiring oxygen. The change in balance of the pathway from serotonin to quinolinic acid production indicates a shift towards a neurotoxic systemic environment.

It should be noted that there are limitations within this study. The samples were collected at the start of the pandemic. Several publications have alluded to the altered expression of infection symptoms and generally decreased respiratory severity over the successive waves of SARS-CoV-2 infection, typically corresponding to the progression of variants [107,108], so the infection may have ongoing changing disease risks and potentially different metabolic sequelae. Certain sociodemographic and pre-existing health factors have been shown to be associated with SARS-CoV-2 outcomes such as age, BMI, and chronic health conditions including diabetes and cardiovascular disease. Thus, the distribution of numbers of patients with some of these parameters is skewed for the higher severity categories. Of note, and as expected, greater mortality was observed in the more severe respiratory infection classes (statistics on sociodemographic, anthropometric, and selected clinical parameters are provided in Table S1). Nevertheless, the metabolic signature for mortality was distinct from the signature associated with severity, indicating that the prediction of mortality was not solely related to the severity of respiratory symptoms. As expected, mortality was associated with infection severity and could be predicted based on the hexosylceramide and sphingomyelin profiles 8–61 days prior to death. Early indices of adverse clinical outcomes have value in identifying the most ‘at risk’ patients and may provide a window of opportunity for tailoring the therapeutic monitoring and management of those patients.

## Figures and Tables

**Table 1:** Classification ability and number of significant parameters identified in pairwise comparisons of respiratory severity groups for each molecular panel.

Comparison	Integrated Data Set		Lipids		Lipoproteins		Low mw Metabolites	
	AUROC	No.	AUROC	No.	AUROC	No.	AUROC	No.
Control vs. Group B	0.99	404 (39.07%)	0.98	296 (34.22%)	0.91	65 (58.04%)	0.98	27 (47.37%)
Control vs. Group C	0.99	483 (46.71%)	0.97	372 (43.00%)	0.95	81 (72.32%)	0.99	35 (61.40%)
Control vs. Group D	0.99	537 (51.93%)	0.99	414 (47.86%)	0.97	84 (75.00%)	1.00	39 (68.42%)
Control vs. Group E	1.00	608 (58.80%)	0.99	490 (56.65%)	1.00	83 (74.12%)	1.00	34 (59.65%)
Group B vs. Group C	NS (0.51)	X	NS (0.49)	X	NS (0.58)	X	NS (0.58)	X
Group B vs. Group D	0.62	0 (0.00%)	0.64	0 (0.00%)	0.64	0 (0.00%)	0.76	4 (0.07%)
Group B vs. Group E	0.77	6 (0.01%)	0.79	4 (0.01%)	0.70	0 (0.00%)	0.78	5 (0.09%)
Group C vs. Group D	0.61	12 (0.01%)	NS (0.59)	x	NS (0.58)	x	0.65	15 (26.32%)
Group C vs. Group E	0.73	175 (0.17%)	0.72	81 (0.09%)	0.72	58 (0.52%)	0.74	18 (31.58%)
Group D vs. Group E	0.64	x	NS (0.59)	x	NS (0.59)	x	0.62	1 (0.02%)

NS: not significant.

**Table 2:** Pan Metabolic Severity Mapping of COVID-19 Severity—the top 25 most significant differentiating metabolites in the controls versus each severity group models for the integrated data set, ranked by fold change. The metabolites are colored according to the assay with which the metabolite is measured: lipids (black), lipoproteins (green), and the low molecular weight metabolites (magenta).

Rank	Control vs. B Severity	Control vs. C Severity	Control vs. D Severity	Control vs. E Severity
**1**	**Pyruvic acid**	**Pyruvic acid**	**Pyruvic acid**	**Neopterin/tryptophan**
**2**	**Formic acid**	**Formic acid**	**Formic acid**	**Pyruvic acid**
**3**	**Asp:Glu/Asn:Gln**	**Asp:Glu/Asn:Gln**	**Asp:Glu/Asn:Gln**	**Formic acid**
**4**	**PC (16:0/18:2)**	**H4PL**	**H4A1**	**Asp:Glu/Asn:Gln**
**5**	**PC (18:1/18:2)**	**PC (18:1/18:2)**	**H4PL**	**TPA1**
**6**	**PC (20:0/20:4)**	**H4CH**	**H4A2**	**TPA2**
**7**	**HCER (22:0)**	**HCER.d (18:0/18:0)**	**PC (18:1/18:2)**	**H4A1**
**8**	**HCER.d (18:0/18:0)**	**PE.P (18:0/18:1)**	**H4CH**	**H4PL**
**9**	**PE.P (18:1/20:1)**	**PC (18:2/20:2)**	**PC (18:2/20:2)**	**H4A2**
**10**	**PC (18:2/20:2)**	**Lactate/pyruvate**	**LPC (20:0)**	**PC (18:1/18:2)**
**11**	**PE.P (18:0/20:1)**	**PE.P (18:0/22:5)**	**PE.P (18:0/22:5)**	**H4CH**
**12**	**LPC (18:2)**	**PE.O (16:0/18:1)**	**PE.P (16:0/22:5)**	**PC (18:0/20:1)**
**13**	**PE.P (18:0/20:4)**	**PE.P (16:0/20:1)**	**PE.P (16:0/20:4)**	**PE.P (18:0/22:5)**
**14**	**PE.O (18:0/22:5)**	**PE.P (16:0/22:5)**	**LPC (18:2)**	**PE.P (16:0/22:5)**
**15**	**PE.O (16:0/20:1)**	**LPC (18:2)**	**PE.P (18:1/20:4)**	**PC (20:0/20:3)**
**16**	**PE.P (18:1/18:2)**	**PE.P (18:0/20:4)**	**PE.P (18:1/18:2)**	**PE.P (16:0/20:4)**
**17**	**PC (18:2/18:3)**	**PE.O (16:0/20:1)**	**PC (18:2/18:3)**	**LPC (18:2)**
**18**	**PC (18:2/20:4)**	**PE.P (18:1/18:2)**	**PC (18:2/20:3)**	**PE.P (18:1/20:4)**
**19**	**PE.P (16:0/18:2)**	**PC (18:2/18:3)**	**PE.P (16:0/18:2)**	**PE.P (18:1/18:2)**
**20**	**PE.P (18:2/20:4)**	**PE.P (16:0/18:2)**	**PE.P (18:2/20:4)**	**PC (18:2/20:3)**
**21**	**PC (18:2/18:2)**	**PE.P (18:2/20:4)**	**PC (18:2/18:2**)	**PC (18:2/20:4)**
**22**	**PE.P (18:0/18:2)**	**PC (18:2/18:2)**	**PE.P (18:0/18:2)**	**PE.P (16:0/18:2)**
**23**	**PE.O (16:0/20:4)**	**PE.P (18:0/18:2)**	**PE.O (16:0/20:4)**	**PE.P (18:2/20:4)**
**24**	**PE.P (16:0/18:3)**	**PE.O (16:0/20:4)**	**PE.P (18:2/18:2)**	**PC (18:2/18:2)**
**25**	**PE.O (16:0/18:2)**	**PE.O (16:0/18:2)**	**PE.O (16:0/18:2)**	**PE.O (16:0/20:4**)

## Data Availability

The datasets generated and/or analyzed during the current study are available at the following link: https://zenodo.org/record/7735385 (accessed on 1 July 2023).

## Supplementary Materials

The following supporting information can be downloaded at: https://www.mdpi.com/article/10.3390/ijms241411614/s1.

## References

[1] Nicholson J.K. (2021). Molecular Phenomic Approaches to Deconvolving the Systemic Effects of SARS-CoV-2 Infection and Post-acute COVID-19 Syndrome. Phenomics.

[2] Holmes E., Wist J., Masuda R., Lodge S., Nitschke P., Kimhofer T., Loo R.L., Begum S., Boughton B., Yang R. (2021). Incomplete Systemic Recovery and Metabolic Phenoreversion in Post-Acute-Phase Nonhospitalized COVID-19 Patients: Implications for Assessment of Post-Acute COVID-19 Syndrome. J. Proteome Res..

[3] Kimhofer T., Lodge S., Whiley L., Gray N., Loo R.L., Lawler N.G., Nitschke P., Bong S.-H., Morrison D.L., Begum S. (2020). Integrative Modeling of Quantitative Plasma Lipoprotein, Metabolic, and Amino Acid Data Reveals a Multiorgan Pathological Signature of SARS-CoV-2 Infection. J. Proteome Res..

[4] Lodge S., Nitschke P., Kimhofer T., Coudert J.D., Begum S., Bong S.H., Richards T., Edgar D., Raby E., Spraul M. (2021). NMR Spectroscopic Windows on the Systemic Effects of SARS-CoV-2 Infection on Plasma Lipoproteins and Metabolites in Relation to Circulating Cytokines. J. Proteome Res..

[5] Gray N., Lawler N.G., Zeng A.X., Ryan M., Bong S.H., Boughton B.A., Bizkarguenaga M., Bruzzone C., Embade N., Wist J. (2021). Diagnostic Potential of the Plasma Lipidome in Infectious Disease: Application to Acute SARS-CoV-2 Infection. Metabolites.

[6] Lawler N.G., Gray N., Kimhofer T., Boughton B., Gay M., Yang R., Morillon A.-C., Chin S.-T., Ryan M., Begum S. (2021). Systemic Perturbations in Amine and Kynurenine Metabolism Associated with Acute SARS-CoV-2 Infection and Inflammatory Cytokine Responses. J. Proteome Res..

[7] Lodge S., Nitschke P., Kimhofer T., Wist J., Bong S.-H., Loo R.L., Masuda R., Begum S., Richards T., Lindon J.C. (2021). Diffusion and Relaxation Edited Proton NMR Spectroscopy of Plasma Reveals a High-Fidelity Supramolecular Biomarker Signature of SARS-CoV-2 Infection. Anal. Chem..

[8] Lodge S., Nitschke P., Loo R.L., Kimhofer T., Bong S.-H., Richards T., Begum S., Spraul M., Schaefer H., Lindon J.C. (2021). Low Volume in Vitro Diagnostic Proton NMR Spectroscopy of Human Blood Plasma for Lipoprotein and Metabolite Analysis: Application to SARS-CoV-2 Biomarkers. J. Proteome Res..

[9] Ghini V., Meoni G., Pelagatti L., Celli T., Veneziani F., Petrucci F., Vannucchi V., Bertini L., Luchinat C., Landini G. (2022). Profiling metabolites and lipoproteins in COMETA, an Italian cohort of COVID-19 patients. PLoS Pathog..

[10] Thakur V., Ratho R.K., Kumar P., Bhatia S.K., Bora I., Mohi G.K., Saxena S.K., Devi M., Yadav D., Mehariya S. (2021). Multi-Organ Involvement in COVID-19: Beyond Pulmonary Manifestations. J. Clin. Med..

[11] Lopes-Pacheco M., Silva P.L., Cruz F.F., Battaglini D., Robba C., Pelosi P., Morales M.M., Neves C.C., Rocco P.R.M. (2021). Pathogenesis of Multiple Organ Injury in COVID-19 and Potential Therapeutic Strategies. Front. Physiol..

[12] Veyseh M., Webster P., Blanco I. (2021). COVID-19-associated inflammatory syndrome in an adult woman with unexplained multiple organ failure: Staying vigilant for COVID-19 complications as the pandemic surges. BMJ Case Rep..

[13] Zaim S., Chong J.H., Sankaranarayanan V., Harky A. (2020). COVID-19 and Multiorgan Response. Curr. Probl. Cardiol..

[14] Groff D., Sun A., Ssentongo A.E., Ba D.M., Parsons N., Poudel G.R., Lekoubou A., Oh J.S., Ericson J.E., Ssentongo P. (2021). Short-term and Long-term Rates of Postacute Sequelae of SARS-CoV-2 Infection: A Systematic Review. JAMA Netw. Open.

[15] Bell J., Brown J., Nicholson J., Sadler P. (1987). Assignment of resonances for ‘acute-phase’ glycoproteins in high resolution proton NMR spectra of human blood plasma. FEBS Lett..

[16] Xie Y., Xu E., Bowe B., Al-Aly Z. (2022). Long-term cardiovascular outcomes of COVID-19. Nat. Med..

[17] Nalbandian A., Sehgal K., Gupta A., Madhavan M.V., McGroder C., Stevens J.S., Cook J.R., Nordvig A.S., Shalev D., Sehrawat T.S. (2021). Post-acute COVID-19 syndrome. Nat. Med..

[18] Jiménez B., Holmes E., Heude C., Tolson R.F., Harvey N., Lodge S.L., Chetwynd A.J., Cannet C., Fang F., Pearce J.T.M. (2018). Quantitative Lipoprotein Subclass and Low Molecular Weight Metabolite Analysis in Human Serum and Plasma by ^1^H NMR Spectroscopy in a Multilaboratory Trial. Anal. Chem..

[19] Gray N., Lawler N.G., Yang R., Morillon A.-C., Gay M.C.L., Bong S.-H., Holmes E., Nicholson J.K., Whiley L. (2021). A simultaneous exploratory and quantitative amino acid and biogenic amine metabolic profiling platform for rapid disease phenotyping via UPLC-QToF-MS. Talanta.

[20] Masuda R., Lodge S., Nitschke P., Spraul M., Schaefer H., Bong S.-H., Kimhofer T., Hall D., Loo R.L., Bizkarguenaga M. (2021). Integrative Modeling of Plasma Metabolic and Lipoprotein Biomarkers of SARS-CoV-2 Infection in Spanish and Australian COVID-19 Patient Cohorts. J. Proteome Res..

[21] Bergamaschi L., Mescia F., Turner L., Hanson A.L., Kotagiri P., Dunmore B.J., Ruffieux H., De Sa A., Huhn O., Morgan M.D. (2021). Longitudinal analysis reveals that delayed bystander CD8+ T cell activation and early immune pathology distinguish severe COVID-19 from mild disease. Immunity.

[22] Valdés A., Moreno L.O., Rello S.R., Orduña A., Bernardo D., Cifuentes A. (2022). Metabolomics study of COVID-19 patients in four different clinical stages. Sci. Rep..

[23] Rothan H.A., Byrareddy S.N. (2020). The epidemiology and pathogenesis of coronavirus disease (COVID-19) outbreak. J. Autoimmun..

[24] Coperchini F., Chiovato L., Croce L., Magri F., Rotondi M. (2020). The cytokine storm in COVID-19: An overview of the involvement of the chemokine/chemokine-receptor system. Cytokine Growth Factor. Rev..

[25] Leisman D.E., Ronner L., Pinotti R., Taylor M.D., Sinha P., Calfee C.S., Hirayama A.V., Mastroiani F., Turtle C.J., Harhay M.O. (2020). Cytokine elevation in severe and critical COVID-19: A rapid systematic review, meta-analysis, and comparison with other inflammatory syndromes. Lancet Respir. Med..

[26] Chau A.S., Weber A.G., Maria N.I., Narain S., Liu A., Hajizadeh N., Malhotra P., Bloom O., Marder G., Kaplan B. (2021). The Longitudinal Immune Response to Coronavirus Disease 2019: Chasing the Cytokine Storm. Arthritis Rheumatol..

[27] Collier M.E., Zhang S., Scrutton N.S., Giorgini F. (2021). Inflammation control and improvement of cognitive function in COVID-19 infections: Is there a role for kynurenine 3-monooxygenase inhibition?. Drug Discov. Today.

[28] Costanzo M., Caterino M., Fedele R., Cevenini A., Pontillo M., Barra L., Ruoppolo M. (2022). COVIDomics: The Proteomic and Metabolomic Signatures of COVID-19. Int. J. Mol. Sci..

[29] López-Hernández Y., Monárrez-Espino J., Oostdam A.-S.H.-V., Delgado J.E.C., Zhang L., Zheng J., Valdez J.J.O., Mandal R., González F.d.L.O., Moreno J.C.B. (2021). Targeted metabolomics identifies high performing diagnostic and prognostic biomarkers for COVID-19. Sci. Rep..

[30] Yan H., Liang X., Du J., He Z., Wang Y., Lyu M., Yue L., Zhang F., Xue Z., Xu L. (2021). Proteomic and metabolomic investigation of serum lactate dehydrogenase elevation in COVID-19 patients. Proteomics.

[31] Walldius G., Frank S., Kostner G. (2012). The apoB/apoA-I Ratio is a Strong Predictor of Cardiovascular Risk. Lipoproteins.

[32] Rafsanjani M.R., Pramana T.Y. (2021). Arifin Correlation of Fischer’s Ratio with Liver Fibrosis in Naive Chronic Hepatitis B Patients without Comorbidities. Asian J. Pharmacy Nurs. Med. Sci..

[33] Cihan M., Doğan Ö., Serdar C.C., Yıldırım A.A., Kurt C., Serdar M.A. (2022). Kynurenine pathway in Coronavirus disease (COVID-19): Potential role in prognosis. J. Clin. Lab. Anal..

[34] Marín-Corral J., Rodríguez-Morató J., Gomez-Gomez A., Pascual-Guardia S., Muñoz-Bermúdez R., Salazar-Degracia A., Pérez-Terán P., Restrepo M.I., Khymenets O., Haro N. (2021). Metabolic Signatures Associated with Severity in Hospitalized COVID-19 Patients. Int. J. Mol. Sci..

[35] Bruzzone C., Bizkarguenaga M., Gil-Redondo R., Diercks T., Arana E., de Vicuña A.G., Seco M., Bosch A., Palazón A., Juan I.S. (2020). SARS-CoV-2 Infection Dysregulates the Metabolomic and Lipidomic Profiles of Serum. Iscience.

[36] Codo A.C., Davanzo G.G., de Brito Monteiro L., de Souza G.F., Muraro S.P., Virgilio-da-Silva J.V., Prodonoff J.S., Carregari V.C., de Biagi Junior C.A.O., Crunfli F. (2020). Elevated Glucose Levels Favor SARS-CoV-2 Infection and Monocyte Response through a HIF-1α/Glycolysis-Dependent Axis. Cell Metab..

[37] McElvaney O.J., McEvoy N.L., McElvaney O.F., Carroll T.P., Murphy M.P., Dunlea D.M., Choileáin O.N., Clarke J., Eoin O’Connor H.G. (2020). Characterization of the Inflammatory Response to Severe COVID-19 Illness. Am. J. Respir. Crit. Care Med..

[38] Ruffieux H., Hanson A.L., Lodge S., Lawler N.G., Whiley L., Gray N., Nolan T.H., Bergamaschi L., Mescia F., Turner L. (2023). A patient-centric characterization of systemic recovery from SARS-CoV-2 infection. Nat. Immunol..

[39] Zhou F., Yu T., Du R., Fan G., Liu Y., Liu Z., Xiang J., Wang Y., Song B., Gu X. (2020). Clinical course and risk factors for mortality of adult inpatients with COVID-19 in Wuhan, China: A retrospective cohort study. Lancet.

[40] Grant R.S., Passey R., Matanovic G., Smythe G., Kapoor V. (1999). Evidence for increased de novo synthesis of NAD in im-mune-activated RAW264.7 macrophages: A self-protective mechanism?. Arch. Biochem. Biophys..

[41] Páez-Franco J.C., Torres-Ruiz J., Sosa-Hernández V.A., Cervantes-Díaz R., Romero-Ramírez S., Pérez-Fragoso A., Meza-Sánchez D.E., Germán-Acacio J.M., Maravillas-Montero J.L., Mejía-Domínguez N.R. (2021). Metabolomics analysis reveals a modified amino acid metabolism that correlates with altered oxygen homeostasis in COVID-19 patients. Sci. Rep..

[42] Li X., Tu B., Zhang X., Xu W., Chen J., Zhao G., Xu B., Zheng J., Yan Y., Hao P. (2022). Dysregulation of glutamine/glutamate metabolism in COVID-19 patients: A metabolism study in African population and mini meta-analysis. J. Med. Virol..

[43] Cengiz M., Uysal B.B., Ikitimur H., Ozcan E., Islamoğlu M.S., Aktepe E., Yavuzer H., Yavuzer S. (2020). Effect of oral l-Glutamine supplementation on Covid-19 treatment. Clin. Nutr. Exp..

[44] Masoodi M., Peschka M., Schmiedel S., Haddad M., Frye M., Maas C., Lohse A., Huber S., Kirchhof P., Nofer J.-R. (2022). Disturbed lipid and amino acid metabolisms in COVID-19 patients. J. Mol. Med..

[45] Matsuyama T., Yoshinaga S.K., Shibue K., Mak T.W. (2021). Comorbidity-associated glutamine deficiency is a predisposition to severe COVID-19. Cell Death Differ..

[46] Krishnan S., Nordqvist H., Ambikan A.T., Gupta S., Sperk M., Svensson-Akusjärvi S., Mikaeloff F., Benfeitas R., Saccon E., Ponnan S.M. (2021). Metabolic Perturbation Associated With COVID-19 Disease Severity and SARS-CoV-2 Replication. Mol. Cell. Proteom..

[47] Rubino F., Amiel S.A., Zimmet P., Alberti G., Bornstein S., Eckel R.H., Mingrone G., Boehm B., Cooper M.E., Chai Z. (2020). New-Onset Diabetes in Covid-19. N. Engl. J. Med..

[48] Ssentongo P., Zhang Y., Witmer L., Chinchilli V.M., Ba D.M. (2022). Association of COVID-19 with diabetes: A systematic review and meta-analysis. Sci. Rep..

[49] Liu X., Zheng Y., Guasch-Ferré M., Ruiz-Canela M., Toledo E., Clish C., Liang L., Razquin C., Corella D., Estruch R. (2019). High plasma glutamate and low glutamine-to-glutamate ratio are associated with type 2 diabetes: Case-cohort study within the PREDIMED trial. Nutr. Metab. Cardiovasc. Dis..

[50] Samocha-Bonet D., Wong O., Synnott E.-L., Piyaratna N., Douglas A., Gribble F.M., Holst J.J., Chisholm D.J., Greenfield J.R. (2011). Glutamine Reduces Postprandial Glycemia and Augments the Glucagon-Like Peptide-1 Response in Type 2 Diabetes Patients. J. Nutr..

[51] Albrecht P., Lewerenz J., Dittmer S., Noack R., Maher P., Methner A. (2010). Mechanisms of Oxidative Glutamate Toxicity: The Glutamate/Cystine Antiporter System xc¯ as a Neuroprotective Drug Target. CNS Neurol. Disord. Drug Targets.

[52] Correia B.S.B., Ferreira V.G., Piagge P.M.F.D., Almeida M.B., Assunção N.A., Raimundo J.R.S., Fonseca F.L.A., Carrilho E., Cardoso D.R. (2022). ^1^H qNMR-Based Metabolomics Discrimination of Covid-19 Severity. J. Proteome Res..

[53] Schmelter F., Föh B., Mallagaray A., Rahmöller J., Ehlers M., Lehrian S., von Kopylow V., Künsting I., Lixenfeld A.S., Martin E. (2021). Metabolic and Lipidomic Markers Differentiate COVID-19 From Non-Hospitalized and Other Intensive Care Patients. Front. Mol. Biosci..

[54] Guo L., Schurink B., Roos E., Nossent E.J., Duitman J.W., Vlaar A.P., van der Valk P., Vaz F.M., Yeh S.-R., Geeraerts Z. (2022). Indoleamine 2,3-dioxygenase (IDO)-1 and IDO-2 activity and severe course of COVID-19. J. Pathol..

[55] Kluck G.E.G., Yoo J.-A., Sakarya E.H., Trigatti B.L. (2021). Good Cholesterol Gone Bad? HDL and COVID-19. Int. J. Mol. Sci..

[56] Wei C., Wan L., Yan Q., Wang X., Zhang J., Yang X., Zhang Y., Fan C., Li D., Deng Y. (2020). HDL-scavenger receptor B type 1 facilitates SARS-CoV-2 entry. Nat. Metab..

[57] Feingold K.R., Feingold K.R., Anawalt B., Blackman M.R., Boyce A., Chrousos G., Corpas E., de Herder W.W., Dhatariya K., Dungan K., Hofland J. (2022). Lipid and Lipoprotein Levels in Patients with COVID-19 Infections. Endotext.

[58] Harbaum L., Ghataorhe P., Wharton J., Jiménez B., Howard L.S.G., Gibbs J.S.R., Nicholson J.K., Rhodes C.J., Wilkins M.R. (2019). Reduced plasma levels of small HDL particles transporting fibrinolytic proteins in pulmonary arterial hy-pertension. Thorax.

[59] Long A.T., Kenne E., Jung R., Fuchs T.A., Renné T. (2016). Contact system revisited: An interface between inflammation, coagulation, and innate immunity. J. Thromb. Haemost..

[60] Walldius G., Jungner I. (2007). Apolipoprotein A-I versus HDL cholesterol in the prediction of risk for myocardial infarction and stroke. Curr. Opin. Cardiol..

[61] Avogaro P., Bon G.B., Cazzolato G., Quinci G.B. (1979). Are apolipoproteins better discriminators than lipids for atherosclerosis?. Lancet.

[62] Walldius G., Jungner I., Holme I., Aastveit A.H., Kolar W., Steiner E. (2001). High apolipoprotein B, low apolipoprotein A-I, and improvement in the prediction of fatal myocardial in-farction (AMORIS study): A prospective study. Lancet.

[63] Masuda R., Lodge S., Whiley L., Gray N., Lawler N., Nitschke P., Bong S.-H., Kimhofer T., Loo R.L., Boughton B. (2022). Exploration of Human Serum Lipoprotein Supramolecular Phospholipids Using Statistical Heterospectroscopy in n-Dimensions (SHY-n): Identification of Potential Cardiovascular Risk Biomarkers Related to SARS-CoV-2 Infection. Anal. Chem..

[64] Wu D., Shu T., Yang X., Song J.-X., Zhang M., Yao C., Liu W., Huang M., Yu Y., Yang Q. (2020). Plasma metabolomic and lipidomic alterations associated with COVID-19. Natl. Sci. Rev..

[65] Almulla A.F., Supasitthumrong T., Tunvirachaisakul C., Algon A.A.A., Al-Hakeim H.K., Maes M. (2022). The tryptophan catabolite or kynurenine pathway in COVID-19 and critical COVID-19: A systematic review and meta-analysis. BMC Infect. Dis..

[66] Gostner J.M., Geisler S., Stonig M., Mair L., Sperner-Unterweger B., Fuchs D. (2020). Tryptophan Metabolism and Related Pathways in Psychoneuroimmunology: The Impact of Nutrition and Lifestyle. Neuropsychobiology.

[67] Stone T.W. (2001). Endogenous neurotoxins from tryptophan. Toxicon.

[68] Zádori D., Klivényi P., Vámos E., Fülöp F., Toldi J., Vécsei L. (2009). Kynurenines in chronic neurodegenerative disorders: Future therapeutic strategies. J. Neural Transm..

[69] Kincses Z.T., Toldi J., Vécsei L. (2010). Kynurenines, neurodegeneration and Alzheimer’s disease. J. Cell. Mol. Med..

[70] Vyavahare S., Kumar S., Cantu N., Kolhe R., Bollag W.B., McGee-Lawrence M.E., Hill W.D., Hamrick M.W., Isales C.M., Fulzele S. (2021). Tryptophan-Kynurenine Pathway in COVID-19-Dependent Musculoskeletal Pathology: A Minireview. Mediat. Inflamm..

[71] Chuang S.-C., Fanidi A., Ueland P.M., Relton C., Midttun O., Vollset S.E., Gunter M.J., Seckl M.J., Travis R.C., Wareham N. (2014). Circulating Biomarkers of Tryptophan and the Kynurenine Pathway and Lung Cancer Risk. Cancer Epidemiol. Biomark. Prev..

[72] Grant R., Coggan S., Smythe G. (2009). The Physiological Action of Picolinic Acid in the Human Brain. Int. J. Tryptophan Res..

[73] Medana I.M., Day N.P.J., Salahifar-Sabet H., Stocker R., Smythe G., Bwanaisa L., Njobvu A., Kayira K., Turner G.D.H., Taylor T.E. (2003). Metabolites of the Kynurenine Pathway of Tryptophan Metabolism in the Cerebrospinal Fluid of Malawian Children with Malaria. J. Infect. Dis..

[74] Danlos F.-X., Grajeda-Iglesias C., Durand S., Sauvat A., Roumier M., Cantin D., Colomba E., Rohmer J., Pommeret F., Baciarello G. (2021). Metabolomic analyses of COVID-19 patients unravel stage-dependent and prognostic biomarkers. Cell Death Dis..

[75] Clayton T.A., Lindon J.C., Cloarec O., Antti H., Charuel C., Hanton G., Provost J.-P., Le Net J.-L., Baker D., Walley R.J. (2006). Pharmaco-metabonomic phenotyping and personalized drug treatment. Nature.

[76] Clayton T.A., Baker D., Lindon J.C., Everett J.R., Nicholson J.K. (2009). Pharmacometabonomic identification of a significant host-microbiome metabolic interaction affecting human drug metabolism. Proc. Natl. Acad. Sci. USA.

[77] McPhail M.J., Shawcross D.L., Lewis M.R., Coltart I., Want E.J., Antoniades C.G., Veselkov K., Triantafyllou E., Patel V., Pop O. (2016). Multivariate metabotyping of plasma predicts survival in patients with decompensated cirrhosis. J. Hepatol..

[78] Cas M.D., Ottolenghi S., Morano C., Rinaldo R., Roda G., Chiumello D., Centanni S., Samaja M., Paroni R. (2021). Link between serum lipid signature and prognostic factors in COVID-19 patients. Sci. Rep..

[79] Abusukhun M., Winkler M.S., Pöhlmann S., Moerer O., Meissner K., Tampe B., Hofmann-Winkler H., Bauer M., Gräler M.H., Claus R.A. (2021). Activation of Sphingomyelinase-Ceramide-Pathway in COVID-19 Purposes Its Inhibition for Therapeutic Strategies. Front. Immunol..

[80] Laaksonen R., Ekroos K., Sysi-Aho M., Hilvo M., Vihervaara T., Kauhanen D., Suoniemi M., Hurme R., März W., Scharnagl H. (2016). Plasma ceramides predict cardiovascular death in patients with stable coronary artery disease and acute coronary syndromes beyond LDL-cholesterol. Eur. Heart J..

[81] Leimanis-Laurens M., Wolfrum E., Ferguson K., Grunwell J.R., Sanfilippo D., Prokop J.W., Lydic T.A., Rajasekaran S. (2021). Hexosylceramides and Glycerophosphatidylcholine GPC(36:1) Increase in Multi-Organ Dysfunction Syndrome Patients with Pediatric Intensive Care Unit Admission over 8-Day Hospitalization. J. Pers. Med..

[82] Zhang J.-Y., Qu F., Li J.-F., Liu M., Ren F., Zhang J.-Y., Bian D.-D., Chen Y., Duan Z.-P., Zhang J.-L. (2016). Up-regulation of Plasma Hexosylceramide (d18: 1/18: 1) Contributes to Genotype 2 Virus Replication in Chronic Hepatitis C: A 20-Year Cohort Study. Medicine.

[83] Vitner E.B., Avraham R., Politi B., Melamed S., Israely T. (2022). Elevation in sphingolipid upon SARS-CoV-2 infection: Possible implications for COVID-19 pathology. Life Sci. Alliance.

[84] Smadja D.M., Philippe A., Bory O., Gendron N., Beauvais A., Gruest M., Peron N., Khider L., Guerin C.L., Goudot G. (2021). Placental growth factor level in plasma predicts COVID-19 severity and in-hospital mortality. J. Thromb. Haemost..

[85] Villoteau A., Asfar M., Otekpo M., Loison J., Gautier J., Annweiler C., on behalf of the GERIA-COVID study group (2021). Elevated C-reactive protein in early COVID-19 predicts worse survival among hospitalized geriatric patients. PLoS ONE.

[86] De Lorenzo R., Sciorati C., Ramirez G.A., Colombo B., Lorè N.I., Capobianco A., Tresoldi C., Cirillo D.M., Ciceri F., Corti A. (2022). Chromogranin A plasma levels predict mortality in COVID-19. PLoS ONE.

[87] Li Y., Deng Y., Ye L., Sun H., Du S., Huang H., Zeng F., Chen X., Deng G. (2021). Clinical Significance of Plasma D-Dimer in COVID-19 Mortality. Front. Med..

[88] Ahirwar A.K., Takhelmayum R., Sakarde A., Rathod B.D., Jha P.K., Kumawat R., Gopal N. (2022). The study of serum hsCRP, ferritin, IL-6 and plasma D-dimer in COVID-19: A retrospective study. Horm. Mol. Biol. Clin. Investig..

[89] D’amora P., Silva I.D.C.G., Budib M.A., Ayache R., Silva R.M.S., Silva F.C., Appel R.M., Júnior S.S., Pontes H.B.D., Alvarenga A.C. (2021). Towards risk stratification and prediction of disease severity and mortality in COVID-19: Next generation metabolomics for the measurement of host response to COVID-19 infection. PLoS ONE.

[90] Masvekar R.R., Kosa P., Jin K., Dobbs K., Stack M.A., Castagnoli R., Quaresima V., Su H.C., Imberti L., Notarangelo L.D. (2022). Prognostic value of serum/plasma neurofilament light chain for COVID -19-associated mortality. Ann. Clin. Transl. Neurol..

[91] Masana L., Correig E., Ibarretxe D., Anoro E., Arroyo J.A., Jericó C., Guerrero C., Miret M., Näf S., Pardo A. (2021). Low HDL and high triglycerides predict COVID-19 severity. Sci. Rep..

[92] Richard V.R., Gaither C., Popp R., Chaplygina D., Brzhozovskiy A., Kononikhin A., Mohammed Y., Zahedi R.P., Nikolaev E.N., Borchers C.H. (2022). Early Prediction of COVID-19 Patient Survival by Targeted Plasma Multi-Omics and Machine Learning. Mol. Cell. Proteom..

[93] Lu C., Wang Y., Sheng Z., Liu G., Fu Z., Zhao J., Zhao J., Yan X., Zhu B., Peng S. (2010). NMR-based metabonomic analysis of the hepatotoxicity induced by combined exposure to PCBs and TCDD in rats. Toxicol. Appl. Pharmacol..

[94] Spriet L.L., Whitfield J. (2015). Taurine and skeletal muscle function. Curr. Opin. Clin. Nutr. Metab. Care.

[95] Gonzalez A., Orozco-Aguilar J., Achiardi O., Simon F., Cabello-Verrugio C. (2020). SARS-CoV-2/Renin-Angiotensin System: De-ciphering the Clues for a Couple with Potentially Harmful Effects on Skeletal Muscle. Int. J. Mol. Sci..

[96] Ottenhoff M.C., Ramos L.A., Potters W., Janssen M.L.F., Hubers D., Hu S., Fridgeirsson E.A., Piña-Fuentes D., Thomas R., van der Horst I.C.C. (2021). Predicting mortality of individual patients with COVID-19: A multicentre Dutch cohort. BMJ Open.

[97] Izcovich A., Ragusa M.A., Tortosa F., Marzio M.A.L., Agnoletti C., Bengolea A., Ceirano A., Espinosa F., Saavedra E., Sanguine V. (2020). Prognostic factors for severity and mortality in patients infected with COVID-19: A systematic review. PLoS ONE.

[98] Hasan M.R., Suleiman M., Pérez-López A. (2021). Metabolomics in the Diagnosis and Prognosis of COVID-19. Front. Genet..

[99] Loo R.L., Lodge S., Kimhofer T., Bong S.-H., Begum S., Whiley L., Gray N., Lindon J.C., Nitschke P., Lawler N.G. (2020). Quantitative In-Vitro Diagnostic NMR Spectroscopy for Lipoprotein and Metabolite Measurements in Plasma and Serum: Recommendations for Analytical Artifact Minimization with Special Reference to COVID-19/SARS-CoV-2 Samples. J. Proteome Res..

[100] Dona A.C., Jiménez B., Schäfer H., Humpfer E., Spraul M., Lewis M.R., Pearce J.T.M., Holmes E., Lindon J.C., Nicholson J.K. (2014). Precision High-Throughput Proton NMR Spectroscopy of Human Urine, Serum, and Plasma for Large-Scale Metabolic Phenotyping. Anal. Chem..

[101] Whiley L., Nye L.C., Grant I., Andreas N., Chappell K.E., Sarafian M.H., Misra R., Plumb R.S., Lewis M.R., Nicholson J.K. (2019). Ultrahigh-Performance Liquid Chromatography Tandem Mass Spectrometry with Electrospray Ionization Quantification of Tryptophan Metabolites and Markers of Gut Health in Serum and Plasma-Application to Clinical and Epidemiology Cohorts. Anal. Chem..

[102] Gray N., Zia R., King A., Patel V.C., Wendon J., McPhail M.J.W., Coen M., Plumb R.S., Wilson I.D., Nicholson J.K. (2017). High-Speed Quantitative UPLC-MS Analysis of Multiple Amines in Human Plasma and Serum via Precolumn Derivatization with 6-Aminoquinolyl-*N*-hydroxysuccinimidyl Carbamate: Application to Acetaminophen-Induced Liver Failure. Anal. Chem..

[103] Adams K.J., Pratt B., Bose N., Dubois L.G., John-Williams L.S., Perrott K.M., Ky K., Kapahi P., Sharma V., MacCoss M.J. (2020). Skyline for Small Molecules: A Unifying Software Package for Quantitative Metabolomics. J. Proteome Res..

[104] Luan H., Ji F., Chen Y., Cai Z. (2018). statTarget: A streamlined tool for signal drift correction and interpretations of quantitative mass spectrometry-based omics data. Anal. Chim. Acta.

[105] Bylesjö M., Rantalainen M., Cloarec O., Nicholson J.K., Holmes E., Trygg J. (2006). OPLS discriminant analysis: Combining the strengths of PLS-DA and SIMCA classification. J. Chemom..

[106] Cliff N. (1993). Dominance statistics: Ordinal analyses to answer ordinal questions. Psychol. Bull..

[107] Otshudiema J.O., Folefack G.L.T., Nsio J.M., Mbala-Kingebeni P., Kakema C.H., Kosianza J.B., Mfumu A.K., Saidi G.N., Kabongo P.M., Okum R. (2022). Epidemiological Comparison of Four COVID-19 Waves in the Democratic Republic of the Congo, March 2020–January 2022. J. Epidemiol. Glob. Health.

[108] Abdullah F., Myers J., Basu D., Tintinger G., Ueckermann V., Mathebula M., Ramlall R., Spoor S., de Villiers T., Van der Walt Z. (2022). Decreased severity of disease during the first global omicron variant covid-19 outbreak in a large hospital in tshwane, south africa. Int. J. Infect. Dis..

