# Supplementary material for: Integrative Plasma Metabolic and Lipidomic Modelling of SARS-CoV-2 Infection in Relation to Clinical Severity and Early Mortality Prediction
Source: Int J Mol Sci. 2023 Jul 18;24(14):11614. doi: 10.3390/ijms241411614 (PMC10380980; doi:10.3390/ijms241411614)
Supplement: Supplementary file 1 [file ijms-24-11614-s001.zip › ijms-2461623-supplementary.pdf]

Integrative Plasma Metabolic and Lipidomic Modelling of SARS-CoV-2 Infection in Relation to Clinical Severity and Early Mortality Prediction

Samantha Lodge [1,2], Nathan Lawler [1,2], Nicola Gray [1,2], Reika Masuda [1], Philipp Nitschke [1], Luke Whiley [1,2], Sze-How Bong [1], Bu B. Yeap [3,4], G. Dwivedi [3,5], Manfred Spraul [6], Hartmut Schaefer [6], Rubén Gil-Redondo [7], Nieves Embade [7], Oscar Millet [7], Elaine Holmes [1,2,8], Julien Wist [1,2,9*], and Jeremy K. Nicholson [1,2,10*]

[1]*Australian National Phenome Center, Health Futures Institute, Murdoch University, Harry Perkins Building, Perth, WA 6150, Australia*

[2]*Center for Computational and Systems Medicine, Health Futures Institute, Murdoch University, Harry Perkins Building, Perth, WA 6150, Australia*

[3]*Medical School, University of Western Australia, Perth, WA 6150, Australia*

[4]*Department of Endocrinology and Diabetes, Fiona Stanley Hospital, Perth, WA 6150, Australia*

[5]*Department of Cardiology, Fiona Stanley Hospital, Perth, WA 6150, Australia*

[6]*Bruker Biospin GmbH, 76287 Rheinstetten, Germany*

[7]*Precision Medicine and Metabolism Laboratory, CIC bioGUNE, Parque Tecnológico de Bizkaia, Bld. 800, 48160, Derio, Spain*

[8]*Department of Metabolism, Digestion and Reproduction, Faculty of Medicine, Imperial College London, Sir Alexander Fleming Building, South Kensington, London, SW7 2AZ, UK*

[9]*Chemistry Department, Universidad del Valle, Cali 76001, Colombia*

[10]*Institute of Global Health Innovation, Faculty of Medicine, Imperial College London, Level 1, Faculty Building, South Kensington Campus, London, SW7 2NA, UK*

Key words: COVID-19, SARS-CoV-2, NMR spectroscopy, mass spectrometry, plasma IVDr, metabolic phenotyping, diagnostic modeling, lipoproteins, lipids, mortality prediction, patient stratification.

Acknowledgements: We thank the support provided by the Department of Industry, Tourism and Trade of the Government of the Autonomous Community of the Basque Country (Elkartek BG2019) and the Agencia Estatal de Investigación (Spain) for grant RTI2018-101269-B-I00. We thank The Spinnaker Health Research Foundation, WA, The McCusker Foundation, WA, The Western Australian State Government, and the MRFF (grant number 2014349) for funding the Australian National Phenome Centre for this and related work. We thank the Department of Jobs, Tourism, Science and Innovation, Government of Western Australian Premier's Fellowship for funding EH; and ARC Laureate Fellowship funding for EH. We also would like to acknowledge the Western Australian Covid Research Response team (https://research-au.net/covid-research-response/). JW thanks Ministerio de Ciencia, Tecnología e Innovación (Minciencias), Ministerio de Educación Nacional, Ministerio de Industria, Comercio y Turismo e ICETEX (792–2017) 2a Convocatoria Ecosistema Científico - Colombia Científica para la Financiación de Proyectos de I + D + i), World Bank and Vicerrectoría de Investigaciones, Pontificia Universidad Javeriana, Bogotá, Colombia (contract no. FP44842 - 221-2018).

Table of contents

**Section 1 – Cohort Demographic Data**	**Page**
Table S1 - Full cohort demographic data for the control samples and SARS-CoV-2 positive patients.	S6
**Section 2 - Quantification of Plasma Lipoproteins**	
Table S2 - Annotation of the keys used by the Bruker IVDr Lipoprotein Subclass Analysis (B.I.-LISA™) method.	S7
Table S3 - Annotation of the low molecular weight metabolites	S9
Table S4 - Annotation of the lipids	S11
**Section 3 - Integrated Spectroscopy Modeling**	
Table S5 - OPLS loadings, Cliff's delta and adjusted p-values of the combined model (low molecular weight metabolites, lipoproteins and lipids) for the comparison of the controls and SARS-CoV-2 positive patients.	S11
Figure S1 - Radar plot showing the $\log_2$ fold change of the top 50 metabolites, lipoproteins and lipids with the most significant p-values for the control versus SARS-CoV-2 positive patients.	S28
Figure S2 - Orthogonal Partial Least Squared (O-PLS-DA) and eruption plots between controls and SARS-CoV-2 positive patients of the integrative pan-metabolic model after correction for age	S29
Figure S3 - O-PLS-DA scores plots of the low molecular weight metabolites, lipoproteins and lipids.	S30
Figure S4 – Orthogonal Partial Least Squared (O-PLS-DA) and eruption plots between controls and SARS-CoV-2 positive patients of each severity group for the four Integrated assays.	S31
Table S6 – OPLS loadings, Cliff's delta and adjusted p-values of all the assays combined for the comparison of the controls and SARS-CoV-2 positive patients in severity class B.	S32
Table S7 – OPLS loadings, Cliff's delta and adjusted p-values of all the assays combined for the comparison of the controls and SARS-CoV-2 positive patients in severity class C.	S33
Table S8 – OPLS loadings, Cliff's delta and adjusted p-values of all the assays combined for the comparison of the controls and SARS-CoV-2 positive patients in severity class D.	S35
Table S9 – OPLS loadings, Cliff's delta and adjusted p-values of all the assays combined for the comparison of the controls and SARS-CoV-2 positive patients in severity class E.	S36
Figure S5 – Concentrations of metabolites for the controls and each severity class.	S38
**Section 4 - Severity group modeling**	
Figure S6 – Orthogonal Partial Least Squared (O-PLS-DA) and eruption plots between controls and SARS-CoV-2 positive patients of each severity group for the lipoproteins.	S39

Figure S7 – −log 10 (p-values) between controls and SARS-CoV-2 positive patients of each severity group for the lipoproteins.	S40
Table S10 – OPLS loadings, Cliff's delta and adjusted p-values of the lipoproteins for the comparison of the controls and SARS-CoV-2 positive patients in severity class B.	S41
Table S11 – OPLS loadings, Cliff's delta and adjusted p-values of the lipoproteins for the comparison of the controls and SARS-CoV-2 positive patients in severity class C.	S43
Table S12 – OPLS loadings, Cliff's delta and adjusted p-values of the lipoproteins for the comparison of the controls and SARS-CoV-2 positive patients in severity class D.	S45
Table S13 – OPLS loadings, Cliff's delta and adjusted p-values of the lipoproteins for the comparison of the controls and SARS-CoV-2 positive patients in severity class E.	S47
Figure S8 – Orthogonal Partial Least Squared (O-PLS-DA) and eruption plots between different severity classes of SARS-CoV-2 positive patients for the lipoproteins.	S50
Table S14 – OPLS loadings, Cliff's delta and adjusted p-values of the lipoproteins for the comparison of severity class C and E.	S51
Figure S9 – Orthogonal Partial Least Squared (O-PLS-DA) and eruption plots between controls and SARS-CoV-2 positive patients of each severity group for the Lipids.	S53

Table S15 – OPLS loadings, Cliff's delta and adjusted p-values of the lipids for the comparison of the controls and SARS-CoV-2 positive patients in severity class B.	S54
Table S16 – OPLS loadings, Cliff's delta and adjusted p-values of the lipids for the comparison of the controls and SARS-CoV-2 positive patients in severity class C.	S55
Table S17 – OPLS loadings, Cliff's delta and adjusted p-values of the lipids for the comparison of the controls and SARS-CoV-2 positive patients in severity class D.	S57
Table S18 – OPLS loadings, Cliff's delta and adjusted p-values of the lipids for the comparison of the controls and SARS-CoV-2 positive patients in severity class E.	S58
Figure S10 – Orthogonal Partial Least Squared (O-PLS-DA) and eruption plots between different severity classes of SARS-CoV-2 positive patients for the lipids	S60
Table S19 – OPLS loadings, Cliff's delta and adjusted p-values of the lipids for the comparison of severity class B and E.	S61
Table S20 – OPLS loadings, Cliff's delta and adjusted p-values of the lipids for the comparison of severity class C and E.	S18
Figure S11 – Orthogonal Partial Least Squared (O-PLS-DA), eruption plots and −log 10 (p-values) between controls and SARS-CoV-2 positive patients of each severity group for the low molecular weight metabolites.	S64
Table S21 – OPLS loadings, Cliff's delta and adjusted p-values of the low molecular weight metabolites for the comparison of the controls and SARS-CoV-2 positive patients in severity class B.	S65
Table S22 – OPLS loadings, Cliff's delta and adjusted p-values of the low molecular weight metabolites for the comparison of the controls and SARS-CoV-2 positive patients in severity class C.	S66
Table S23 – OPLS loadings, Cliff's delta and adjusted p-values of the low molecular weight metabolites for the comparison of the controls and SARS-CoV-2 positive patients in severity class D.	S67
Table S24 – OPLS loadings, Cliff's delta and adjusted p-values of the low molecular weight metabolites for the comparison of the controls and SARS-CoV-2 positive patients in severity class E.	S68
Figure S12 – Orthogonal Partial Least Squared (O-PLS-DA), eruption plots and −log 10 (p-values) between different severity classes of SARS-CoV-2 positive patients for the low molecular weight metabolites.	S70
Table S25 – OPLS loadings, Cliff's delta and adjusted p-values for the low molecular weight metabolites for the comparison of severity class B and D.	S71
Table S26 – OPLS loadings, Cliff's delta and adjusted p-values for the low molecular weight metabolites for the comparison of severity class B and E.	S71
Table S27 – OPLS loadings, Cliff's delta and adjusted p-values for the low molecular weight metabolites for the comparison of severity class C and D.	S71
Table S28 – OPLS loadings, Cliff's delta and adjusted p-values for the low molecular weight metabolites for the comparison of severity class C and E.	S72
Table S29 – OPLS loadings, Cliff's delta and adjusted p-values for the low molecular weight metabolites for the comparison of severity class D and E.	S73
Figure S13 – Radar plots showing the $\log_2$ fold change between controls and SARS-CoV-2 severity group E patients of the most significant ($p<0.05$) metabolites, lipids and lipoproteins ordered by $\log_2$ fold change.	S74
**Section 6 - Mortality prediction**	

Table S30 – Demographics for the severity group E patients who survived and who did not.	S75
Table S31 - OPLS loadings, Cliff's delta and adjusted p-values of the combined model (low molecular weight metabolites, lipoproteins and lipids) for the comparison group E severity patients comparing who survived and who died, samples were age matched.	S76
Figure S14 – Correlation plot of the (A) survived model and (B) Did not survive model.	S88
Figure S15 – Modeling COVID-19 mortality prediction using only the lipoproteins	S89
Table S32 – OPLS loadings, Cliff's delta and adjusted p-values of lipoprotein model for the comparison group E severity patients comparing who survived and who died, samples were age matched.	S90
Figure S16 - AUROC built using HCer 16:0 and PEO (18:0/18:1)	S91

Table S1 - Full cohort demographic data for the control samples and SARS-CoV-2 positive patients.

	Controls (n=89)	SARS-CoV-2 Positive (+) Group B severity (n=18)	SARS-CoV-2 Positive (+) Group C severity (n=139)	SARS-CoV-2 Positive (+) Group D severity (n=92)	SARS-CoV-2 Positive (+) Group E severity (n=57)
Sex, Male	43 (48.31%)	5 (27.78%)	56 (40.29%)	44 (47.83%)	33 (57.89%)
Age, years [SD]	48.00 [±11.95]	62.00 [±18.13]	68.00 [±18.21]	71.00 [±17.22]	75.00 [±13.46]
BMI, kg/m^2 [SD]	23.81 [±4.57]	-	-	-	-
Pre-diabetic/ diabetic	-	3 (16.67%)	29 (20.86%)	24 (26.09%)	19 (33.33%)
Hypertension	-	5 (27.78%)	48 (34.53%)	50 (54.35%)	33 (57.89%)
Cardiovascular disease	-	3 (16.67%)	33 (23.74%)	29 (31.52%)	21 (36.84%)
Cerebrovascular disease	-	2 (11.11%)	16 (11.51%)	9 (9.78%)	10 (17.54%)
Chronic Renal Disease	-	0	8 (5.76%)	9 (9.78%)	6 (10.53%)
Chronic liver disease	-	0	2 (1.44%)	1 (1.09%)	2 (3.51%)
Patients who died of SARS-CoV-2	-	0	0	0	32 (56.14%)

Quantification of Plasma Lipoproteins, Lipids and the low molecular weight metabolites

Plasma lipoproteins – A total of 112 lipoprotein parameters were quantified based on 1D NMR experiments as part of Bruker's IVDr experiment suite for blood plasma. This approach is termed Bruker's IVDr lipoprotein class and subclass Analysis (B.I.-LISA™) and lipid analytes include cholesterol, free cholesterol, phospholipids, triglycerides, apolipoproteins A1/A2/B100 and ratio B100/A1, in total plasma concentration and resolved for main lipoprotein classes and subclasses. Main classes of plasma-lipoproteins were defined as: high-density lipoprotein (HDL, density 1.063–1.210 kg/L), intermediate-density lipoprotein (IDL, density 1.006–1.019 kg/L) low-density lipoprotein (LDL, density 1.09–1.63 kg/L) and very low-density lipoprotein (VLDL, 0.950–1.006 kg/L). The main lipoprotein classes HDL, LDL, VLDL were further divided into different lipoprotein sub-classes: (LDL-1: 1.019–1.031 kg/L, LDL-2: 1.031–1.034 kg/L, LDL-3: 1.034–1.037 kg/L, LDL-4: 1.037–1.040 kg/L, LDL-5: 1.040–1.044 kg/L, LDL-6: 1.044–1.063 kg/L), and

the HDL sub-fractions into four density classes (HDL-1 1.063–1.100 kg/L, HDL-2 1.100–1.125 kg/L, HDL-3 1.125–1.175 kg/L, and HDL-4 1.175–1.210 kg/L), the VLDL sub-fractions divided into 5 density classes.

Table S2- Annotation of the keys used by the Bruker IVDr Lipoprotein Subclass Analysis (B.I.-LISA ™) method. Abbreviations: LDL – low-density lipoprotein; HDL – high-density lipoprotein; VLDL – very low-density lipoprotein; IDL – intermediate-density lipoprotein.

Key	Class/subclass	Compound	Concentration unit
TPTG	Total Plasma	Triglycerides	mg/dL
TPCH	Total Plasma	Cholesterol	mg/dL
LDCH	LDL	Cholesterol	mg/dL
HDCH	HDL	Cholesterol	mg/dL
TPA1	Total Plasma	Apolipoprotein-A1	mg/dL
TPA2	Total Plasma	Apolipoprotein-A2	mg/dL
TPAB	Total Plasma	Apolipoprotein-B100	mg/dL
LDHD	Ratio LDL and HDL Cholesterol	LDL Cholesterol / HDL Cholesterol	-/-
ABA1	Ratio of Apolipoproteins A1 and B100	Apolipoprotein-A1 / Apolipoprotein-B100	-/-
TBPN	Apolipoprotein-B100 carrying particles	Particle Number	nmol/L
VLPN	VLDL	Particle Number	nmol/L
IDPN	IDL	Particle Number	nmol/L
LDPN	LDL	Particle Number	nmol/L
L1PN	LDL-1	Particle Number	nmol/L
L2PN	LDL-2	Particle Number	nmol/L
L3PN	LDL-3	Particle Number	nmol/L
L4PN	LDL-4	Particle Number	nmol/L
L5PN	LDL-5	Particle Number	nmol/L
L6PN	LDL-6	Particle Number	nmol/L
VLTG	VLDL Class	Triglycerides	mg/dL
IDTG	IDL Class	Triglycerides	mg/dL
LDTG	LDL Class	Triglycerides	mg/dL
HDTG	HDL Class	Triglycerides	mg/dL
VLCH	VLDL Class	Cholesterol	mg/dL
IDCH	IDL Class	Cholesterol	mg/dL
LDCH	LDL Class	Cholesterol	mg/dL
HDCH	HDL Class	Cholesterol	mg/dL
VLFC	VLDL Class	Free Cholesterol	mg/dL
IDFC	IDL Class	Free Cholesterol	mg/dL
LDFC	LDL Class	Free Cholesterol	mg/dL
HDFC	HDL Class	Free Cholesterol	mg/dL
VLPL	VLDL Class	Phospholipids	mg/dL
IDPL	IDL Class	Phospholipids	mg/dL
LDPL	LDL Class	Phospholipids	mg/dL
HDPL	HDL Class	Phospholipids	mg/dL

HDA1	HDL Class	Apolipoprotein-A1	mg/dL
HDA2	HDL Class	Apolipoprotein-A2	mg/dL
VLAB	VLDL Class	Apolipoprotein-B100	mg/dL
IDAB	IDL Class	Apolipoprotein-B100	mg/dL
LDAB	LDL Class	Apolipoprotein-B100	mg/dL
V1TG	VLDL-1 Subclass	Triglycerides	mg/dL
V2TG	VLDL-2 Subclass	Triglycerides	mg/dL
V3TG	VLDL-3 Subclass	Triglycerides	mg/dL
V4TG	VLDL-4 Subclass	Triglycerides	mg/dL
V5TG	VLDL-5 Subclass	Triglycerides	mg/dL
V1CH	VLDL-1 Subclass	Cholesterol	mg/dL
V2CH	VLDL-2 Subclass	Cholesterol	mg/dL
V3CH	VLDL-3 Subclass	Cholesterol	mg/dL
V4CH	VLDL-4 Subclass	Cholesterol	mg/dL
V5CH	VLDL-5 Subclass	Cholesterol	mg/dL
V1FC	VLDL-1 Subclass	Free Cholesterol	mg/dL
V2FC	VLDL-2 Subclass	Free Cholesterol	mg/dL
V3FC	VLDL-3 Subclass	Free Cholesterol	mg/dL
V4FC	VLDL-4 Subclass	Free Cholesterol	mg/dL
V5FC	VLDL-5 Subclass	Free Cholesterol	mg/dL
V1PL	VLDL-1 Subclass	Phospholipids	mg/dL
V2PL	VLDL-2 Subclass	Phospholipids	mg/dL
V3PL	VLDL-3 Subclass	Phospholipids	mg/dL
V4PL	VLDL-4 Subclass	Phospholipids	mg/dL
V5PL	VLDL-5 Subclass	Phospholipids	mg/dL
L1TG	LDL-1 Subclass	Triglycerides	mg/dL
L2TG	LDL-2 Subclass	Triglycerides	mg/dL
L3TG	LDL-3 Subclass	Triglycerides	mg/dL
L4TG	LDL-4 Subclass	Triglycerides	mg/dL
L5TG	LDL-5 Subclass	Triglycerides	mg/dL
L6TG	LDL-6 Subclass	Triglycerides	mg/dL
L1CH	LDL-1 Subclass	Cholesterol	mg/dL
L2CH	LDL-2 Subclass	Cholesterol	mg/dL
L3CH	LDL-3 Subclass	Cholesterol	mg/dL
L4CH	LDL-4 Subclass	Cholesterol	mg/dL
L5CH	LDL-5 Subclass	Cholesterol	mg/dL
L6CH	LDL-6 Subclass	Cholesterol	mg/dL
L1FC	LDL-1 Subclass	Free apoHDA Cholesterol	mg/dL
L2FC	LDL-2 Subclass	Free Cholesterol	mg/dL
L3FC	LDL-3 Subclass	Free Cholesterol	mg/dL
L4FC	LDL-4 Subclass	Free Cholesterol	mg/dL
L5FC	LDL-5 Subclass	Free Cholesterol	mg/dL
L6FC	LDL-6 Subclass	Free Cholesterol	mg/dL
L1PL	LDL-1 Subclass	Phospholipids	mg/dL
L2PL	LDL-2 Subclass	Phospholipids	mg/dL

L3PL	LDL-3 Subclass	Phospholipids	mg/dL
L4PL	LDL-4 Subclass	Phospholipids	mg/dL
L5PL	LDL-5 Subclass	Phospholipids	mg/dL
L6PL	LDL-6 Subclass	Phospholipids	mg/dL
L1AB	LDL-1 Subclass	Apolipoprotein-B100	mg/dL
L2AB	LDL-2 Subclass	Apolipoprotein-B100	mg/dL
L3AB	LDL-3 Subclass	Apolipoprotein-B100	mg/dL
L4AB	LDL-4 Subclass	Apolipoprotein-B100	mg/dL
L5AB	LDL-5 Subclass	Apolipoprotein-B100	mg/dL
L6AB	LDL-6 Subclass	Apolipoprotein-B100	mg/dL
H1TG	HDL-1 Subclass	Triglycerides	mg/dL
H2TG	HDL-2 Subclass	Triglycerides	mg/dL
H3TG	HDL-3 Subclass	Triglycerides	mg/dL
H4TG	HDL-4 Subclass	Triglycerides	mg/dL
H1CH	HDL-1 Subclass	Cholesterol	mg/dL
H2CH	HDL-2 Subclass	Cholesterol	mg/dL
H3CH	HDL-3 Subclass	Cholesterol	mg/dL
H4CH	HDL-4 Subclass	Cholesterol	mg/dL
H1FC	HDL-1 Subclass	Free Cholesterol	mg/dL
H2FC	HDL-2 Subclass	Free Cholesterol	mg/dL
H3FC	HDL-3 Subclass	Free Cholesterol	mg/dL
H4FC	HDL-4 Subclass	Free Cholesterol	mg/dL
H1PL	HDL-1 Subclass	Phospholipids	mg/dL
H2PL	HDL-2 Subclass	Phospholipids	mg/dL
H3PL	HDL-3 Subclass	Phospholipids	mg/dL
H4PL	HDL-4 Subclass	Phospholipids	mg/dL
H1A1	HDL-1 Subclass	Apolipoprotein-A1	mg/dL
H2A1	HDL-2 Subclass	Apolipoprotein-A1	mg/dL
H3A1	HDL-3 Subclass	Apolipoprotein-A1	mg/dL
H4A1	HDL-4 Subclass	Apolipoprotein-A1	mg/dL
H1A2	HDL-1 Subclass	Apolipoprotein-A2	mg/dL
H2A2	HDL-2 Subclass	Apolipoprotein-A2	mg/dL
H3A2	HDL-3 Subclass	Apolipoprotein-A2	mg/dL
H4A2	HDL-4 Subclass	Apolipoprotein-A2	mg/dL

Table S3 - Annotation of the low molecular weight metabolites. Those in italics were quantified by NMR, while the remaining were quantified by LC-MS.

Abbreviations in models	Metabolite	Concentration unit
Acetic acid	*Acetic acid*	*mmol/L*
Acetoacetic acid	*Acetoacetic acid*	*mmol/L*
Acetone	*Acetone*	*mmol/L*
Citric acid	*Citric acid*	*mmol/L*
Creatine	*Creatine*	*mmol/L*
Creatinine	*Creatinine*	*mmol/L*
Formic acid	*Formic acid*	*mmol/L*

Glucose	*Glucose*	*mmol/L*
D-3-hydroxybutryic acid	*D-3-hydroxybutryic acid*	*mmol/L*
Lactic acid	*Lactic acid*	*mmol/L*
Pyruvic acid	*Pyruvic acid*	*mmol/L*
Tryptophan	Tryptophan	µmol/L
3-Hydroxykynurenine	3-Hydroxykynurenine	µmol/L
3-Hydroxyanthranilic acid	3-Hydroxyanthranilic acid	µmol/L
Kynurenic acid	Kynurenic acid	µmol/L
Quinolinic acid	Quinolinic acid	µmol/L
Picolinic acid	Picolinic acid	µmol/L
Xanthurenic acid	Xanthurenic acid	µmol/L
Kynurenine	Kynurenine	µmol/L
Indole-3-acetic acid	Indole-3-acetic acid	µmol/L
5-Hydroxyindole acetic acid	5-Hydroxyindole acetic acid	µmol/L
Neopterin	Neopterin	µmol/L
Serotonin	Serotonin	µmol/L
1-methylhistidine	1-methylhistidine	µmol/L
3-methylhistidine	3-methylhistidine	µmol/L
Alanine	Alanine	µmol/L
Alpha aminobutyric acid	Alpha aminobutyric acid	µmol/L
Arginine	Arginine	µmol/L
Aspartic acid	Aspartic acid	µmol/L
Citrulline	Citrulline	µmol/L
Glutamic acid	Glutamic acid	µmol/L
Glutamine	Glutamine	µmol/L
Histidine	Histidine	µmol/L
Isoleucine	Isoleucine	µmol/L
Leucine	Leucine	µmol/L
Lysine	Lysine	µmol/L
Methionine	Methionine	µmol/L
Ornithine	Ornithine	µmol/L
Phenylalanine	Phenylalanine	µmol/L
Proline	Proline	µmol/L
Serine	Serine	µmol/L
Taurine	Taurine	µmol/L
Threonine	Threonine	µmol/L
Tyrosine	Tyrosine	µmol/L
Valine	Valine	µmol/L
Lac.pyr	*Lactate/pyruvate*	*-/-*
Kynurenine/tryptophan	Kynurenine/tryptophan	-/-
3-hydroxykynurenine/tryptophan	3-hydroxykynurenine/tryptophan	-/-
Kynurenic acid/tryptophan	Kynurenic acid/tryptophan	-/-
Neopterin/tryptophan	Neopterin/tryptophan	-/-
Serotonin/tryptophan	Serotonin/tryptophan	-/-
Quinolinic acid/tryptophan	Quinolinic acid/tryptophan	-/-
Fisher’s ratio	Fisher’s ratio	-/-

Branched AA/taurine	Branched chain amino acids/aromatic amino acids	-/-
Asp…glu…asn…gln	Asp:Glu/Asn:Gln	-/-

Table S4 - Annotation of the lipids

Abbreviation	Subclasses	Concentration unit
CE	Cholesterol esters	μmol/L
CER	Ceramides	μmol/L
DAG	Diacylglycerides	μmol/L
DCER	Dihydroceramides	μmol/L
FFA	Free fatty acids	μmol/L
HCER	Hexosylceramides	μmol/L
LCER	Lactosylceramides	μmol/L
LPC	Lysophosphocholines	μmol/L
LPE	Lysophosphoethanolamines	μmol/L
LPG	Lysophosphoglycerols	μmol/L
LPI	Lysophosphoinositols	μmol/L
MAG	Monoacylglycerols	μmol/L
PC	Phosphocholines	μmol/L
PE	Phosphoethanolamines	μmol/L
PG	Phosphoglycerols	μmol/L
PI	Phosphoinositols	μmol/L
PS	Phosphoserines	μmol/L
SM	Sphinogomyelins	μmol/L
TAG	Triacylglycerides	μmol/L

Integrated Spectroscopy Modeling

Table S5 - OPLS loadings, Cliff's delta and adjusted p-values of the combined model (low molecular weight metabolites, lipoproteins and lipids) for the comparison of the controls and SARS-CoV-2 positive patients. Only metabolites with significant p-values are shown in the table.

Metabolite	OPLS loadings	Cliff's delta	Adjusted p value
Formic acid	0.063	1.00	$1.18x10^{-43}$
PC (18:2/18:2)	0.085	-0.94	$4.48x10^{-39}$
PE.O (16:0/20:4)	0.073	-0.94	$4.48x10^{-39}$
Asp:Glu/Asn:Gln	0.039	0.93	$1.21x10^{-38}$
Pyruvic acid	0.050	0.93	$2.67x10^{-38}$
PE.P (18:1/20:4)	0.076	-0.93	$2.67x10^{-38}$
PC (18:1/18:2)	0.085	-0.92	$5.41x10^{-38}$
H4CH	0.069	-0.92	$8.87x10^{-38}$
H4PL	0.071	-0.92	$1.08x10^{-37}$

PE.P (18:1/18:2)	0.082	-0.92	$1.21x10^{-37}$
PE.P (16:0/18:2)	0.082	-0.91	$2.00x10^{-37}$
PE.P (16:0/22:5)	0.073	-0.91	$2.00x10^{-37}$
PE.O (16:0/18:2)	0.074	-0.91	$2.60x10^{-37}$
PE.P (18:1/20:4)	0.077	-0.91	$2.60x10^{-37}$
LPC (18:2)	0.077	-0.91	$8.25x10^{-37}$
PE.P (18:0/18:2)	0.079	-0.90	$8.25x10^{-37}$
PE.P (18:0/22:5)	0.076	-0.90	$2.27x10^{-36}$
PE.P (16:0/20:4)	0.074	-0.90	$3.76x10^{-36}$
PC (18:2/20:3)	0.077	-0.90	$3.86x10^{-36}$
PC (18:2/20:2)	0.076	-0.89	$5.52x10^{-36}$
PE.P (18:2/18:2)	0.076	-0.88	$4.01x10^{-35}$
Lactate/pyruvate	0.046	-0.88	$6.10x10^{-35}$
PC (20:0/20:3)	0.075	-0.88	$6.10x10^{-35}$
PE.P (18:0/18:1)	0.077	-0.88	$7.95x10^{-35}$
HCER.d (18:0/18:0)	0.074	-0.88	$8.72x10^{-35}$
PE.O (16:0/20:1)	0.073	-0.88	$9.46x10^{-35}$
H4A1	0.067	-0.87	$2.68x10^{-34}$
PE.P (18:1/22:5)	0.074	-0.87	$2.95x10^{-34}$
PE.O (16:0/22:5)	0.072	-0.87	$3.00x10^{-34}$
PE.P (18:0/20:4)	0.073	-0.87	$3.40x10^{-34}$
PE.P (16:0/20:1)	0.077	-0.87	$4.35x10^{-34}$
PC (18:2/20:4)	0.079	-0.87	$4.81x10^{-34}$
H4A2	0.067	-0.86	$6.21x10^{-34}$
PE.P (16:0/18:3)	0.066	-0.86	$6.21x10^{-34}$
PC (18:0/20:1)	0.076	-0.86	$6.96x10^{-34}$
PE.O (18:0/22:5)	0.070	-0.86	$6.96x10^{-34}$
LPC (20:0)	0.075	-0.86	$1.03x10^{-33}$
PE.P (18:0/22:4)	0.069	-0.86	$1.22x10^{-33}$
PE.O (16:0/18:1)	0.067	-0.86	$1.70x10^{-33}$
PE.P (18:0/20:1)	0.074	-0.85	$4.02x10^{-33}$
PE.P (18:0/20:3)	0.073	-0.85	$4.59x10^{-33}$
PC (18:2/18:3)	0.063	-0.84	$2.40x10^{-32}$
PE.P (18:0/16:0)	0.075	-0.84	$2.71x10^{-32}$
PE.P (18:1/22:4)	0.062	-0.84	$4.30x10^{-32}$
PE.P (18:0/20:2)	0.074	-0.84	$5.79x10^{-32}$
PE.P (18:1/18:1)	0.063	-0.84	$6.28x10^{-32}$

HCER (22:0)	0.069	-0.83	$8.56x10^{-32}$
Glutamic acid	0.045	0.83	$1.59x10^{-31}$
HCER (26:0)	0.072	-0.83	$1.59x10^{-31}$
Glutamic acid	0.046	0.83	$1.69x10^{-31}$
HCER.d (18:0/22:0)	0.069	-0.83	$2.01x10^{-31}$
PE.P (18:0/18:0)	0.071	-0.83	$3.79x10^{-31}$
LDTG	0.048	0.83	$4.06X10^{-31}$
PC (20:0/18:1)	0.068	-0.82	$4.58x10^{-31}$
PI (18:1/18:1)	0.068	-0.82	$7.43x10^{-31}$
PI (20:0/18:2)	0.070	-0.82	$1.04x10^{-30}$
PE.O (18:0/22:4)	0.052	-0.81	$2.46x10^{-30}$
PC (18:2/22:6)	0.074	-0.81	$3.22x10^{-30}$
HCER.d (18:0/24:0)	0.069	-0.81	$3.25x10^{-30}$
SM (20:0)	0.064	-0.81	$4.74x10^{-30}$
HCER (24:0)	0.069	-0.81	$5.28x10^{-30}$
PE.P (16:0/22:4)	0.057	-0.81	$5.45x10^{-30}$
TPA2	0.065	-0.81	$7.21x10^{-30}$
CER (26:0)	0.072	-0.81	$7.64x10^{-30}$
PC (18:2/22:5)	0.072	-0.80	$1.55x10^{-29}$
LPI (20:3)	0.062	-0.80	$1.73x10^{-29}$
PE.P (18:1/20:1)	0.070	-0.80	$3.81x10^{-29}$
PC (14:0/18:2)	0.071	-0.79	$7.22x10^{-29}$
SM (24:0)	0.065	-0.79	$8.74x10^{-29}$
PE.P (18:1/16:0)	0.034	-0.79	$1.98x10^{-28}$
LPC (20:3)	0.069	-0.79	$2.02x10^{-28}$
PC (16:1/18:2)	0.068	-0.78	$3.14x10^{-28}$
PC (16:0/18:2)	0.071	-0.78	$4.28x10^{-28}$
PI (18:1/20:4)	0.069	-0.78	$6.18x10^{-28}$
PI (18:1/18:2)	0.067	-0.78	$6.55x10^{-28}$
H4FC	0.062	-0.78	$6.60x10^{-28}$
MAG (20:3)	0.015	0.78	$7.44x10^{-28}$
LPI (18:1)	0.058	-0.78	$8.17x10^{-28}$
PC (18:2/20:5)	0.057	-0.78	$8.17x10^{-28}$
LPC (18:1)	0.069	-0.77	$3.10x10^{-27}$
SM (26:0)	0.064	-0.77	$3.17x10^{-27}$
Aspartic acid	0.046	0.77	$4.05x10^{-27}$
DCER (24:0)	0.072	-0.77	$4.09x10^{-27}$

PI (18:0/20:3)	0.069	-0.77	$4.09x10^{-27}$
HDA2	0.063	-0.77	$4.99x10^{-27}$
PE.P (16:0/20:5)	0.060	-0.76	$7.34x10^{-27}$
PE.P (16:0/18:1)	0.063	-0.76	$8.05x10^{-27}$
HDA1	0.066	-0.76	$1.29x10^{-26}$
PC (18:1/20:3)	0.070	-0.75	$3.20x10^{-26}$
PE.O (16:0/22:4)	0.056	-0.75	$3.59x10^{-26}$
PC (14:0/20:4)	0.066	-0.75	$4.26x10^{-26}$
HCER (20:0)	0.059	-0.75	$4.62x10^{-26}$
PC (14:0/18:3)	0.050	-0.75	$8.38x10^{-26}$
LPC (18:3)	0.060	-0.75	$8.78x10^{-26}$
Neopterin/tryptophan	0.024	0.71	$1.49x10^{-25}$
MAG (20:4)	0.015	0.74	$1.52x10^{-25}$
TPA1	0.066	-0.74	$1.68x10^{-25}$
PC (18:2/16:1)	0.065	-0.74	$1.68x10^{-25}$
PC (16:0/20:1)	0.068	-0.74	$1.69x10^{-25}$
Fisher’s ratio	0.046	-0.74	$2.07x10^{-25}$
PI (20:0/20:4)	0.065	-0.74	$2.27x10^{-25}$
PC (20:0/20:4)	0.067	-0.74	$2.38x10^{-25}$
PC (18:0/20:2)	0.069	-0.74	$4.68x10^{-25}$
PI (18:1/20:3)	0.063	-0.73	$5.49x10^{-25}$
LPI (18:2)	0.049	-0.73	$5.56x10^{-25}$
PC (14:0/22:6)	0.065	-0.73	$1.10x10^{-24}$
PC (18:0/18:2)	0.068	-0.73	$1.44x10^{-24}$
H3FC	0.062	-0.72	$2.53x10^{-24}$
Quinolinic acid/tryptophan	0.016	0.72	$3.12x10^{-24}$
PE.P (18:1/20:3)	0.062	-0.72	$3.79x10^{-24}$
PE.P (18:1/20:5)	0.055	-0.72	$6.03x10^{-24}$
PS (20:0/18:2)	0.065	-0.72	$6.67x10^{-24}$
LPC (20:2)	0.067	-0.72	$7.30x10^{-24}$
LPI (20:4)	0.048	-0.72	$8.16x10^{-24}$
PG (20:0/18:2)	0.056	-0.72	$8.18x10^{-24}$
PE.O (16:0/22:6)	0.055	-0.71	$8.75x10^{-24}$
CER (24:0)	0.066	-0.71	$9.16x10^{-24}$
DCER (26:0)	0.062	-0.71	$1.48x10^{-23}$
PI (18:2/18:2)	0.051	-0.71	$1.49x10^{-23}$
HCER (14:0)	0.057	-0.71	$1.61x10^{-23}$

HDCH	0.061	-0.71	2.04×10^{-23}
PC (18:1/20:1)	0.061	-0.71	2.39×10^{-23}
PS (18:0/18:0)	0.041	0.71	2.40×10^{-23}
PC (14:0/20:3)	0.062	-0.71	2.71×10^{-23}
LPE (18:2)	0.052	-0.70	4.36×10^{-23}
Phenylalanine	0.046	0.70	6.63×10^{-23}
H2TG	0.038	0.70	6.63×10^{-23}
PC (18:1/18:3)	0.049	-0.70	8.92×10^{-23}
CE (16:0)	0.060	-0.70	9.44×10^{-23}
H3CH	0.061	-0.70	1.18×10^{-22}
L4TG	0.042	0.70	1.27×10^{-22}
PE.P (16:0/20:3)	0.059	-0.69	1.75×10^{-22}
PE.P (16:0/16:1)	0.054	-0.69	1.99×10^{-22}
PE.P (18:1/20:2)	0.063	-0.69	2.55×10^{-22}
MAG (18:1)	0.015	0.69	2.81×10^{-22}
PC (14:0/18:1)	0.058	-0.68	6.84×10^{-22}
SM (14:0)	0.063	-0.68	7.59×10^{-22}
PC (20:0/22:5)	0.057	-0.68	2.20×10^{-21}
DCER (26:1)	0.060	-0.67	2.36×10^{-21}
HCER.d (18:0/20:0)	0.055	-0.67	2.36×10^{-21}
PI (18:0/20:4)	0.061	-0.67	2.36×10^{-21}
PC (14:0/20:5)	0.049	-0.67	3.20×10^{-21}
Histidine	0.046	-0.67	3.34×10^{-21}
CE (18:1)	0.057	-0.67	4.03×10^{-21}
PC (18:0/20:3)	0.063	-0.67	4.09×10^{-21}
PE.P (18:1/18:0)	0.055	-0.67	5.09×10^{-21}
LCER (24:0)	0.054	-0.67	5.37×10^{-21}
L1TG	0.037	0.67	5.50×10^{-21}
L5TG	0.041	0.67	5.69×10^{-21}
Quinolinic acid	0.020	0.67	6.90×10^{-21}
PC (18:1/22:4)	0.056	-0.66	1.00×10^{-20}
CE (18:3)	0.050	-0.66	1.11×10^{-20}
PS (18:0/20:4)	0.047	-0.66	1.29×10^{-20}
Neopterin	0.026	0.66	1.39×10^{-20}
CER (18:0)	0.036	0.66	2.41×10^{-20}
PC (14:0/14:0)	0.040	-0.66	2.90×10^{-20}
CE (14:0)	0.053	-0.65	3.66×10^{-20}

Citrulline	0.042	-0.65	$4.91x10^{-20}$
L2TG	0.041	0.65	$5.56x10^{-20}$
PC (14:0/22:5)	0.053	-0.65	$5.69x10^{-20}$
PC (16:0/18:0)	0.019	-0.65	$6.19x10^{-20}$
HDPL	0.058	-0.65	$7.11x10^{-20}$
LPE (20:3)	0.042	-0.65	$9.47x10^{-20}$
PE (18:2/16:1)	0.055	-0.65	$1.24x10^{-19}$
LPC (20:4)	0.057	-0.64	$2.25x10^{-19}$
LPC (22:5)	0.055	-0.64	$3.79x10^{-19}$
PC (16:0/20:3)	0.063	-0.64	$4.08x10^{-19}$
H3PL	0.055	-0.64	$4.49x10^{-19}$
H3A1	0.058	-0.63	$7.57x10^{-19}$
PG (20:0/20:3)	0.038	-0.63	$8.70x10^{-19}$
PE.P (18:0/20:5)	0.051	-0.63	$1.12x10^{-19}$
LCER.d (18:0/22:0)	0.052	-0.63	$1.44x10^{-18}$
PC (18:0/18:1)	0.049	-0.63	$1.44x10^{-18}$
CE (22:0)	0.053	-0.63	$1.44x10^{-18}$
PC (18:0/18:0)	0.050	-0.63	$1.51x10^{-18}$
PS (20:0/18:3)	0.049	-0.63	$1.51x10^{-18}$
LPC (18:0)	0.062	-0.62	$1.87x10^{-18}$
CE (24:0)	0.047	-0.62	$2.09x10^{-18}$
LPE (20:4)	0.045	-0.62	$3.38x10^{-18}$
LCER.d (18:0/24:0)	0.051	-0.62	$3.52x10^{-18}$
PS (20:0/20:4)	0.057	-0.62	$4.75x10^{-18}$
DCER (22:0)	0.052	-0.62	$5.19x10^{-18}$
PC (14:0/22:4)	0.049	-0.61	$6.72x10^{-18}$
PS (20:0/20:5)	0.047	-0.61	$6.94x10^{-18}$
PE.O (18:0/18:2)	0.039	-0.61	$1.07x10^{-17}$
VLPN	0.032	0.61	$1.35x10^{-17}$
VLAB	0.032	0.61	$1.35x10^{-17}$
ABA1	0.040	0.61	$1.51x10^{-17}$
LPC (22:4)	0.052	-0.61	$1.57x10^{-17}$
PG (18:0/18:2)	0.049	-0.60	$2.60x10^{-17}$
PC (18:1/22:6)	0.057	-0.60	$2.78x10^{-17}$
PC (18:0/22:4)	0.052	-0.60	$3.27x10^{-17}$
DCER (22:1)	0.050	-0.60	$3.91x10^{-17}$
V4TG	0.033	0.60	$4.83x10^{-17}$

PI (16:0/20:3)	0.053	-0.60	$5.15x10^{-17}$
PI (18:0/20:2)	0.042	-0.59	$6.92x10^{-17}$
LPE (18:1)	0.051	-0.59	$7.48x10^{-17}$
PE (18:2/20:4)	0.054	-0.59	$1.03x10^{-16}$
PE (18:2/18:2)	0.053	-0.59	$1.19x10^{-16}$
Glutamine	0.037	-0.59	$1.27x10^{-16}$
LPC (22:6)	0.050	-0.59	$1.40x10^{-16}$
PG (18:0/18:1)	0.051	-0.59	$1.71x10^{-16}$
SM (20:1)	0.050	-0.59	$1.91x10^{-16}$
PC (16:0/20:4)	0.053	-0.59	$1.96x10^{-16}$
PC (14:0/20:2)	0.051	-0.59	$2.07x10^{-16}$
PC (18:1/18:1)	0.050	-0.58	$2.26x10^{-16}$
LPE (18:3)	0.028	-0.58	$2.52x10^{-16}$
PI (18:0/18:1)	0.050	-0.58	$3.01x10^{-16}$
CE (18:0)	0.052	-0.58	$4.67x10^{-16}$
SM (24:1)	0.051	-0.58	$5.75x10^{-16}$
HCER.d (18:0/26:0)	0.045	-0.58	$5.78x10^{-16}$
PG (18:1/16:1)	0.045	0.58	$6.63x10^{-16}$
MAG (22:5)	0.013	0.58	$6.77x10^{-16}$
V3TG	0.027	0.57	$1.40x10^{-15}$
CE (20:2)	0.053	-0.57	$1.51x10^{-15}$
PE.P (16:0/22:6)	0.050	-0.57	$1.51x10^{-15}$
LPE (20:1)	0.049	-0.57	$1.91x10^{-15}$
PI (16:0/20:4)	0.047	-0.56	$4.21x10^{-15}$
PC (16:0/22:4)	-0.050	-0.56	$4.85x10^{-15}$
V2FC	0.025	0.56	$5.15x10^{-15}$
H1TG	0.027	0.55	$7.66x10^{-15}$
PI (18:0/22:4)	0.049	-0.55	$1.04x10^{-14}$
PG (20:0/20:5)	0.035	-0.55	$1.11x10^{-14}$
LPE (20:2)	0.041	-0.55	$1.15x10^{-14}$
LCER (14:0)	0.042	-0.55	$1.66x10^{-14}$
CE (20:3)	0.053	-0.54	$2.30x10^{-14}$
L6TG	0.035	0.54	$2.54x10^{-14}$
HDFC	0.050	-0.54	$2.94x10^{-14}$
LPC (20:5)	0.040	-0.54	$3.71x10^{-14}$
Kynurenine/tryptophan	0.033	0.54	$3.84x10^{-14}$
PS (20:0/22:6)	0.051	-0.54	$4.10x10^{-14}$

SM (22:1)	0.044	-0.54	$4.70x10^{-14}$
PG (18:0/20:0)	0.036	0.54	$4.80x10^{-14}$
PC (18:2/20:1)	0.042	-0.53	$1.08x10^{-13}$
PE.O (18:0/20:4)	0.040	-0.53	$1.13x10^{-13}$
PC (16:0/20:5)	0.040	-0.53	$1.18x10^{-13}$
V3FC	0.024	0.53	$1.42x10^{-13}$
MAG (22:4)	0.014	0.53	$1.51x10^{-13}$
PI (20:0/16:1)	0.050	-0.53	$1.52x10^{-13}$
PC (16:0/14:0)	0.040	-0.53	$1.57x10^{-13}$
CE (20:1)	0.037	-0.52	$2.48x10^{-13}$
MAG (16:0)	0.012	0.52	$2.88x10^{-13}$
PI (16:0/18:1)	0.045	-0.52	$3.00x10^{-13}$
PC (16:1/18:1)	0.036	-0.52	$3.29x10^{-13}$
MAG (18:2)	0.013	0.52	$5.34x10^{-13}$
H2A1	0.048	-0.51	$6.06x10^{-13}$
LCER (16:0)	0.038	-0.51	$8.86x10^{-13}$
PG (18:1/18:2)	0.035	0.51	$9.70x10^{-13}$
PC (18:0/14:0)	0.044	-0.51	$1.05x10^{-12}$
PE.P (16:0/18:0)	0.044	-0.51	$1.22x10^{-12}$
Threonine	0.027	-0.51	$1.24x10^{-12}$
3-Hydroxykynurenine	0.022	0.47	$1.42x10^{-12}$
H3TG	0.024	0.51	$1.53x10^{-12}$
PG (20:0/20:1)	0.047	-0.51	$1.58x10^{-12}$
PC (16:0/22:6)	0.049	-0.51	$1.61x10^{-12}$
LPC (14:0)	0.046	-0.50	$1.87x10^{-12}$
PI (16:0/20:2)	0.047	-0.50	$1.95x10^{-12}$
Lactic acid	0.030	0.50	$2.42x10^{-12}$
PE (18:1/18:2)	0.036	-0.50	$2.38x10^{-12}$
HCER.d (18:0/26:1)	0.032	-0.50	$2.45x10^{-12}$
V4PL	0.031	0.50	$2.96x10^{-12}$
HCER (22:1)	-0.037	-0.50	$2.95x10^{-12}$
LPC (20:1)	0.051	-0.50	$3.23x10^{-12}$
PC (16:0/18:3)	0.026	-0.50	$3.96x10^{-12}$
PC (16:0/18:1)	0.040	-0.50	$4.32x10^{-12}$
H3A2	0.046	-0.49	$6.54x10^{-12}$
PI (18:0/20:0)	0.039	0.49	$6.54x10^{-12}$
PC (18:1/20:4)	0.046	-0.49	$7.01x10^{-12}$

V2TG	0.018	0.49	$7.88x10^{-12}$
PE.P (18:1/22:6)	0.047	-0.49	$1.14x10^{-11}$
TPCH	0.048	-0.48	$1.48x10^{-11}$
CE (22:5)	0.043	-0.48	$1.51x10^{-11}$
LCER (26:1)	0.041	-0.48	$1.85x10^{-11}$
PS (20:0/22:5)	0.049	-0.48	$3.29x10^{-11}$
MAG (16:1)	0.011	0.47	$5.62x10^{-11}$
TPTG	0.013	0.47	$5.75x10^{-11}$
Glucose	0.024	0.47	$5.94x10^{-11}$
LCER (24:1)	0.037	-0.47	$5.94x10^{-11}$
V3PL	0.025	0.47	$6.29x10^{-11}$
Methionine	0.016	-0.47	$7.06x10^{-11}$
HDTG	0.021	0.47	$7.51x10^{-11}$
PE (18:1/18:3)	0.029	-0.46	$1.31x10^{-10}$
L3TG	0.031	0.46	$1.52x10^{-10}$
H1FC	0.043	-0.46	$1.71x10^{-10}$
PC (16:0/20:2)	0.045	-0.46	$1.88x10^{-10}$
MAG (22:6)	0.012	0.46	$2.04x10^{-10}$
HCER (26:1)	0.036	-0.45	$2.71x10^{-10}$
CE (20:0)	0.035	-0.45	$2.74x10^{-10}$
PE.P (18:0/22:6)	0.042	-0.45	$3.55x10^{-10}$
HCER.d (18:0/24:1)	0.034	-0.45	$3.99x10^{-10}$
V5TG	0.024	0.45	$5.21x10^{-10}$
HCER (18:0)	0.032	-0.44	$6.39x10^{-10}$
PC (16:0/22:5)	0.042	-0.44	$7.67x10^{-10}$
SM (18:1)	0.042	-0.44	$9.01x10^{-10}$
SM (26:1)	0.035	-0.44	$1.00x10^{-9}$
L4CH	0.041	-0.44	$1.26x10^{-9}$
MAG (20:2)	0.011	0.43	$1.57x10^{-9}$
H2FC	0.039	-0.43	$1.62x10^{-9}$
L1CH	0.028	-0.43	$1.77x10^{-9}$
PS (20:0/20:3)	0.040	-0.43	$1.82x10^{-9}$
CE (20:5)	0.035	-0.43	$2.84x10^{-9}$
Tryptophan	0.033	-0.42	$4.02x10^{-9}$
V3CH	0.023	0.42	$4.15x10^{-9}$
DAG (16:0/20:4)	0.008	0.42	$4.43x10^{-9}$
LPE (16:1)	0.025	-0.42	$6.29x10^{-9}$

PE.O (18:0/22:6)	0.037	-0.41	$8.67x10^{-9}$
CER (22:1)	0.022	0.41	$9.89x10^{-9}$
V4FC	0.026	0.41	$1.21x10^{-8}$
VLTG	0.010	0.41	$1.34x10^{-8}$
PE (18:0/22:6)	0.015	0.41	$1.38x10^{-8}$
PE (18:2/18:3)	0.020	-0.41	$1.39x10^{-8}$
LPE (22:4)	0.021	-0.41	$1.41x10^{-8}$
LPC (16:0)	0.046	-0.41	$1.46x10^{-8}$
V2PL	0.016	0.41	$1.74x10^{-8}$
Acetone	0.015	0.40	$1.96x10^{-8}$
LDCH	0.037	-0.40	$2.88x10^{-8}$
CER (14:0)	0.040	-0.40	$3.99x10^{-8}$
DAG (16:0/18:1)	0.007	0.40	$4.05x10^{-8}$
PE.P (16:0/16:0)	0.033	-0.39	$5.80x10^{-8}$
3-Hydroxybutyric acid	0.022	0.39	$6.03x10^{-8}$
FFA (24:0)	0.033	-0.39	$6.05x10^{-8}$
PI (18:1/16:1)	0.032	-0.39	$7.33x10^{-8}$
Kynurenic acid	0.011	-0.39	$8.31x10^{-8}$
LCER (18:1)	0.028	-0.39	$8.90x10^{-8}$
IDPN	0.025	0.39	$8.98x10^{-8}$
IDAB	0.025	0.39	$9.00x10^{-8}$
VLCH	0.016	0.39	$9.20x10^{-8}$
PC (18:1/20:5)	0.024	-0.39	$9.86x10^{-8}$
HCER (24:1)	0.032	-0.38	$1.08x10^{-7}$
PE (16:0/18:1)	0.012	0.38	$1.09x10^{-7}$
PS (20:0/22:4)	0.027	-0.38	$1.16x10^{-7}$
DCER (18:0)	0.025	0.38	$1.17x10^{-7}$
LPC (16:1)	0.035	-0.38	$1.26x10^{-7}$
LPG (18:1)	0.025	-0.38	$1.34x10^{-7}$
PE.P (18:0/16:1)	0.037	-0.38	$1.84x10^{-7}$
L4PL	0.037	-0.38	$2.00x10^{-7}$
PG (18:1/20:4)	0.028	0.38	$2.18x10^{-7}$
DAG (18:1/20:4)	0.011	0.37	$2.43x10^{-7}$
DAG (16:0/22:6)	0.008	0.37	$2.46x10^{-7}$
PS (18:0/18:1)	0.013	0.37	$2.66x10^{-7}$
TAG (55:5)_FA (20:4)	0.008	0.37	$2.70x10^{-7}$
3-Hydroxykynurenine	0.019	0.37	$3.60x10^{-7}$

V2CH	0.015	0.37	$3.84x10^{-7}$
L4FC	0.037	-0.37	$4.70x10^{-7}$
PE (18:0/22:4)	0.025	-0.36	$5.13x10^{-7}$
PE (18:1/20:4)	0.031	-0.36	$5.47x10^{-7}$
PI (16:0/22:4)	0.032	-0.36	$6.05x10^{-7}$
MAG (18:0)	0.017	0.36	$7.02x10^{-7}$
V4CH	0.025	0.36	$7.26x10^{-7}$
LPE (22:5)	0.013	-0.36	$7.64x10^{-7}$
L3CH	0.032	-0.36	$8.91x10^{-7}$
PI (18:0/18:3)	0.023	-0.36	$8.99x10^{-7}$
PI (16:0/18:2)	0.034	-0.35	$1.10x10^{-6}$
PI (18:0/18:2)	0.036	-0.35	$1.35x10^{-6}$
TAG (54:4)_FA (20:4)	0.010	0.35	$1.40x10^{-6}$
TAG (54:5)_FA (20:4)	0.013	0.35	$1.53x10^{-6}$
PC (18:1/22:5)	0.036	-0.35	$2.02x10^{-6}$
LPE (16:0)	0.029	-0.34	$2.90x10^{-6}$
PC (18:0/18:3)	0.011	-0.34	$2.99x10^{-6}$
CER (20:1)	0.019	0.34	$3.23x10^{-6}$
CER (22:0)	0.032	-0.34	$3.43x10^{-6}$
DAG (16:0/18:2)	0.002	0.33	$4.63x10^{-6}$
LCER (20:0)	0.027	-0.33	$5.13x10^{-6}$
DAG (18:2/22:4)	0.007	0.33	$5.14x10^{-6}$
HCER (16:0)	0.026	-0.33	$5.29x10^{-6}$
CE (22:4)	0.026	-0.33	$5.39x10^{-6}$
PG (20:0/22:5)	0.012	-0.33	$5.69x10^{-6}$
TAG (54:5)_FA (16:0)	0.007	0.33	$5.91x10^{-6}$
TAG (52:4)_FA (20:4)	0.006	0.32	$1.06x10^{-5}$
PI (20:0/18:1)	0.022	-0.32	$1.10x10^{-5}$
PE (18:1/20:3)	0.028	-0.32	$1.18x10^{-5}$
TAG (56:6)_FA (18:0)	0.012	0.32	$1.23x10^{-5}$
TAG (54:6)_FA (20:4)	0.008	0.32	$1.25x10^{-5}$
TAG (56:5)_FA (20:4)	0.014	0.32	$1.30x10^{-5}$
VLFC	0.011	0.32	$1.44x10^{-5}$
TAG (54:6)_FA (16:0)	0.004	0.31	$1.98x10^{-5}$
IDTG	0.003	0.31	$2.54x10^{-5}$
CER (20:0)	0.013	0.31	$2.56x10^{-5}$
DAG (18:1/22:4)	0.007	0.31	$2.68x10^{-5}$

DAG (16:0/16:0)	0.004	0.31	$2.78x10^{-5}$
V1TG	0.001	0.31	$2.89x10^{-5}$
LDPL	0.029	-0.31	$2.91x10^{-5}$
DAG (18:2/20:4)	0.006	0.31	$2.93x10^{-}$
TAG (54:4)_FA (22:4)	0.002	0.30	$3.57x10^{-5}$
TAG (56:5)_FA (18:0)	0.012	0.30	$3.77x10^{-5}$
PI (16:0/18:0)	0.027	-0.30	$4.40x10^{-5}$
TAG (58:7)_FA (18:0)	0.006	0.30	$4.43x10^{-5}$
PG (18:1/20:2)	0.027	-0.30	$4.77x10^{-5}$
CE (16:1)	0.024	-0.30	$5.97x10^{-5}$
PC (16:0/16:1)	0.023	-0.29	$6.60x10^{-5}$
SM (18:0)	0.024	-0.29	$6.88x10^{-5}$
TAG (56:4)_FA (20:4)	0.009	0.29	$6.88x10^{-5}$
DAG (18:1/22:6)	0.008	0.29	$8.08x10^{-5}$
H2A2	0.029	-0.29	$8.36x10^{-5}$
TAG (58:6)_FA (18:0)	0.003	0.29	$8.82x10^{-5}$
PI (18:0/20:5)	0.014	-0.29	$9.04x10^{-5}$
DAG (16:0/20:5)	0.004	0.29	$1.05x10^{-4}$
CER (24:1)	0.009	0.29	$1.09x10^{-4}$
Citric acid	0.020	0.28	$1.10x10^{-4}$
L3PL	0.026	-0.28	$1.19x10^{-4}$
DAG (16:0/22:5)	0.005	0.28	$1.21x10^{-4}$
TAG (54:5)_FA (22:5)	0.003	0.28	$1.29x10^{-4}$
Asparagine	0.010	-0.28	$1.34x10^{-4}$
MAG (14:0)	0.008	0.28	$1.43x10^{-4}$
PG (18:2/16:1)	0.028	0.28	$1.59x10^{-4}$
H2CH	0.031	-0.28	$1.59x10^{-4}$
FFA (20:4)	0.008	0.28	$1.59x10^{-4}$
PI (18:0/22:5)	0.030	-0.28	$1.61x10^{-4}$
V1PL	0.001	0.28	$1.72x10^{-4}$
VLPL	0.009	0.28	$1.92x10^{-4}$
LDFC	0.027	-0.27	$2.18x10^{-4}$
PG (20:0/20:4)	0.011	-0.27	$2.24x10^{-4}$
PS (20:0/16:1)	0.015	-0.27	$2.42x10^{-4}$
L1FC	0.018	-0.27	$2.53x10^{-4}$
TAG (53:5)_FA (20:4)	0.002	0.27	$2.74x10^{-4}$
DAG (18:0/18:1)	0.004	0.27	$2.89x10^{-4}$

TAG (58:8)_FA (20:3)	0.030	-0.27	$3.20x10^{-4}$
Indole-3-acetic acid	0.008	-0.26	$3.63x10^{-4}$
TAG (56:6)_FA (20:4)	0.006	0.26	$3.66x10^{-4}$
L4PN	0.028	-0.26	$4.34x10^{-4}$
IDCH	0.014	0.26	$4.34x10^{-4}$
L1PL	0.013	-0.26	$4.34x10^{-4}$
L4AB	0.028	-0.26	$4.34x10^{-4}$
CER (16:0)	0.013	0.26	$4.43x10^{-4}$
MAG (18:3)	0.010	0.26	$4.63x10^{-4}$
DAG (16:0/16:1)	0.001	0.26	$4.74x10^{-4}$
CE (22:2)	0.023	-0.26	$5.08x10^{-4}$
Kynurenine	0.021	0.25	$6.21x10^{-4}$
PE (16:0/20:5)	0.020	-0.25	$6.29x10^{-4}$
CE (22:6)	0.028	-0.25	$6.33x10^{-4}$
PI (16:0_22:6)	0.025	-0.25	$6.34x10^{-4}$
TAG (58:6)_FA (22:4)	0.004	0.25	$7.31x10^{-4}$
Creatine	0.015	0.25	$7.31x10^{-4}$
TAG (51:1)_FA (18:1)	0.001	0.25	$8.20x10^{-4}$
DAG (18:1/18:1)	0.004	0.25	$8.28x10^{-4}$
DAG (16:1/20:4)	0.004	0.25	$8.99x10^{-4}$
L2CH	0.016	-0.24	$1.10x10^{-3}$
TAG (50:1)_FA (18:1)	0.006	0.24	$1.21x10^{-3}$
TAG (51:1)_FA (17:0)	0.000	0.24	$1.21x10^{-3}$
TAG (54:3)_FA (18:0)	0.005	0.24	$1.36x10^{-3}$
L3FC	0.024	-0.24	$1.43x10^{-3}$
DAG (18:0/18:2)	0.000	0.24	$1.53x10^{-3}$
DAG (16:1/18:2)	0.000	0.24	$1.63X10^{-3}$
TAG (51:1)_FA (16:0)	0.001	0.23	$1.78x10^{-3}$
TAG (56:5)_FA (22:4)	0.002	0.23	$1.79x10^{-3}$
TAG (54:8)_FA (18:3)	0.012	-0.23	$1.80x10^{-3}$
TAG (51:2)_FA (17:0)	0.001	0.23	$1.82x10^{-3}$
PE.O (18:0/18:1)	0.015	0.23	$1.96x10^{-3}$
TAG (50:2)_FA (16:0)	0.004	0.23	$2.29x10^{-3}$
LPE (18:0)	0.029	-0.23	$2.35x10^{-3}$
TAG (58:6)_FA (18:1)	0.003	0.23	$2.44x10^{-3}$
TAG (56:6)_FA (22:4)	0.000	0.23	$2.53x10^{-3}$
TAG (53:3)_FA (17:0)	0.002	0.23	$2.59x10^{-3}$

PE (18:0/20:0)	0.031	-0.23	2.69x10^{-3}
TAG (51:4)_FA (20:4)	0.002	0.23	2.70x10^{-3}
PI (18:0/22:6)	0.025	-0.23	2.71x10^{-3}
IDFC	0.011	0.22	2.82x10$^{-.3}$
TAG (50:2)_FA (18:2)	0.000	0.22	2.86x10^{-3}
TAG (58:6)_FA (22:5)	0.001	0.22	2.89x10^{-3}
DCER (20:0)	0.013	-0.22	3.09x10^{-3}
TAG (49:0)_FA (16:0)	0.000	0.22	3.09x10^{-3}
TAG (53:1)_FA (18:1)	0.000	0.22	3.09x10^{-3}
TAG (49:0)_FA (17:0)	0.002	0.22	3.19x10^{-3}
TAG (53:2)_FA (17:0)	0.001	0.22	3.20x10^{-3}
DAG (16:1/18:0)	0.003	0.22	3.39x10^{-3}
TAG (56:4)_FA (22:4)	0.000	0.22	3.44x10^{-3}
TAG (56:6)_FA (18:1)	0.001	0.22	3.73x10^{-3}
TAG (50:1)_FA (16:0)	0.004	0.22	3.75x10^{-3}
PC (18:0/16:1)	0.016	-0.22	4.20x10^{-3}
TAG (52:2)_FA (18:0)	0.002	0.22	4.22x10^{-3}
TAG (54:2)_FA (18:0)	0.004	0.22	4.22x10^{-3}
Isoleucine	0.011	-0.22	4.39x10^{-3}
TAG (53:2)_FA (18:1)	0.000	0.22	4.39x10^{-3}
DAG (18:0/18:3)	0.000	0.22	4.39x10^{-3}
TAG (48:5)_FA (18:3)	0.021	-0.21	4.43x10^{-3}
TAG (52:2)_FA (16:1)	0.001	0.21	4.82x10^{-3}
LCER (18:0)	0.018	-0.21	4.82x10^{-3}
TAG (51:2)_FA (18:2)	0.005	0.21	4.83x10^{-3}
TAG (58:8)_FA (18:1)	0.005	0.21	5.48x10^{-3}
PS (18:0/20:0)	0.019	0.21	5.63x10^{-3}
TAG (52:3)_FA (18:0)	0.000	0.21	6.00x10^{-3}
TAG (48:1)_FA (16:1)	0.001	0.21	6.48x10^{-3}
PG (20:0/16:1)	0.003	-0.21	6.58x10^{-3}
SM (22:0)	0.005	-0.21	6.84x10^{-3}
TAG (53:1)_FA (17:0)	0.001	0.20	7.18x10^{-3}
TAG (56:8)_FA (16:0)	0.003	0.20	7.91x10^{-3}
FFA (22:6)	0.012	-0.20	8.06x10^{-3}
TAG (56:5)_FA (16:0)	0.000	0.20	8.06x10^{-3}
FFA (20:1)	0.011	0.20	8.41x10^{-3}
TAG (50:2)_FA (16:1)	0.003	0.20	9.09x10^{-3}

DAG (18:2/22:5)	0.004	0.20	$9.37x10^{-3}$
PG (18:2/18:2)	0.001	-0.20	$9.51x10^{-3}$
PG (16:0/18:2)	0.031	-0.20	$9.56x10^{-3}$
HCER (20:1)	0.015	-0.20	$9.93x10^{-3}$
TAG (58:7)_FA (18:1)	0.002	0.20	$9.93x10^{-3}$
TAG (56:7)_FA (20:3)	0.025	-0.20	$9.98x10^{-3}$
TAG (52:1)_FA (18:1)	0.003	0.20	$1.00x10^{-2}$
TAG (51:1)_FA (18:0)	0.001	0.20	$1.03x10^{-2}$
PE (18:0/18:2)	0.020	-0.20	$1.05x10^{-2}$
TAG (53:1)_FA (18:0)	0.000	0.20	$1.05x10^{-2}$
TAG (52:3)_FA (20:0)	0.001	0.20	$1.05x10^{-2}$
PC (18:1/20:2)	0.022	-0.19	$1.07x10^{-2}$
TAG (56:6)_FA (16:0)	0.002	0.19	$1.10x10^{-2}$
TAG (56:6)_FA (20:3)	0.025	-0.19	$1.10x10^{-2}$
TAG (56:7)_FA (20:4)	0.001	0.19	$1.15x10^{-2}$
TAG (54:3)_FA (18:1)	0.002	0.19	$1.21x10^{-2}$
DAG (16:1/22:6)	0.003	0.19	$1.23x10^{-2}$
PI (16:0/22:5)	0.022	-0.19	$1.26x10^{-2}$
TAG (56:6)_FA (22:6)	0.002	0.19	$1.27x10^{-2}$
TAG (54:4)_FA (18:0)	0.000	0.19	$1.27x10^{-2}$
PI (18:0/18:0)	0.015	-0.19	$1.31x10^{-2}$
TAG (49:1)_FA (18:1)	0.004	0.19	$1.32x10^{-2}$
PE (18:0/20:1)	0.021	-0.19	$1.32x10^{-2}$
TAG (56:2)_FA (20:0)	0.016	-0.19	$1.32x10^{-2}$
TAG (52:1)_FA (16:0)	0.003	0.19	$1.36x10^{-2}$
PE (18:1/18:1)	0.007	0.19	$1.40x10^{-2}$
TAG (53:2)_FA (18:2)	0.004	0.19	$1.43x10^{-2}$
PG (20:0/18:1)	0.007	-0.19	$1.43x10^{-2}$
TAG (50:4)_FA (20:3)	0.020	-0.19	$1.54x10^{-2}$
TAG (54:2)_FA (18:1)	0.002	0.18	$1.61x10^{-2}$
TAG (49:1)_FA (16:1)	0.002	0.18	$1.64x10^{-2}$
LDHD	0.011	0.18	$1.66x10^{-2}$
TAG (51:2)_FA (18:1)	0.003	0.18	$1.73x10^{-2}$
TAG (56:3)_FA (20:0)	0.019	-0.18	$1.73x10^{-2}$
TAG (48:0)_FA (16:0)	0.000	0.18	$1.82x10^{-2}$
TAG (48:4)_FA (18:3)	0.019	-0.18	$1.82x10^{-2}$
PE (18:0/20:4)	0.002	0.18	$1.84x10^{-2}$

TAG (52:1)_FA (18:0)	0.004	0.18	$1.84x10^{-2}$
TAG (54:3)_FA (18:2)	0.003	0.18	$1.84x10^{-2}$
TAG (51:2)_FA (16:0)	0.004	0.18	$1.87x10^{-2}$
TAG (54:6)_FA (18:3)	0.021	-0.18	$1.91x10^{-2}$
TAG (56:9)_FA (18:3)	0.013	-0.18	$1.91x10^{-2}$
TAG (58:7)_FA (22:4)	0.001	0.18	$1.91x10^{-2}$
V5FC	0.007	-0.18	$1.92x10^{-2}$
TPAB	0.004	0.18	$1.93x10^{-2}$
TBPN	0.004	0.18	$1.93x10^{-2}$
LCER.d (18:0/20:0)	0.015	-0.18	$1.95x10^{-2}$
TAG (55:3)_FA (18:1)	0.000	0.18	$1.97x10^{-2}$
TAG (51:2)_FA (16:1)	0.002	0.18	$1.97x10^{-2}$
TAG (58:9)_FA (18:1)	0.000	0.18	$2.00x10^{-2}$
TAG (56:7)_FA (22:6)	0.002	0.18	$2.03x10^{-2}$
TAG (49:1)_FA (16:0)	0.005	0.18	$2.05x10^{-2}$
TAG (51:0)_FA (16:0)	0.001	0.18	$2.06x10^{-2}$
PE (16:0/18:3)	0.008	-0.18	$2.08x10^{-2}$
V5CH	0.009	0.18	$2.13x10^{-2}$
TAG (53:4)_FA (20:4)	0.001	0.18	$2.16x10^{-2}$
TAG (53:3)_FA (18:2)	0.006	0.18	$2.17x10^{-2}$
Valine	0.006	-0.18	$2.19x10^{-2}$
LPE (22:6)	0.013	-0.18	$2.25x10^{-2}$
TAG (52:5)_FA (20:5)	0.001	0.17	$2.27x10^{-2}$
TAG (58:7)_FA (22:6)	0.001	0.17	$2.29x10^{-2}$
Lysine	0.006	-0.17	$2.31x10^{-2}$
TAG (54:5)_FA (22:4)	0.003	0.17	$2.33x10^{-2}$
TAG (52:2)_FA (18:2)	0.003	0.17	$2.33x10^{-2}$
TAG (53:2)_FA (16:0)	0.002	0.17	$2.33x10^{-2}$
TAG (47:0)_FA (16:0)	0.003	0.17	$2.35x10^{-2}$
TAG (51:0)_FA (17:0)	0.002	0.17	$2.35x10^{-2}$
DAG (16:0/18:0)	0.002	0.17	$2.48x10^{-2}$
TAG (52:5)_FA (20:4)	0.004	0.17	$2.55x10^{-2}$
TAG (52:5)_FA (20:3)	0.023	-0.17	$2.56x10^{-2}$
DAG (16:0/18:3)	0.003	0.17	$2.61x10^{-2}$
TAG (51:0)_FA (18:0)	0.002	0.17	$2.61x10^{-2}$
L2PL	0.009	-0.17	$2.63x10^{-2}$
TAG (58:8)_FA (22:6)	0.002	0.17	$2.63x10^{-2}$

PG (18:1/20:3)	0.019	0.17	$2.65x10^{-2}$
TAG (58:8)_FA (20:4)	0.001	0.17	$2.82x10^{-2}$
TAG (58:7)_FA (18:2)	0.004	0.17	$2.90x10^{-2}$
V5PL	0.008	0.17	$3.05x10^{-2}$
TAG (52:3)_FA (18:1)	0.000	0.17	$3.19x10^{-2}$
TAG (50:5)_FA (18:3)	0.019	-0.16	$3.24x10^{-2}$
TAG (54:7)_FA (18:3)	0.017	-0.16	$3.28x10^{-2}$
TAG (53:4)_FA (17:0)	0.004	0.16	$3.34x10^{-2}$
DAG (18:1/20:1)	0.002	0.16	$3.44x10^{-2}$
TAG (56:7)_FA (18:1)	0.002	0.16	$3.47x10^{-2}$
TAG (52:3)_FA (16:1)	0.002	0.16	$3.60x10^{-2}$
PC (18:0/22:5)	0.002	0.16	$3.63x10^{-2}$
L2FC	0.010	-0.16	$3.65x10^{-2}$
TAG (49:2)_FA (18:2)	0.006	0.16	$3.69x10^{-2}$
TAG (53:6)_FA (20:4)	0.006	0.16	$3.76x10^{-2}$
Creatinine	0.018	0.16	$3.77x10^{-2}$
TAG (50:5)_FA (14:0)	0.019	-0.16	$3.83x10^{-2}$
TAG (50:3)_FA (16:0)	0.003	0.16	$4.01x10^{-2}$
TAG (50:1)_FA (16:1)	0.002	0.16	$4.09x10^{-2}$
V1CH	0.003	0.16	$4.13x10^{-2}$
TAG (58:10)_FA (20:5)	0.006	-0.16	$4.13x10^{-2}$
L5CH	0.018	-0.16	$4.32x10^{-2}$
TAG (49:2)_FA (16:0)	0.006	0.16	$4.38x10^{-2}$
TAG (51:3)_FA (17:0)	0.003	0.16	$4.37x10^{-2}$
TAG (54:4)_FA (18:1)	0.002	0.16	$4.38x10^{-2}$
H1CH	0.023	-0.16	$4.40x10^{-2}$
TAG (54:8)_FA (18:2)	0.009	-0.16	$4.49x10^{-2}$
PE (18:1/22:6)	0.003	0.16	$4.52x10^{-2}$
PE (18:1/20:1)	0.016	-0.16	$4.53x10^{-2}$
DAG (18:0/22:6)	0.002	0.15	$4.61x10^{-2}$
TAG (49:0)_FA (18:0)	0.000	0.15	$4.65x10^{-2}$
TAG (56:7)_FA (16:0)	0.004	0.15	$4.72x10^{-2}$
Ornithine	0.010	0.15	$4.73x10^{-2}$
PS (18:1/18:2)	0.001	0.15	$4.75x10^{-2}$

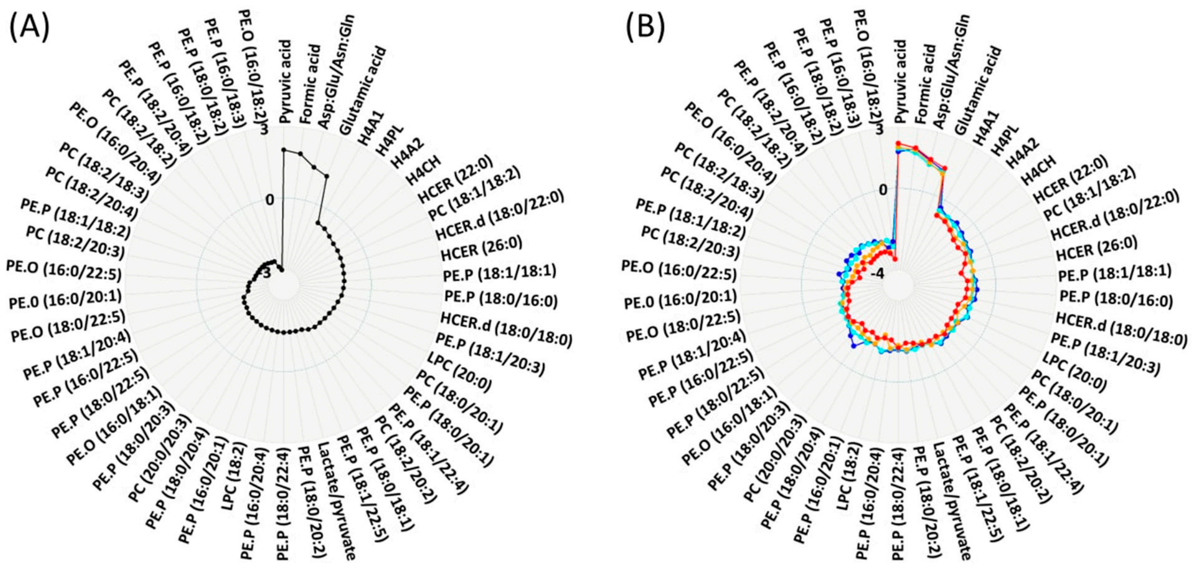

Figure S1 - (A) Radar plot showing the $\log_2$ fold change of the top 50 metabolites, lipoproteins and lipids with the most significant p-values for the control versus SARS-CoV-2 positive patients. (B) Radar plot showing the $\log_2$ fold change of the top 50 metabolites, lipoproteins and lipids with the most significant p-values for the control versus SARS-CoV-2 positive model but coloured by severity class. Control vs group B severity $\log_2$ fold change (blue), Control vs group C severity $\log_2$ fold change (cyan), Control vs group D severity $\log_2$ fold change (orange), Control vs group E severity $\log_2$ fold change (red). The radar plots are ordered by fold change.

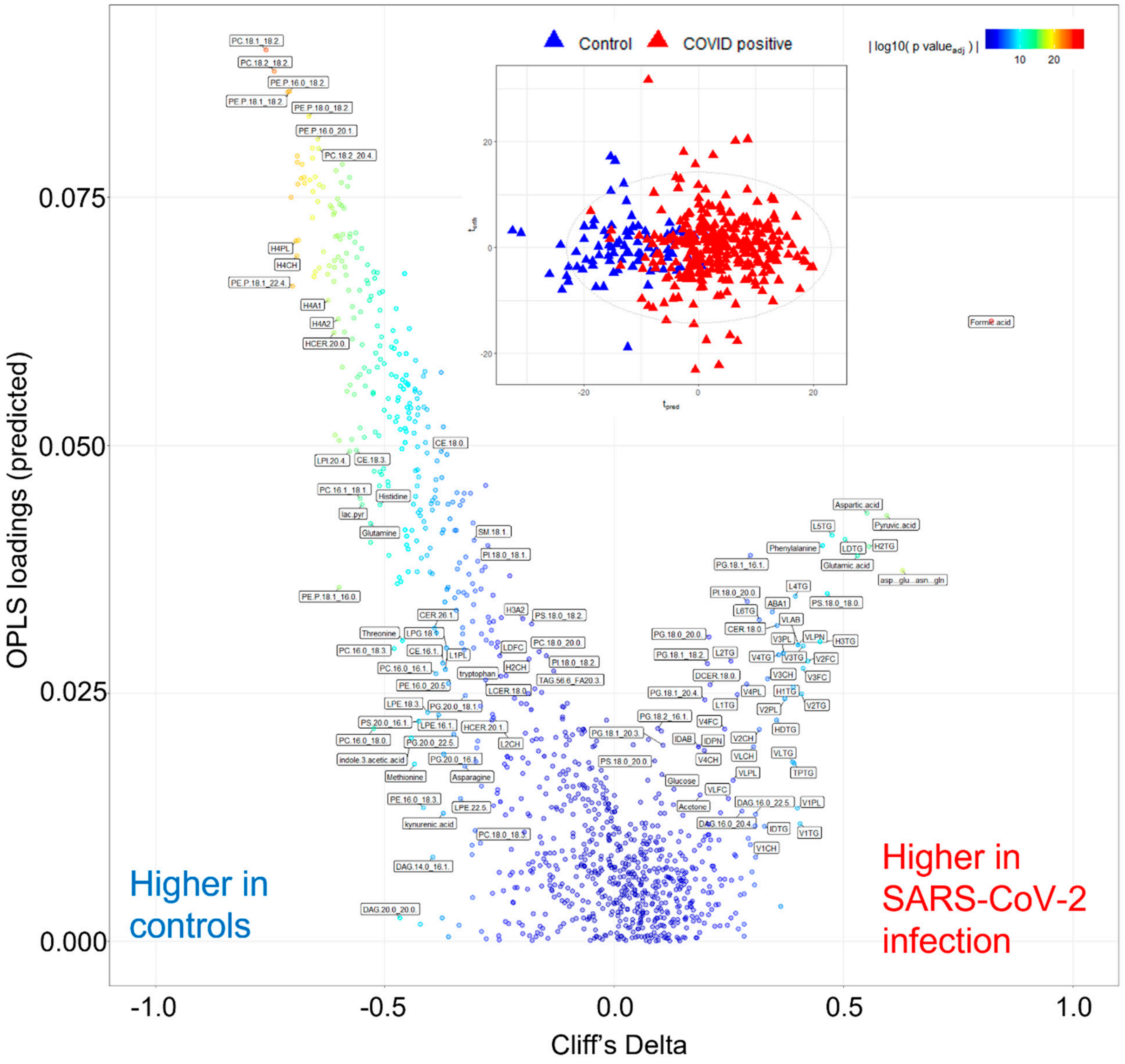

Figure S2 - Orthogonal Partial Least Squared (O-PLS-DA) and eruption plots between controls and SARS-CoV-2 positive patients of the integrative pan-metabolic model after correction for age. Eruption plot of the controls vs SARS-CoV-2 positive patients with the O-PLS-DA of controls (blue triangles) and SARS-CoV-2 positive patients (red triangles) (R_2X=0.10, AUROC=0.94) inset.

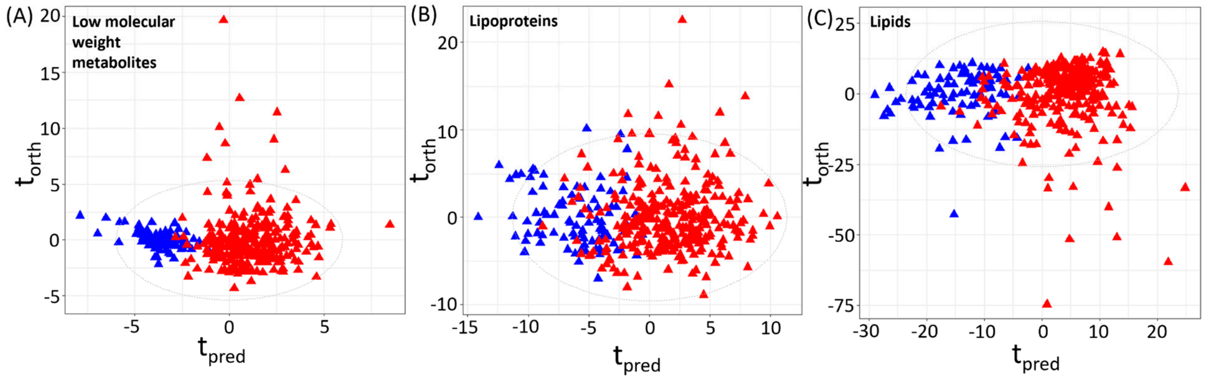

Figure S3 – O-PLS-DA scores plots of the low molecular weight metabolites, lipoproteins and lipids. (A) O-PLS-DA of control samples (blue triangles) and SARS-CoV-2 positive patients (red triangles) for the low molecular weight metabolites, $R_2X=0.12$, AUROC=1.00. (B) O-PLS-DA of control samples (blue triangles) and SARS-CoV-2 positive patients (red triangles) for the NMR derived lipoproteins, $R_2X=0.26$, AUROC=0.95. (C) O-PLS-DA of control samples (blue triangles) and SARS-CoV-2 positive patients (red triangles) for the MS derived lipids, $R_2X=0.17$, AUROC=0.98.

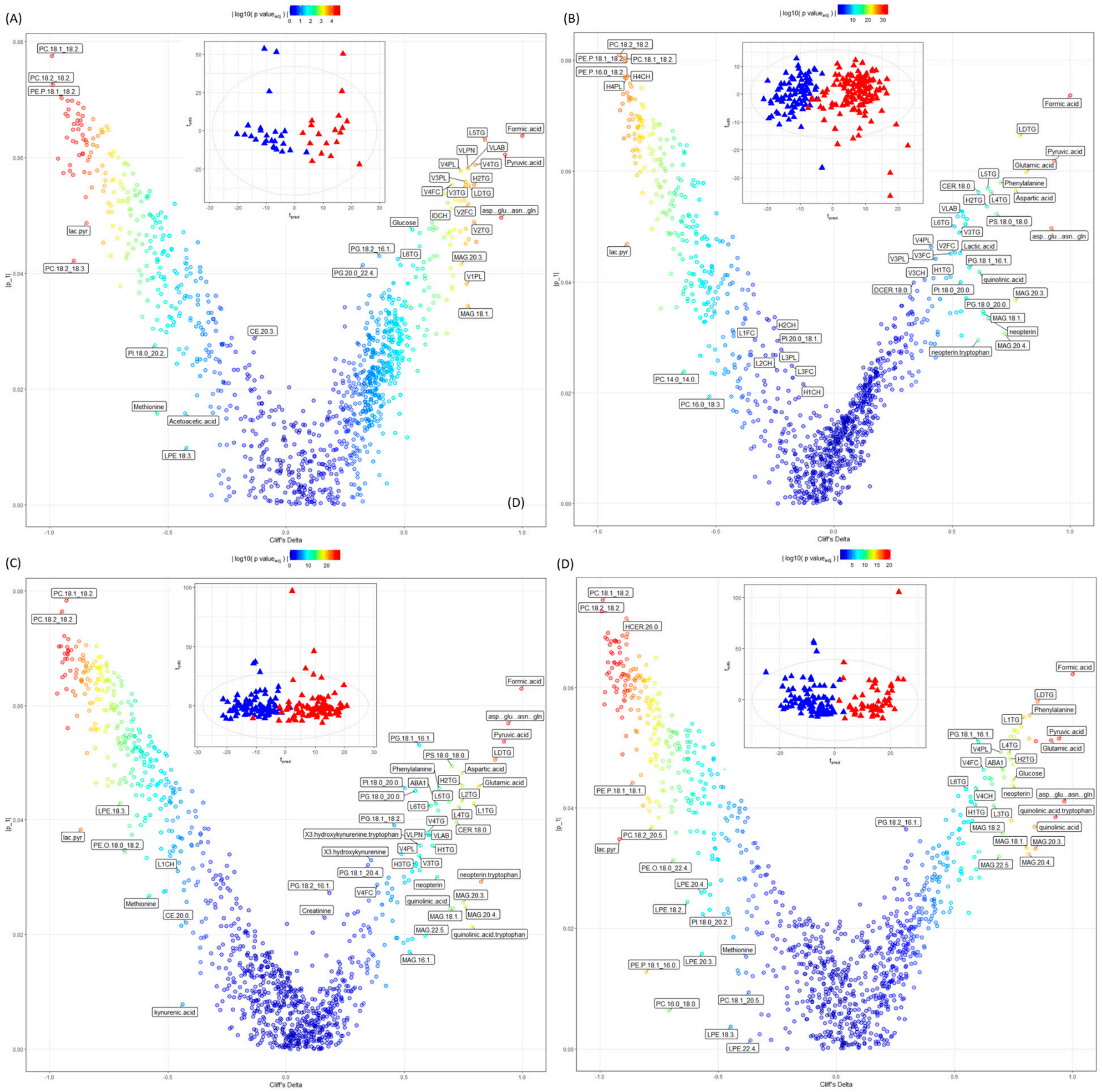

Figure S4 - Orthogonal Partial Least Squared (O-PLS-DA) and eruption plots between controls and SARS-CoV-2 positive patients of each severity group for the four Integrated assays. (A) Eruption plot of the controls vs group B severity group with the O-PLS-DA of controls (blue triangles) and SARS-CoV-2 group B severity patients (red triangles) (R_2X=0.21, AUROC=0.99) inset. (B) Eruption plot of the controls vs group C severity group with the O-PLS-DA of controls (blue triangles) and SARS-CoV-2 group B severity patients (red triangles) (R_2X=0.12, AUROC=0.99) inset. (C) Eruption plot of the controls vs group D severity group with the O-PLS-DA of controls (blue triangles) and SARS-CoV-2 group B severity patients (red triangles) (R_2X=0.20, AUROC=0.99) inset. (D) Eruption plot of the controls vs group E severity group with the O-PLS-DA of controls (blue triangles) and SARS-CoV-2 group B severity patients (red triangles) (R_2X=0.22, AUROC=1.00) inset.

Table S6 - OPLS loadings, Cliff's delta and adjusted p-values of all the assays combined for the comparison of the controls and SARS-CoV-2 positive patients in severity class B. Only the 50 most significant metabolites are shown.

Metabolite	OPLS loadings	Cliff's delta	Adjusted p-value
Formic acid	0.064	1.00	$1.59x10^{-5}$
PC (18:1/18:2)	0.078	-0.99	$1.59x10^{-5}$
PC (18:2/18:2)	0.073	-0.99	$1.59x10^{-5}$
PE.O (16:0/18:2)	0.060	-0.96	$2.37x10^{-5}$
PE.P (18:1/18:2)	0.070	-0.95	$2.85x10^{-5}$
Pyruvic acid	0.061	0.93	$2.86x10^{-5}$
LPC (18:2)	0.057	-0.94	$2.86x10^{-5}$
PE.P (16:0/18:2)	0.070	-0.92	$2.86x10^{-5}$
PE.P (18:0/18:2)	0.068	-0.93	$2.86x10^{-5}$
PE.P (18:0/20:1)	0.067	-0.92	$2.86x10^{-5}$
PE.P (18:2/20:4)	0.063	-0.93	$2.86x10^{-5}$
HCER.d (18:0/18:0)	0.068	-0.92	$2.92x10^{-5}$
PE.O (16:0/20:4)	0.059	-0.92	$2.92x10^{-5}$
PE.P (16:0/18:3)	0.058	-0.92	$2.92x10^{-5}$
Asp:Glu/Asn/Gln	0.050	0.91	$3.09x10^{-5}$
PC (18:2/20:2)	0.057	-0.90	$3.16x10^{-5}$
PE.O (18:0/22:5)	0.065	-0.90	$3.16x10^{-5}$
PE.P (18:1/20:1)	0.070	-0.91	$3.16x10^{-5}$
PE.P (18:0/20:4)	0.063	-0.90	$3.16x10^{-5}$
PC (18:2/18:3)	0.042	-0.90	$3.25x10^{-5}$
PC (18:2/20:4)	0.066	-0.90	$3.25x10^{-5}$
HCER (22:0)	0.063	-0.89	$3.99x10^{-5}$
PC (16:0/18:2)	0.066	-0.88	$4.15x10^{-5}$
PE.O (16:0/20:1)	0.066	-0.88	$4.15x10^{-5}$
PC (20:0/20:4)	0.065	-0.88	$4.18x10^{-5}$
PE.P (18:0/18:1)	0.068	-0.88	$4.18x10^{-5}$
PE.P (18:1/22:5)	0.069	-0.88	$4.18x10^{-5}$
PE.O (18:0/22:4)	0.060	-0.88	$4.56x10^{-5}$
H4CH	0.066	-0.87	$4.99x10^{-5}$
PC (18:0/20:1)	0.066	-0.87	$5.44x10^{-5}$
PE.O (16:0/18:1)	0.061	-0.86	$5.44x10^{-5}$
PE.P (16:0/22:5)	0.067	-0.86	$5.44x10^{-5}$
PE.P (18:0/20:4)	0.061	-0.86	$5.44x10^{-5}$

PE.P (18:1/22:4)	0.064	-0.86	$5.44x10^{-5}$
PE.P (18:0/22:5)	0.069	-0.86	$5.80x10^{-5}$
PE.P (18:2/18:2)	0.063	-0.86	$5.80x10^{-5}$
PE.O (16:0/22:5)	0.057	-0.85	$6.38x10^{-5}$
PE.O (16:0/22:6)	0.053	-0.85	$6.83x10^{-5}$
PE.P (18:0/22:4)	0.063	-0.85	$6.83x10^{-5}$
Lactate/pyruvate	0.049	-0.84	$7.15x10^{-5}$
PC (20:0/18:1)	0.062	-0.84	$7.15x10^{-5}$
PE.P (18:0/18:0)	0.063	-0.84	$7.15x10^{-5}$
L5TG	0.063	0.84	$7.41x10^{-5}$
PE.P (16:0/20:4)	0.059	-0.84	$7.69x10^{-5}$
HCER.d (18:0/22:0)	0.060	-0.83	$9.13x10^{-5}$
PE.P (16:0/20:1)	0.065	-0.83	$9.13x10^{-5}$
PE.P (18:1/16:0)	0.057	-0.83	$9.13x10^{-5}$
PE.P (18:0/16:0)	0.061	-0.83	$1.01x10^{-4}$
PC (20:0/20:3)	0.055	-0.82	$1.11x10^{-4}$
H4PL	0.063	-0.82	$1.17x10^{-4}$

Table S7 - OPLS loadings, Cliff's delta and adjusted p-values of all the assays combined for the comparison of the controls and SARS-CoV-2 positive patients in severity class C. Only the 50 most significant metabolites are shown.

Metabolite	OPLS loadings	Cliff's delta	Adjusted p-value
Formic acid	0.074	1.00	$4.47x10^{-34}$
Pyruvic acid	0.062	0.93	$8.99x10^{-30}$
Asp:Glu/Asn:Gln	0.050	0.92	$4.05x10^{-29}$
PC (18:2/18:2)	0.081	-0.91	$1.28x10^{-28}$
PE.O (16:0/20:4)	0.071	-0.91	$1.66x10^{-28}$
PE.O (16:0/18:2)	0.072	-0.89	$8.50x10^{-28}$
PE.P (18:2/20:4)	0.074	-0.89	$8.50x10^{-28}$
PE.P (16:0/22:5)	0.066	-0.89	$1.31x10^{-27}$
PE.P (16:0/18:2)	0.077	-0.89	$1.49x10^{-27}$
PE.P (18:1/18:2)	0.080	-0.89	$1.49x10^{-27}$
PC (18:1/18:2)	0.080	-0.88	$1.88x10^{-27}$
H4PL	0.077	-0.88	$3.71x10^{-27}$
Lactate/pyruvate	0.047	-0.88	$4.94x10^{-27}$
PE.P (18:0/18:2)	0.074	-0.88	$4.94x10^{-27}$

H4CH	0.077	-0.87	$5.88x10^{-27}$
HCER.d (18:0/18:0)	0.073	-0.87	$1.24x10^{-26}$
PE.O (16:0/18:1)	0.066	-0.87	$1.24x10^{-26}$
PE.O (16:0/20:1)	0.068	-0.87	$1.24x10^{-26}$
PE.P (18:0/18:1)	0.073	-0.87	$1.47x10^{-26}$
PE.P (18:0/22:5)	0.069	-0.87	$1.47x10^{-26}$
PE.P (18:0/20:4)	0.073	-0.87	$1.54x10^{-26}$
PC (18:2/20:2)	0.076	-0.86	$2.12x10^{-26}$
PE.P (16:0/20:1)	0.073	-0.86	$3.91x10^{-26}$
LPC (18:2)	0.075	-0.86	$4.11x10^{-26}$
PC (18:2/20:3)	0.070	-0.86	$4.22x10^{-26}$
PE.P (16:0/20:4)	0.069	-0.85	$7.46x10^{-26}$
PE.O (18:0/22:5)	0.064	-0.85	$7.52x10^{-26}$
PC (20:0/20:3)	0.067	-0.84	$2.15x10^{-25}$
PE.P (18:0/22:4)	0.063	-0.84	$2.15x10^{-25}$
PE.P (18:2/18:2)	0.073	-0.84	$2.54x10^{-25}$
PC (18:0/20:1)	0.066	-0.84	$2.68x10^{-25}$
PE.O (18:0/22:4)	0.061	-0.84	$2.72x10^{-25}$
PE.P (18:0/20:1)	0.070	-0.84	$4.69x10^{-25}$
LPI (18:1)	0.064	-0.84	$4.87x10^{-25}$
LPI (18:2)	0.059	-0.83	$6.88x10^{-25}$
PE.P (18:1/22:5)	0.068	-0.83	$9.11x10^{-25}$
HCER (26:0)	0.068	-0.83	$1.15x10^{-24}$
PE.P (18:0/20:3)	0.068	-0.83	$1.20x10^{-24}$
PE.P (16:0/18:3)	0.064	-0.83	$1.36x10^{-24}$
PE.P (18:0/20:4)	0.069	-0.83	$1.97x10^{-24}$
PE.O (16:0/22:5)	0.066	-0.82	$2.14x10^{-24}$
PE.P (18:0/20:2)	0.070	-0.82	$2.14x10^{-24}$
HCER (22:0)	0.066	-0.82	$2.54x10^{-24}$
HCER.d (18:0/22:0)	0.065	-0.82	$2.54x10^{-24}$
LPI (20:3)	0.063	-0.82	$2.65x10^{-24}$
PE.P (18:0/18:0)	0.070	-0.82	$2.65x10^{-24}$
PC (20:0/18:1)	0.060	-0.82	$4.46x10^{-24}$
PE.P (18:1/18:1)	0.069	-0.82	$4.56x10^{-24}$
PC (18:2/20:4)	0.068	-0.82	$5.19x10^{-24}$
LPC (20:0)	0.075	-0.82	$6.05x10^{-24}$

Table S8 - OPLS loadings, Cliff's delta and adjusted p-values of all the assays combined for the comparison of the controls and SARS-CoV-2 positive patients in severity class D. Only the 50 most significant metabolites are shown.

Metabolite	OPLS loadings	Cliff's delta	Adjusted p-value
Formic acid	0.063	1.00	$5.46x10^{-28}$
PE.O (16:0/20:4)	0.062	-0.97	$1.14x10^{-26}$
H4CH	0.070	-0.96	$1.89x10^{-26}$
H4PL	0.070	-0.95	$4.13x10^{-26}$
PC (18:2/18:2)	0.076	-0.95	$5.90x10^{-26}$
PE.P (18:1/20:4)	0.068	-0.95	$5.90x10^{-26}$
PE.P (18:2/20:4)	0.065	-0.95	$5.90x10^{-26}$
Asp:Glu/Asn:Gln	0.057	0.94	$1.04x10^{-25}$
PE.P (18:1/18:2)	0.072	-0.94	$1.04x10^{-25}$
LPC (18:2)	0.069	-0.94	$1.27x10^{-25}$
PE.P (16:0/20:4)	0.065	-0.94	$1.41x10^{-25}$
PE.P (16:0/22:5)	0.068	-0.94	$1.41x10^{-25}$
PC (18:1/18:2)	0.078	-0.93	$2.41x10^{-25}$
PE.0 (16:0/18:2)	0.063	-0.93	$2.53x10^{-25}$
H4A2	0.068	-0.93	$3.02x10^{-25}$
PC (18:2/20:2)	0.069	-0.93	$3.02x10^{-25}$
H4A1	0.070	-0.93	$3.12x10^{-25}$
PE.P (18:0/18:2)	0.069	-0.92	$3.65x10^{-25}$
PC (18:2/18:3)	0.056	-0.92	$3.91x10^{-25}$
PE.P (16:0/18:2)	0.072	-0.92	$4.20x10^{-25}$
PE.P (18:0/22:5)	0.070	-0.92	$4.26x10^{-25}$
Pyruvic acid	0.054	0.92	$4.96x10^{-25}$
PC (18:2/20:3)	0.070	-0.92	$4.96x10^{-25}$
PE.P (18:2/18:2)	0.066	-0.91	$1.08x10^{-24}$
LPC (20:0)	0.070	-0.91	$1.59x10^{-24}$
PE.P (18:1/22:5)	0.068	-0.91	$2.88x10^{-24}$
PE.O (16:0/22:5)	0.063	-0.90	$4.36x10^{-24}$
PE.O (16:0/18:1)	0.062	-0.89	$1.31x10^{-23}$
PE.O (18:0/22:5)	0.065	-0.89	$1.55x10^{-23}$
PE.P (18:0/20:4)	0.066	-0.89	$1.69x10^{-23}$
PE.P (18:0/18:1)	0.070	-0.89	$1.73x10^{-23}$
PC (20:0/20:3)	0.066	-0.89	$1.89x10^{-23}$
Neopterin/tryptophan	0.029	0.82	$1.93x10^{-23}$

PE.P (16:0/18:3)	0.058	-0.89	$2.32x10^{-23}$
LDTG	0.051	0.88	$2.94x10^{-23}$
PC (18:2/20:4)	0.071	-0.88	$7.29x10^{-23}$
HCER.d (18:0/18:0)	0.068	-0.87	$9.77x10^{-23}$
Lactate/pyruvate	0.038	-0.87	$1.70x10^{-22}$
PE.O (16:0/20:1)	0.065	-0.87	$1.91x10^{-22}$
PI (20:0/18:2)	0.064	-0.87	$2.09x10^{-22}$
PE.P (18:1/22:4)	0.064	-0.87	$2.23x10^{-22}$
LPC (20:3)	0.068	-0.86	$3.99x10^{-22}$
PE.P (16:0/20:1)	0.069	-0.86	$3.99x10^{-22}$
PE.P (18:0/16:0)	0.068	-0.86	$5.19x10^{-22}$
PE.P (18:0/22:4)	0.066	-0.86	$5.53x10^{-22}$
PI (18:1/18:1)	0.062	-0.85	$1.04x10^{-21}$
TPA2	0.066	-0.85	$1.05x10^{-21}$
H4FC	0.067	-0.85	$1.41x10^{-21}$
PE.P (18:0/20:3)	0.064	-0.85	$1.55x10^{-21}$
SM (24:0)	0.067	-0.85	$1.55x10^{-21}$

Table S9 - OPLS loadings, Cliff's delta and adjusted p-values of all the assays combined for the comparison of the controls and SARS-CoV-2 positive patients in severity class E. Only the 50 most significant metabolites are shown.

Metabolite	OPLS loadings	Cliff's delta	Adjusted p-value
Formic acid	0.064	1.00	$2.50x10^{-21}$
PC (18:1/18:2)	0.075	-0.99	$2.67x10^{-21}$
PC (18:2/18:2)	0.073	-0.99	$2.67x10^{-21}$
PE.O (16:0/20:4)	0.057	-0.98	$3.92x10^{-21}$
PE.P (18:2/20:4)	0.059	-0.98	$3.92x10^{-21}$
H4PL	0.068	-0.98	$4.16x10^{-21}$
H4CH	0.068	-0.97	$7.25x10^{-21}$
PC (20:0/20:3)	0.062	-0.97	$7.25x10^{-21}$
PC (18:2/20:3)	0.064	-0.97	$8.31x10^{-21}$
PC (18:2/20:4)	0.069	-0.97	$8.31x10^{-21}$
Asp:Glu/Asn:Gln	0.041	0.97	$8.65x10^{-21}$
PE.P (18:1/20:4)	0.063	-0.96	$2.50x10^{-21}$
PE.P (16:0/18:2)	0.067	-0.96	$1.26x10^{-20}$
LPC (18:2)	0.062	-0.96	$1.73x10^{-20}$

TPA2	0.066	-0.95	1.81×10^{-20}
H4A1	0.068	-0.95	1.81×10^{-20}
PE.P (16:0/22:5)	0.063	-0.95	2.28×10^{-20}
H4A2	0.066	0.95	2.62×10^{-20}
Neopterin/tryptophan	0.051	0.84	4.83×10^{-20}
Pyruvic acid	0.052	0.94	4.93×10^{-20}
PE.P (18:1/18:2)	0.064	-0.94	4.93×10^{-20}
PE.P (16:0/20:4)	0.060	-0.94	5.06×10^{-20}
TPA1	0.068	-0.94	5.63×10^{-20}
PE.P (18:0/22:5)	0.064	-0.94	5.63×10^{-20}
PC (18:0/20:1)	0.068	-0.94	7.36×10^{-20}
HDA1	0.068	-0.93	8.26×10^{-20}
PE.P (18:2/18:2)	0.059	-0.93	1.00×10^{-20}
PE.P (18:0/20:3)	0.060	-0.93	1.17×10^{-19}
PE.P (18:0/20:4)	0.061	-0.93	1.17×10^{-19}
Quinolinic acid/tryptophan	0.038	0.93	1.19×10^{-19}
PE.P (18:0/18:2)	0.064	-0.93	1.19×10^{-19}
PE.O (16:0/22:5)	0.056	-0.93	1.29×10^{-19}
H4FC	0.068	-0.92	2.37×10^{-19}
PC (18:2/22:5)	0.064	-0.92	2.60×10^{-19}
HCER (22:0)	0.067	-0.92	2.62×10^{-19}
Lactate/pyruvate	0.035	-0.92	2.75×10^{-19}
HCER.d (18:0/22:0)	0.066	-0.92	3.11×10^{-19}
LPC (20:0)	0.064	-0.91	3.80×10^{-19}
PE.P (18:0/22:4)	0.062	-0.91	4.47×10^{-19}
PI (20:0/18:2)	0.057	-0.91	4.70×10^{-19}
Glutamic acid	0.051	0.91	5.54×10^{-19}
PE.O (16:0/18:2)	0.055	-0.91	6.52×10^{-19}
PE.P (16:0/18:3)	0.049	-0.91	6.86×10^{-19}
H3FC	0.069	-0.91	7.32×10^{-19}
HDA2	0.062	-0.91	7.33×10^{-19}
PE.P (16:0/20:1)	0.064	-0.90	9.68×10^{-19}
PC (18:2/22:6)	0.062	-0.90	1.06×10^{-18}
PC (18:2/20:2)	0.058	-0.90	1.12×10^{-18}
PE.O (16:0/20:1)	0.059	-0.90	1.37×10^{-18}
PE.P (18:1/22:4)	0.058	-0.90	1.50×10^{-18}

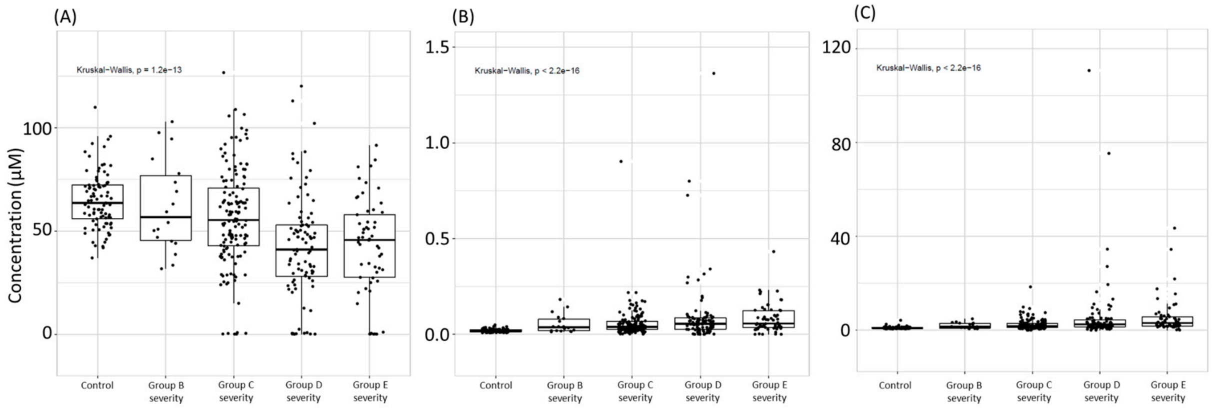

Figure S5 – Concentrations of metabolites for the controls and each severity class. (A) tryptophan, (B) neopterin and (C) quinolinic acid.

Section 4 - Severity group modeling

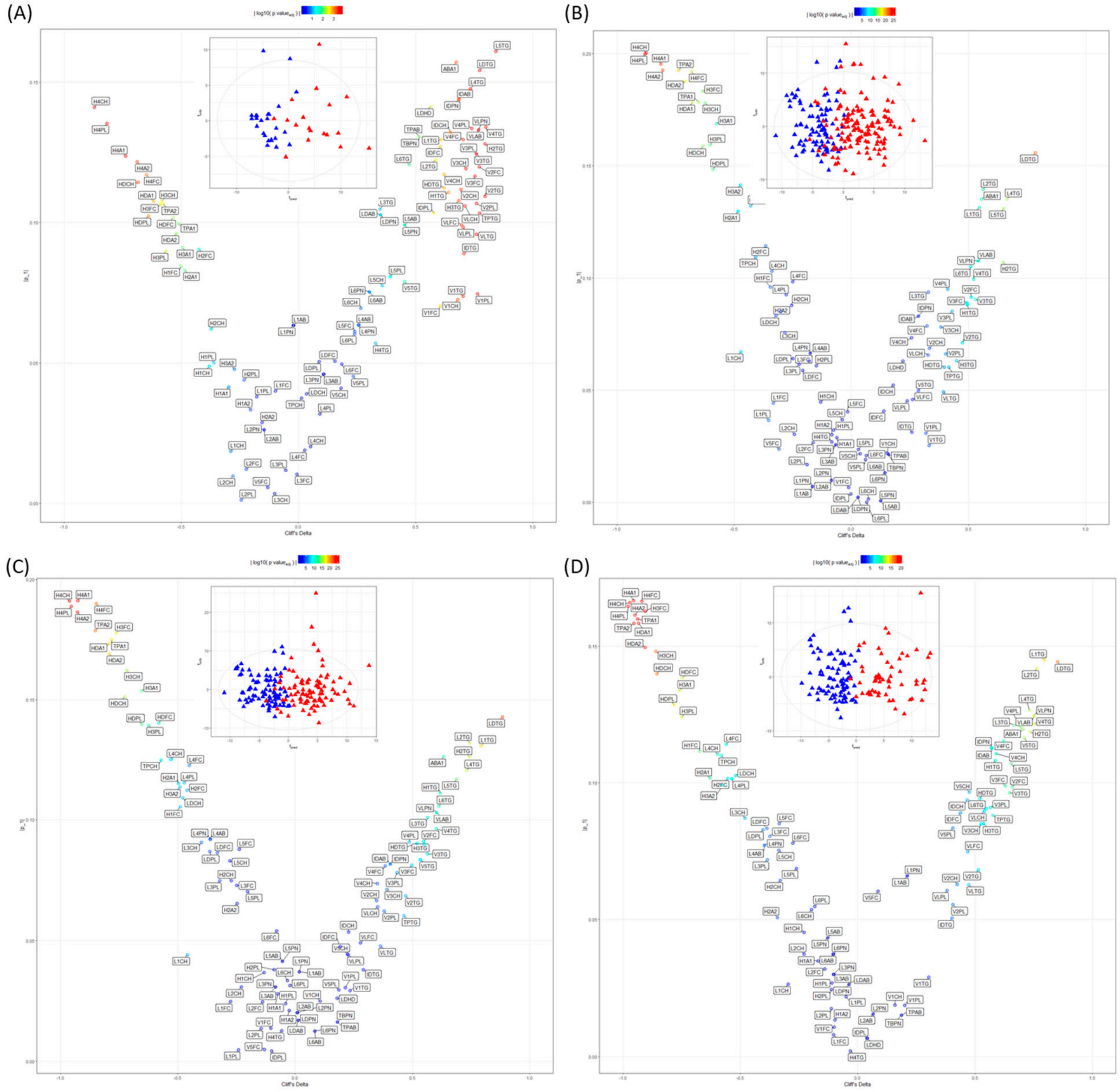

Figure S6 - Orthogonal Partial Least Squared (O-PLS-DA) and eruption plots between controls and SARS-CoV-2 positive patients of each severity group for the lipoproteins. (A) Eruption plot of the controls vs group B severity group with the O-PLS-DA of controls (blue triangles) and SARS-CoV-2 group B severity patients (red triangles) (R_2X=0.39, AUROC=0.91) inset. (B) Eruption plot of the controls vs group C severity group with the O-PLS-DA of controls (blue triangles) and SARS-CoV-2 group C severity patients (red triangles) (R_2X=0.27, AUROC=0.95) inset. (C) Eruption plot of the controls vs group D severity group with the O-PLS-DA of controls (blue triangles) and SARS-CoV-2 group D severity patients (red triangles) (R_2X=0.29, AUROC=0.97) inset. (D) Eruption plot of the controls vs group E severity group with the O-PLS-DA of controls (blue triangles) and SARS-CoV-2 group E severity patients (red triangles) (R_2X=0.36, AUROC=1.00) inset. Eruption plots are formed from the Cliff's delta (abscissa) and O-PLS-DA loadings (ordinate). Coloured by significance.

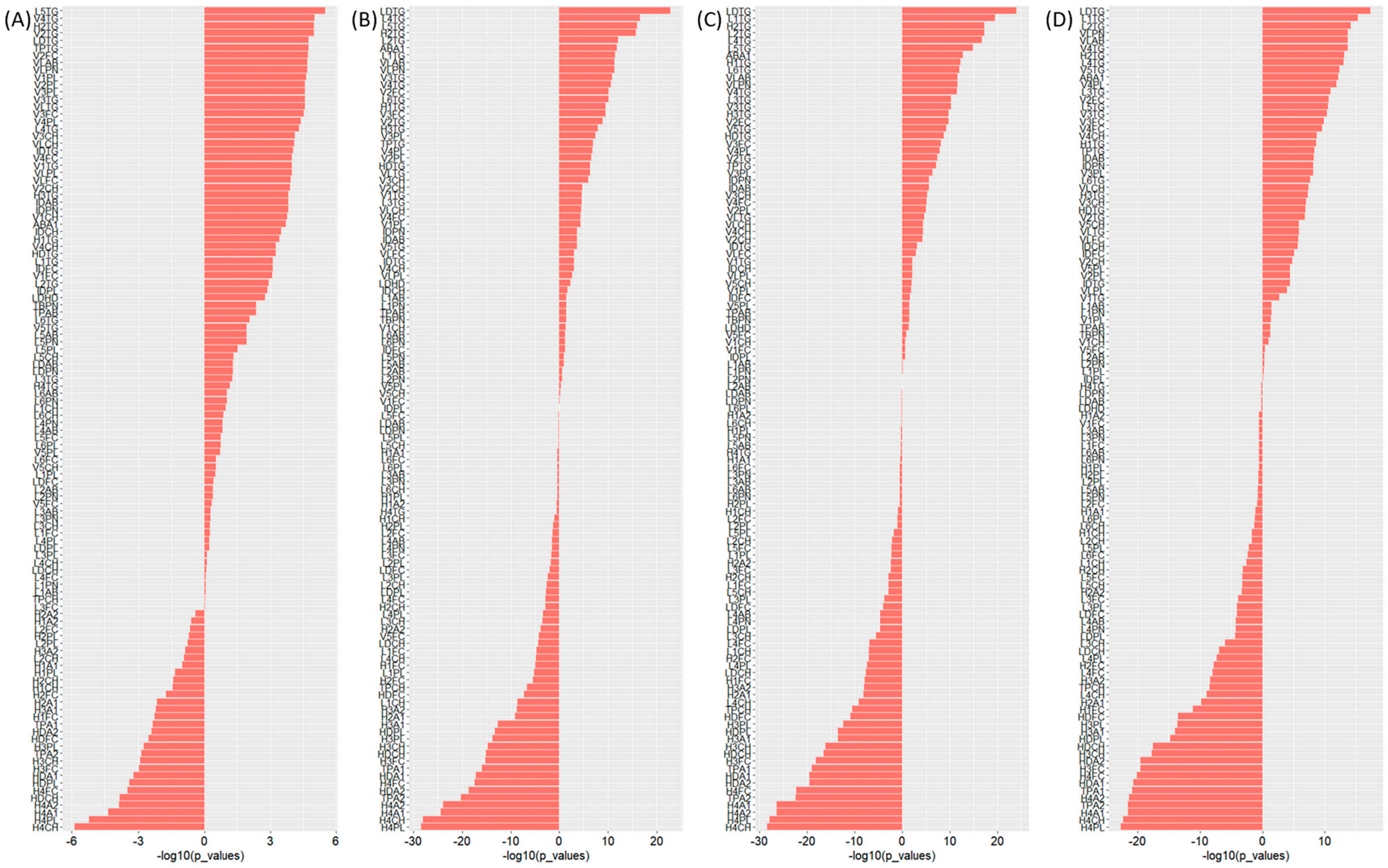
(A)
(B)
(C)
(D)
-log10(p_values)
-6
-3
0
3
6
-30
-20
-10
0
10
20
-30
-20
-10
0
10
20
-20
-10
0
10

Figure S6 - –log 10 (p-values) between controls and SARS-CoV-2 positive patients of each severity group for the lipoproteins. (A) Controls vs group B severity. (B) Controls vs Group C severity. (D) Controls vs Group D severity. (E) Controls vs Group E severity.

Table S10 - OPLS loadings, Cliff's delta and adjusted p-values of the lipoproteins for the comparison of the controls and SARS-CoV-2 positive patients in severity class B. Only metabolites with significant p-values are shown in the table.

Metabolite	OPLS loadings	Cliff's delta	Adjusted p-value
H4CH	0.141	-0.87	$1.57x10^{-4}$
L5TG	0.161	0.84	$1.73x10^{-4}$
TPTG	0.103	0.77	$1.79x10^{-4}$
VLPN	0.133	0.77	$1.79x10^{-4}$
VLTG	0.096	0.76	$1.79x10^{-4}$
LDTG	0.154	0.77	$1.79x10^{-4}$
VLAB	0.133	0.77	$1.79x10^{-4}$
V2TG	0.109	0.80	$1.79x10^{-4}$
V3TG	0.124	0.76	$1.79x10^{-4}$
V4TG	0.134	0.80	$1.79x10^{-4}$
V2FC	0.120	0.77	$1.79x10^{-4}$
V1PL	0.075	0.76	$1.79x10^{-4}$
V2PL	0.107	0.76	$1.79x10^{-4}$
V3PL	0.124	0.76	$1.79x10^{-4}$
H2TG	0.128	0.80	$1.79x10^{-4}$
H4PL	0.135	-0.82	$1.79x10^{-4}$
V3FC	0.117	0.75	$1.97x10^{-4}$
V4PL	0.133	0.74	$2.58x10^{-4}$
H4A1	0.124	-0.74	$2.58x10^{-4}$
L4TG	0.148	0.73	$2.72x10^{-4}$
V3CH	0.119	0.72	$3.93x10^{-4}$
VLCH	0.106	0.71	$4.17x10^{-4}$
IDTG	0.089	0.71	$4.32x10^{-4}$
VLPL	0.098	0.70	$4.32x10^{-4}$
V1TG	0.074	0.70	$4.32x10^{-4}$
V4FC	0.130	0.70	$4.32x10^{-4}$
VLFC	0.099	0.70	$4.84x10^{-4}$
V2CH	0.112	0.69	$4.91x10^{-4}$
HDCH	0.114	-0.68	$5.09x10^{-4}$

IDPN	0.114	0.68	5.09×10^{-4}
IDAB	0.144	0.68	5.09×10^{-4}
H3TG	0.108	0.68	5.09×10^{-4}
H4A2	0.122	-0.69	5.09×10^{-4}
V1CH	0.073	0.68	5.45×10^{-4}
ABA1	0.157	0.67	6.08×10^{-4}
IDCH	0.132	0.65	9.85×10^{-4}
H4FC	0.117	-0.65	9.85×10^{-4}
HDPL	0.102	-0.64	1.13×10^{-3}
H1TG	0.111	0.64	1.13×10^{-3}
HDTG	0.112	0.62	1.55×10^{-3}
V4CH	0.117	0.62	1.55×10^{-3}
HDA1	0.108	-0.62	1.66×10^{-3}
L1TG	0.130	0.61	1.94×10^{-3}
IDFC	0.127	0.61	1.98×10^{-3}
V1FC	0.070	0.60	2.02×10^{-3}
H3FC	0.106	-0.59	2.69×10^{-3}
L2TG	0.122	0.58	2.87×10^{-3}
H3CH	0.108	-0.58	2.94×10^{-3}
TPA2	0.107	-0.58	3.07×10^{-3}
IDPL	0.103	0.58	3.07×10^{-3}
LDHD	0.141	0.56	3.81×10^{-3}
H3PL	0.090	-0.56	3.81×10^{-3}
HDFC	0.102	-0.54	6.36×10^{-3}
HDA2	0.096	-0.52	8.24×10^{-3}
TPAB	0.131	0.52	8.59×10^{-3}
TBPN	0.131	0.52	8.59×10^{-3}
TPA1	0.100	-0.51	9.12×10^{-3}
H1FC	0.084	-0.50	1.04×10^{-2}
H3A1	0.091	-0.49	1.19×10^{-2}
H2A1	0.083	-0.48	1.36×10^{-2}
L6TG	0.121	0.47	1.60×10^{-2}
L5PN	0.099	0.45	2.11×10^{-2}
V5TG	0.079	0.45	2.11×10^{-2}
L5AB	0.099	0.45	2.11×10^{-2}
H2FC	0.090	-0.42	3.22×10^{-2}

Table S11 - OPLS loadings, Cliff's delta and adjusted p-values of the lipoproteins for the comparison of the controls and SARS-CoV-2 positive patients in severity class C. Only metabolites with significant p-values are shown in the table.

Metabolite	OPLS loadings	Cliff's delta	Adjusted p-value
H4CH	0.200	-0.87	$4.77x10^{-27}$
H4PL	0.195	-0.88	$4.77x10^{-27}$
H4A1	0.195	-0.81	$1.72x10^{-23}$
H4A2	0.193	-0.81	$3.19x10^{-23}$
LDTG	0.156	0.79	$3.29x10^{-23}$
TPA2	0.193	-0.74	$1.24x10^{-19}$
HDA2	0.188	-0.71	$3.22x10^{-18}$
H4FC	0.192	-0.68	$4.81x10^{-17}$
HDA1	0.178	-0.68	$8.30x10^{-17}$
L4TG	0.135	0.66	$3.07x10^{-16}$
TPA1	0.178	-0.65	$1.05x10^{-15}$
L5TG	0.131	0.65	$1.05x10^{-15}$
H2TG	0.107	0.65	$1.52x10^{-15}$
H3FC	0.183	-0.64	$4.79x10^{-15}$
HDCH	0.159	-0.63	$5.62x10^{-15}$
H3CH	0.178	-0.62	$1.44x10^{-14}$
H3PL	0.159	-0.60	$1.15x10^{-13}$
HDPL	0.148	-0.59	$3.56x10^{-13}$
H3A1	0.169	-0.58	$1.36x10^{-12}$
L2TG	0.139	0.56	$4.71x10^{-12}$
ABA1	0.135	0.56	$7.45x10^{-12}$
L1TG	0.131	0.55	$1.83x10^{-11}$
VLPN	0.108	0.54	$2.31x10^{-11}$
VLAB	0.108	0.54	$2.31x10^{-11}$
V3TG	0.090	0.53	$6.74x10^{-11}$
V4TG	0.099	0.52	$1.29x10^{-10}$
V2FC	0.091	0.51	$3.20x10^{-10}$
L6TG	0.105	0.51	$3.64x10^{-10}$
V3FC	0.089	0.49	$1.25x10^{-9}$
H1TG	0.088	0.49	$1.25x10^{-9}$
H2A1	0.130	-0.48	$2.77x10^{-9}$
V2TG	0.071	0.47	$5.31x10^{-9}$
H3A2	0.141	-0.47	$6.35x10^{-9}$

L1CH	0.067	-0.47	$6.99x10^{-9}$
H3TG	0.063	0.45	$3.72x10^{-8}$
V3PL	0.085	0.43	$1.29x10^{-7}$
HDFC	0.132	-0.43	$1.42x10^{-7}$
TPTG	0.060	0.42	$3.60x10^{-7}$
V4PL	0.095	0.41	$4.48x10^{-7}$
TPCH	0.109	-0.41	$5.74x10^{-7}$
V2PL	0.066	0.40	$8.18x10^{-7}$
HDTG	0.060	0.40	$1.26x10^{-6}$
VLTG	0.049	0.39	$1.39x10^{-6}$
V3CH	0.078	0.38	$2.77x10^{-6}$
H2FC	0.114	-0.36	$8.54x10^{-6}$
L1PL	0.037	-0.35	$1.71x10^{-5}$
H1FC	0.096	-0.34	$2.75x10^{-5}$
L4CH	0.103	-0.34	$3.61x10^{-5}$
V2CH	0.069	0.33	$4.82x10^{-5}$
L1FC	0.044	-0.33	$4.85x10^{-5}$
V1TG	0.025	0.33	$5.22x10^{-5}$
L3TG	0.094	0.33	$6.05x10^{-5}$
VLCH	0.066	0.33	$6.44x10^{-5}$
V4FC	0.079	0.32	$8.42x10^{-5}$
LDCH	0.083	-0.32	$9.33x10^{-5}$
V1PL	0.031	0.32	$9.91x10^{-5}$
V5FC	0.024	-0.31	$1.24x10^{-4}$
H2A2	0.085	-0.30	$2.41x10^{-4}$
IDPN	0.083	0.29	$5.12x10^{-4}$
IDAB	0.083	0.29	$5.13x10^{-4}$
V5TG	0.050	0.29	$5.13x10^{-4}$
L3CH	0.076	-0.28	$5.40x10^{-4}$
L4PL	0.093	-0.28	$7.32x10^{-4}$
VLFC	0.046	0.26	$1.56x10^{-3}$
IDTG	0.031	0.26	$1.67x10^{-3}$
V4CH	0.073	0.26	$1.85x10^{-3}$
H2CH	0.088	-0.25	$2.02x10^{-3}$
L4FC	0.098	-0.25	$2.38x10^{-3}$
LDPL	0.064	-0.25	$2.58x10^{-3}$
L2CH	0.030	-0.24	$3.33x10^{-3}$

VLPL	0.045	0.24	$3.70x10^{-3}$
L3PL	0.062	-0.22	$7.06x10^{-3}$
LDHD	0.063	0.22	$7.59x10^{-3}$
LDFC	0.059	-0.21	$1.30x10^{-2}$
L2PL	0.017	-0.19	$2.40x10^{-2}$
L3FC	0.063	-0.18	$3.35x10^{-2}$
IDCH	0.052	0.18	$3.37x10^{-2}$
L4PN	0.067	-0.17	$3.75x10^{-2}$
L4AB	0.066	-0.17	$3.75x10^{-2}$
L1PN	0.007	-0.17	$4.79x10^{-2}$
L1AB	0.007	-0.17	$4.79x10^{-2}$

Table S12 - OPLS loadings, Cliff's delta and adjusted p-values of the lipoproteins for the comparison of the controls and SARS-CoV-2 positive patients in severity class D. Only metabolites with significant p-values are shown in the table.

Metabolite	OPLS loadings	Cliff's delta	Adjusted p-value
H4CH	0.191	-0.96	$6.16x10^{-27}$
H4PL	0.189	-0.95	$8.95x10^{-27}$
H4A1	0.191	-0.93	$1.44x10^{-25}$
H4A2	0.186	-0.93	$1.44x10^{-25}$
LDTG	0.143	0.88	$2.23x10^{-23}$
TPA2	0.179	-0.85	$8.89x10^{-22}$
H4FC	0.190	-0.85	$1.05x10^{-21}$
HDA1	0.173	-0.79	$3.96x10^{-19}$
HDA2	0.169	-0.79	$3.96x10^{-19}$
L1TG	0.131	0.79	$3.96x10^{-19}$
TPA1	0.175	-0.78	$1.02x10^{-18}$
H3FC	0.178	-0.76	$7.79x10^{-18}$
H2TG	0.126	0.74	$4.40x10^{-17}$
L2TG	0.132	0.74	$4.46x10^{-17}$
L4TG	0.121	0.73	$1.53x10^{-16}$
HDCH	0.151	-0.73	$2.40x10^{-16}$
H3CH	0.162	-0.72	$5.26x10^{-16}$
L5TG	0.117	0.69	$7.42x10^{-15}$
H3A1	0.154	-0.66	$1.83x10^{-13}$

HDPL	0.140	-0.65	$1.94x10^{-13}$
ABA1	0.126	0.63	$9.32x10^{-13}$
H3PL	0.139	-0.62	$2.01x10^{-12}$
H1TG	0.111	0.62	$3.20x10^{-12}$
L6TG	0.106	0.61	$5.25x10^{-12}$
VLPN	0.103	0.60	$1.01x10^{-11}$
VLAB	0.103	0.60	$1.01x10^{-11}$
V4TG	0.096	0.60	$1.19x10^{-11}$
HDFC	0.140	-0.58	$5.71x10^{-11}$
TPCH	0.125	-0.57	$1.28x10^{-10}$
L3TG	0.101	0.57	$1.92x10^{-10}$
V3TG	0.086	0.56	$2.03x10^{-10}$
H3TG	0.090	0.55	$5.73x10^{-10}$
V2FC	0.091	0.55	$6.11x10^{-10}$
V5TG	0.083	0.53	$1.72x10^{-9}$
L4CH	0.125	-0.53	$2.58x10^{-9}$
HDTG	0.090	0.52	$5.28x10^{-9}$
V3FC	0.081	0.50	$2.15x10^{-8}$
H2A1	0.115	-0.50	$2.20x10^{-8}$
H3A2	0.114	-0.49	$3.34x10^{-8}$
H1FC	0.106	-0.49	$3.53x10^{-8}$
V4PL	0.091	0.49	$4.21x10^{-8}$
LDCH	0.109	-0.48	$7.00x10^{-8}$
L4PL	0.116	-0.47	$1.09x10^{-7}$
V2TG	0.068	0.47	$1.09x10^{-7}$
TPTG	0.060	0.46	$1.70x10^{-7}$
H2FC	0.112	-0.46	$2.27x10^{-7}$
L1CH	0.044	-0.46	$2.48x10^{-7}$
L4FC	0.123	-0.45	$3.27x10^{-7}$
V3PL	0.078	0.43	$1.03x10^{-6}$
IDPN	0.082	0.41	$5.58x10^{-6}$
IDAB	0.082	0.40	$5.58x10^{-6}$
L3CH	0.091	-0.40	$7.83x10^{-6}$
V3CH	0.071	0.39	$1.15x10^{-5}$
V4FC	0.081	0.38	$1.61x10^{-5}$
V2PL	0.062	0.38	$2.12x10^{-5}$
VLTG	0.048	0.36	$4.51x10^{-5}$

LDPL	0.087	-0.36	4.81×10^{-5}
L4PN	0.092	-0.36	5.16×10^{-5}
L4AB	0.092	-0.36	5.16×10^{-5}
VLCH	0.064	0.35	8.33×10^{-5}
V4CH	0.074	0.35	8.76×10^{-5}
V2CH	0.067	0.35	9.56×10^{-5}
LDFC	0.086	-0.33	2.08×10^{-4}
L3PL	0.075	-0.32	3.46×10^{-4}
IDTG	0.038	0.29	1.27×10^{-3}
VLFC	0.049	0.28	2.00×10^{-3}
L5CH	0.083	-0.28	2.05×10^{-3}
L1FC	0.025	-0.28	2.27×10^{-3}
H2CH	0.075	-0.27	2.56×10^{-3}
L3FC	0.073	-0.25	6.16×10^{-3}
H2A2	0.065	-0.25	6.30×10^{-3}
L1PL	0.005	-0.24	8.14×10^{-3}
L5FC	0.088	-0.24	8.65×10^{-3}
V1TG	0.029	0.23	1.00×10^{-2}
L2CH	0.031	-0.23	1.16×10^{-2}
IDCH	0.054	0.23	1.17×10^{-2}
VLPL	0.044	0.23	1.21×10^{-2}
V5CH	0.044	0.22	1.40×10^{-2}
V1PL	0.031	0.21	1.82×10^{-2}
L5PL	0.070	-0.20	2.61×10^{-2}
IDFC	0.048	0.19	3.52×10^{-2}
V5PL	0.030	0.19	4.21×10^{-2}
TPAB	0.016	0.18	4.73×10^{-2}
TBPN	0.016	0.18	4.73×10^{-2}

Table S13 - OPLS loadings, Cliff's delta and adjusted p-values of the lipoproteins for the comparison of the controls and SARS-CoV-2 positive patients in severity class E. Only metabolites with significant p-values are shown in the table.

Metabolite	OPLS loadings	Cliff's delta	Adjusted p-value
H4PL	0.165	-0.98	2.70×10^{-21}
H4CH	0.166	-0.97	3.01×10^{-21}
TPA2	0.158	-0.95	7.83×10^{-21}

H4A1	0.167	-0.95	$7.83x10^{-21}$
H4A2	0.161	-0.95	$1.02x10^{-20}$
TPA1	0.160	-0.94	$2.39x10^{-20}$
HDA1	0.158	-0.93	$3.32x10^{-20}$
H4FC	0.166	-0.92	$1.06x10^{-19}$
HDA2	0.150	-0.91	$3.57x10^{-19}$
H3FC	0.163	-0.91	$3.57x10^{-19}$
H3CH	0.148	-0.86	$2.11x10^{-17}$
HDCH	0.140	-0.86	$3.02x10^{-17}$
LDTG	0.144	0.85	$4.11x10^{-17}$
L1TG	0.145	0.80	$4.36x10^{-15}$
HDPL	0.129	-0.78	$1.36x10^{-14}$
L2TG	0.142	0.76	$5.23x10^{-14}$
H3A1	0.134	-0.76	$6.97x10^{-14}$
VLPN	0.124	0.75	$1.16x10^{-13}$
VLAB	0.124	0.75	$1.16x10^{-13}$
V4TG	0.121	0.75	$1.16x10^{-13}$
H3PL	0.124	-0.75	$1.31x10^{-13}$
HDFC	0.138	-0.75	$1.47x10^{-13}$
H2TG	0.119	0.74	$3.52x10^{-13}$
L4TG	0.127	0.73	$4.65x10^{-13}$
V5TG	0.116	0.71	$2.18x10^{-12}$
ABA1	0.120	0.70	$3.23x10^{-12}$
V4PL	0.120	0.70	$6.23x10^{-12}$
H1FC	0.112	-0.67	$2.92x10^{-11}$
L3TG	0.121	0.67	$4.92x10^{-11}$
V2FC	0.098	0.66	$8.04x10^{-11}$
L5TG	0.107	0.65	$9.96x10^{-11}$
V3TG	0.096	0.65	$1.55x10^{-10}$
V3FC	0.099	0.63	$5.03x10^{-10}$
H2A1	0.102	-0.63	$5.03x10^{-10}$
V4FC	0.115	0.62	$7.93x10^{-10}$
L4CH	0.110	-0.60	$3.78x10^{-9}$
V4CH	0.111	0.59	$6.08x10^{-9}$
H1TG	0.108	0.59	$7.39x10^{-9}$
TPCH	0.110	-0.58	$9.54x10^{-9}$
TPTG	0.088	0.58	$1.34x10^{-8}$

H3A2	0.099	-0.58	$1.34x10^{-8}$
IDAB	0.113	0.57	$1.70x10^{-8}$
IDPN	0.113	0.57	$1.74x10^{-8}$
V3PL	0.091	0.57	$2.04x10^{-8}$
L4FC	0.114	-0.56	$2.94x10^{-8}$
H2FC	0.102	-0.55	$4.38x10^{-8}$
L6TG	0.091	0.55	$5.66x10^{-8}$
VLCH	0.090	0.54	$9.94x10^{-8}$
L4PL	0.101	-0.54	$1.10x10^{-7}$
H3TG	0.085	0.54	$1.10x10^{-7}$
V3CH	0.085	0.52	$2.06x10^{-7}$
LDCH	0.103	-0.52	$2.59x10^{-7}$
HDTG	0.094	0.52	$2.60x10^{-7}$
V2TG	0.068	0.52	$3.31x10^{-7}$
L3CH	0.087	-0.48	$2.18x10^{-6}$
V5CH	0.097	0.48	$2.62x10^{-6}$
VLTG	0.063	0.47	$2.97x10^{-6}$
VLFC	0.075	0.47	$3.75x10^{-6}$
IDCH	0.094	0.47	$3.91x10^{-6}$
IDFC	0.089	0.44	$1.55x10^{-5}$
V2CH	0.063	0.42	$2.86x10^{-5}$
V2PL	0.055	0.41	$6.40x10^{-5}$
V5PL	0.083	0.41	$6.40x10^{-5}$
IDTG	0.050	0.40	$7.17x10^{-5}$
IDPL	0.083	-0.40	$7.19x10^{-5}$
L4PN	0.077	-0.40	$9.02x10^{-5}$
L4AB	0.077	-0.40	$9.02x10^{-5}$
LDFC	0.083	-0.39	$1.37x10^{-4}$
L3PL	0.072	-0.39	$1.41x10^{-4}$
VLPL	0.061	0.38	$1.69x10^{-4}$
L3FC	0.080	-0.37	$2.18x10^{-4}$
H2A2	0.051	-0.34	$7.86x10^{-4}$
L5CH	0.075	-0.34	$9.84x10^{-4}$
L5FC	0.085	-0.33	$1.02x10^{-3}$
H2CH	0.064	-0.33	$1.21x10^{-3}$
V1TG	0.029	0.30	$3.04x10^{-3}$
L1CH	0.026	-0.30	$3.84x10^{-3}$

L6FC	0.078	-0.28	$6.97x10^{-3}$
L5PL	0.069	-0.26	$1.10x10^{-2}$
L2CH	0.037	-0.23	$2.76x10^{-2}$
H1CH	0.045	-0.23	$2.76x10^{-2}$
L1PN	0.066	0.21	$4.32x10^{-2}$
L1AB	0.066	0.21	$4.32x10^{-2}$

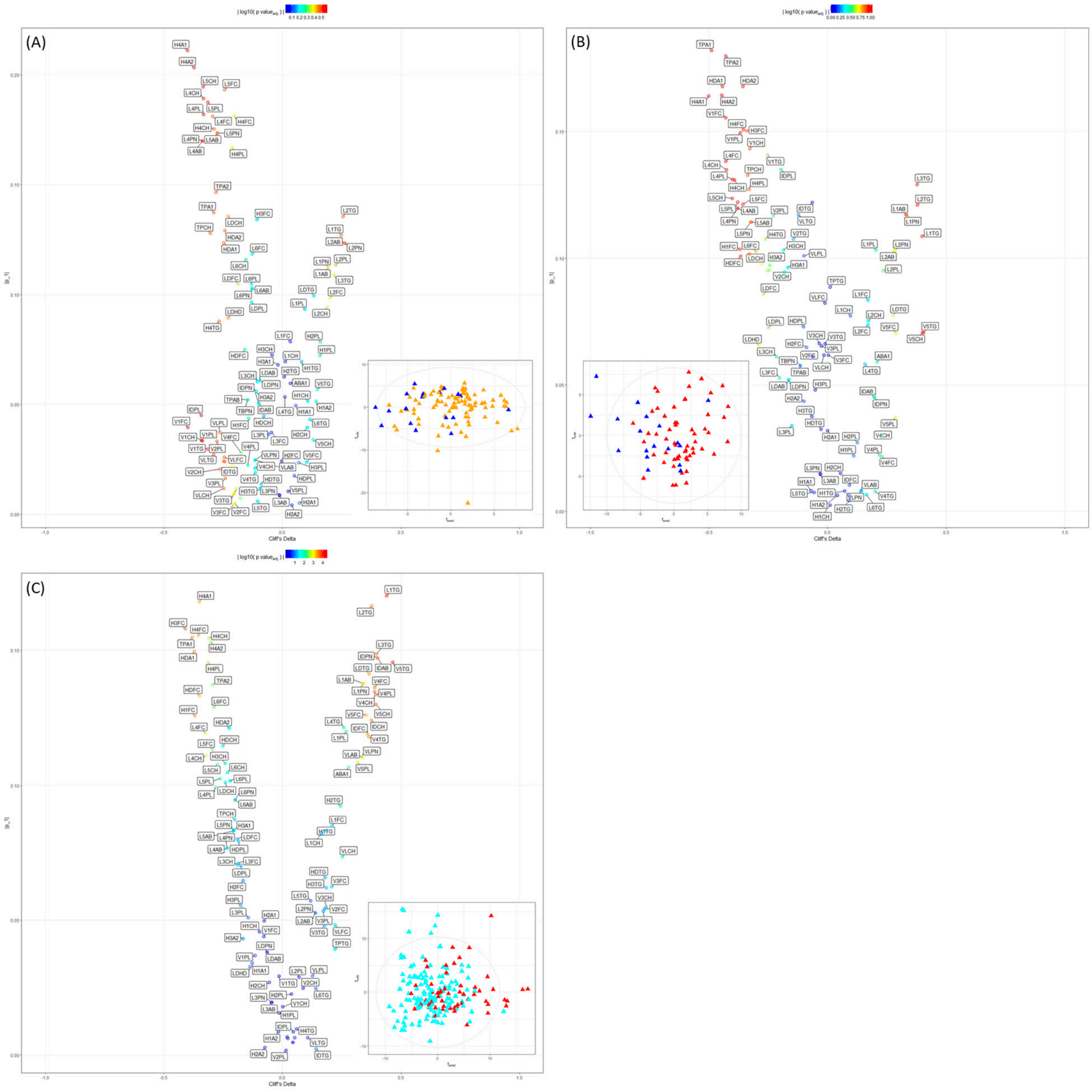

Figure S8 - Orthogonal Partial Least Squared (O-PLS-DA) and eruption plots between different severity classes of SARS-CoV-2 positive patients for the lipoproteins. (A) O-PLS-DA SARS-CoV-2 severity group B (blue) and SARS-CoV-2 severity group D (orange) (R_2X=0.18, AUROC=0.64) and eruption plot. (B) O-PLS-DA SARS-CoV-2 severity group B

(blue) and SARS-CoV-2 severity group E (red) (R_2X=0.21, AUROC=0.70) and eruption plot. (C) O-PLS-DA SARS-CoV-2 severity group C (cyan) and SARS-CoV-2 severity group E (red) (R_2X=0.28, AUROC=0.72) and eruption plot. Significant models could not be produced for severity group B vs C, group C vs D or D vs E.

Table S14 - OPLS loadings, Cliff's delta and adjusted p-values of the lipoproteins for the comparison of severity class C and E. Only metabolites with significant p-values are shown in the table.

Metabolite	OPLS loadings	Cliff's delta	Adjusted p-value
V5TG	0.146	0.47	$3.50x10^{-5}$
L1TG	0.170	0.44	$7.69x10^{-5}$
IDPN	0.147	0.40	$2.00x10^{-4}$
IDAB	0.147	0.40	$2.00x10^{-4}$
V5CH	0.130	0.40	$2.00x10^{-4}$
V4PL	0.134	0.40	$2.00x10^{-4}$
L3TG	0.149	0.40	$2.00x10^{-4}$
H3FC	0.158	-0.41	$2.00x10^{-4}$
V4CH	0.135	0.39	$2.20x10^{-4}$
V4FC	0.136	0.39	$2.20x10^{-4}$
TPA1	0.155	-0.38	$3.08x10^{-4}$
L2TG	0.166	0.38	$3.26x10^{-4}$
IDCH	0.124	0.37	$3.40x10^{-4}$
HDA1	0.150	-0.37	$3.58x10^{-4}$
H1FC	0.126	-0.37	$3.65x10^{-4}$
LDTG	0.141	0.37	$4.20x10^{-4}$
V4TG	0.118	0.36	$4.20x10^{-4}$
V5FC	0.126	0.35	$4.57x10^{-4}$
IDFC	0.119	0.36	$4.58x10^{-4}$
H4FC	0.156	-0.35	$5.42x10^{-4}$
H4A1	0.168	-0.35	$6.02x10^{-4}$
HDFC	0.133	-0.35	$6.15x10^{-4}$
L1PN	0.138	0.34	$8.71x10^{-4}$
L1AB	0.138	0.34	$9.64x10^{-4}$
VLPN	0.110	0.34	$9.64x10^{-4}$
VLAB	0.110	0.34	$9.64x10^{-4}$
L4FC	0.119	-0.33	$1.46x10^{-3}$
L4CH	0.111	-0.32	$1.53x10^{-3}$
V5PL	0.109	0.32	$1.91x10^{-3}$
H4PL	0.146	-0.31	$2.08x10^{-3}$
H4CH	0.154	-0.31	$2.39x10^{-3}$

H4A2	0.153	-0.30	$3.07x10^{-3}$
TPA2	0.137	-0.29	$4.04x10^{-3}$
L5FC	0.113	-0.29	$4.04x10^{-3}$
L6FC	0.129	-0.29	$4.70x10^{-3}$
L4PL	0.099	-0.28	$5.81x10^{-3}$
ABA1	0.107	0.28	$6.27x10^{-3}$
L5CH	0.108	-0.27	$7.41x10^{-3}$
L1PL	0.120	0.27	$8.88x10^{-3}$
L5PL	0.103	-0.26	$1.09x10^{-2}$
L4TG	0.121	0.26	$1.26x10^{-2}$
HDCH	0.115	-0.25	$1.49x10^{-2}$
VLCH	0.073	0.25	$1.49x10^{-2}$
H2TG	0.092	0.24	$1.87x10^{-2}$
LDCH	0.101	-0.24	$1.98x10^{-2}$
H3CH	0.108	-0.24	$2.00x10^{-2}$
L4PN	0.077	-0.23	$2.47x10^{-2}$
L4AB	0.077	-0.23	$2.47x10^{-2}$
L6CH	0.105	-0.23	$2.51x10^{-2}$
HDA2	0.121	-0.22	$3.07x10^{-2}$
TPTG	0.039	0.22	$3.14x10^{-2}$
VLFC	0.048	0.22	$3.14x10^{-2}$
L6PL	0.102	-0.22	$3.14x10^{-2}$
L1FC	0.085	0.21	$4.35x10^{-2}$
L5PN	0.083	-0.21	$4.56x10^{-2}$
V3FC	0.062	0.21	$4.56x10^{-2}$
L5AB	0.083	-0.21	$4.56x10^{-2}$
H3A1	0.083	-0.20	$4.81x10^{-2}$

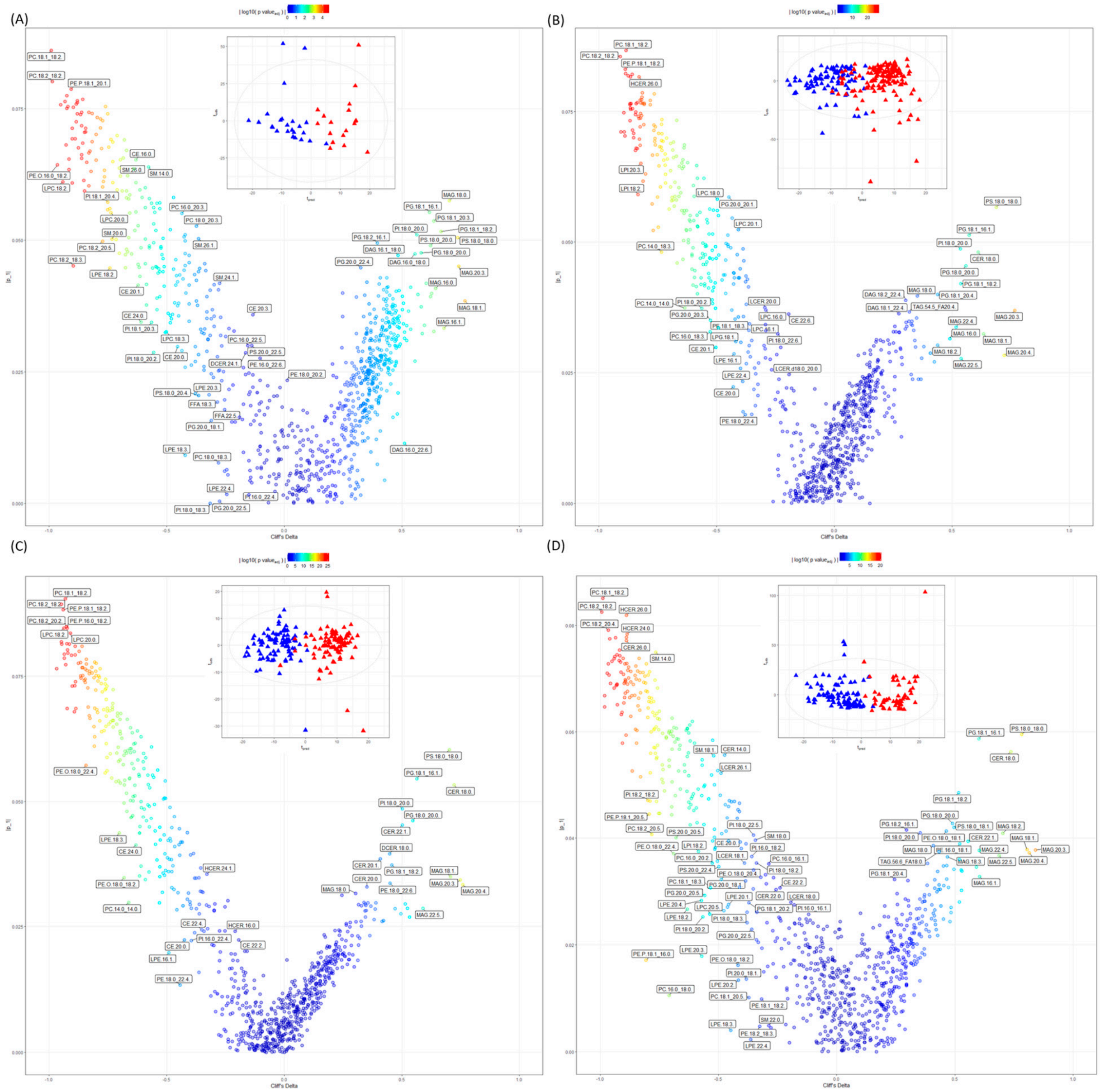

Figure S9 - Orthogonal Partial Least Squared (O-PLS-DA) and eruption plots between controls and SARS-CoV-2 positive patients of each severity group for the Lipids. (A) Eruption plot of the controls vs group B severity group with the O-PLS-DA of controls (blue triangles) and SARS-CoV-2 group B severity patients (red triangles) (R_2X=0.22, AUROC=0.98) inset. (B) Eruption plot of the controls vs group C severity group with the O-PLS-DA of controls (blue triangles) and SARS-CoV-2 group C severity patients (red triangles) (R_2X=0.18, AUROC=0.97) inset. (C) Eruption plot of the controls vs group D severity group with the O-PLS-DA of controls (blue triangles) and SARS-CoV-2 group D severity patients (red triangles) (R_2X=0.12, AUROC=0.99) inset. (D) Eruption plot of the controls vs group E severity group with the O-PLS-DA of controls (blue triangles) and SARS-CoV-2 group E severity patients (red triangles) (R_2X=0.21, AUROC=0.99) inset.

Table S15 - OPLS loadings, Cliff's delta and adjusted p-values of the lipids for the comparison of the controls and SARS-CoV-2 positive patients in severity class B. Only the 50 most significant adjusted p-values are shown.

Metabolite	OPLS loadings	Cliff's delta	Adjusted p-value
PC (18:1/18:2)	0.086	-0.99	$2.00x10^{-5}$
PC (18:2/18:2)	0.080	-0.99	$2.00x10^{-5}$
PE.O (16:0/18:2)	0.064	-0.96	$2.65x10^{-5}$
HCER.d (18:0/18:0)	0.075	-0.92	$2.85x10^{-5}$
LPC (18:2)	0.061	-0.94	$2.85x10^{-5}$
PE.O (16:0/20:4)	0.063	-0.92	$2.85x10^{-5}$
PE.P (16:0/18:2)	0.077	-0.92	$2.85x10^{-5}$
PE.P (16:0/18:3)	0.065	-0.92	$2.85x10^{-5}$
PE.P (18:0/18:2)	0.076	-0.93	$2.85x10^{-5}$
PE.P (18:0/20:1)	0.075	-0.92	$2.85x10^{-5}$
PE.P (18:1/18:2)	0.076	-0.95	$2.85x10^{-5}$
PE.P (18:2/20:4)	0.068	-0.93	$2.85x10^{-5}$
PC (18:2/20:2)	0.061	-0.90	$3.14x10^{-5}$
PE.O (18:0/22:5)	0.071	-0.90	$3.14x10^{-5}$
PE.P (18:1/20:1)	0.079	-0.91	$3.14x10^{-5}$
PE.P (18:1/20:4)	0.069	-0.90	$3.14x10^{-5}$
PC (18:2/18:3)	0.045	-0.90	$3.17x10^{-5}$
PC (18:2/20:4)	0.075	-0.90	$3.17x10^{-5}$
HCER (22:0)	0.069	-0.89	$3.87x10^{-5}$
PC (16:0/18:2)	0.076	-0.88	$3.93x10^{-5}$
PC (20:0/20:4)	0.074	-0.88	$3.93x10^{-5}$
PE.O (16:0/20:1)	0.073	-0.88	$3.93x10^{-5}$
PE.P (18:0/18:1)	0.077	-0.88	$3.93x10^{-5}$
PE.P (18:1/22:5)	0.076	-0.88	$3.93x10^{-5}$
PE.O (18:0/22:4)	0.068	-0.88	$4.28x10^{-5}$
PC (18:0/20:1)	0.074	-0.87	$5.16x10^{-5}$
PE.O (16:0/18:1)	0.066	-0.86	$5.16x10^{-5}$
PE.P (16:0/22:5)	0.074	-0.86	$5.16x10^{-5}$
PE.P (18:0/20:4)	0.066	-0.86	$5.16x10^{-5}$
PE.P (18:1/22:4)	0.072	-0.86	$5.16x10^{-5}$
PE.P (18:0/22:5)	0.068	-0.86	$5.46x10^{-5}$
PE.P (18:2/18:2)	0.068	-0.86	$5.46x10^{-5}$
PE.O (16:0/22:5)	0.061	-0.85	$5.98x10^{-5}$

PE.O (16:0/22:6)	0.059	-0.85	$6.36x10^{-5}$
PE.P (18:0/22:4)	0.071	-0.85	$6.36x10^{-5}$
PC (20:0/18:1)	0.071	-0.84	$6.79x10^{-5}$
PE.P (18:0/18:0)	0.071	-0.84	$6.79x10^{-5}$
PE.P (16:0/20:4)	0.065	-0.84	$7.45x10^{-5}$
HCER.d (18:0/22:0)	0.067	-0.83	$8.76x10^{-5}$
PE.P (16:0/20:1)	0.074	-0.83	$8.76x10^{-5}$
PE.P (18:1/16:0)	0.060	-0.83	$8.76x10^{-5}$
PE.P (18:0/16:0)	0.067	-0.83	$9.62x10^{-5}$
PC (20:0/20:3)	0.063	-0.82	$1.06x10^{-4}$
PC (18:2/20:3)	0.069	-0.82	$1.14x10^{-4}$
PE.P (18:1/18:1)	0.074	-0.82	$1.14x10^{-4}$
PC (18:2/22:6)	0.065	-0.80	$1.72x10^{-4}$
PE.P (18:0/20:2)	0.070	-0.80	$1.72x10^{-4}$
PC (16:0/20:1)	0.073	-0.80	$1.89x10^{-4}$
HCER.d (18:0/24:0)	0.073	-0.78	$2.55x10^{-4}$
PE.P (16:0/18:1)	0.071	-0.78	$2.55x10^{-4}$

Table S16 - OPLS loadings, Cliff's delta and adjusted p-values of the lipids for the comparison of the controls and SARS-CoV-2 positive patients in severity class C. Only the 50 most significant adjusted p-values are shown.

**Metabolite**	**OPLS loadings**	**Cliff's delta**	**Adjusted p-value**
PC (18:2/18:2)	0.086	-0.91	$3.47x10^{-28}$
PE.O (16:0/20:4)	0.071	-0.91	$3.47x10^{-28}$
PE.O (16:0/18:2)	0.075	-0.89	$1.24x10^{-27}$
PE.P (18:2/20:4)	0.071	-0.89	$1.24x10^{-27}$
PE.P (16:0/22:5)	0.070	-0.89	$1.75x10^{-27}$
PE.P (16:0/18:2)	0.082	-0.89	$1.78x10^{-27}$
PE.P (18:1/18:2)	0.083	-0.89	$1.78x10^{-27}$
PC (18:1/18:2)	0.087	-0.88	$2.16x10^{-27}$
PE.P (18:0/18:2)	0.082	-0.88	$6.43x10^{-27}$
HCER.d (18:0/18:0)	0.076	-0.87	$1.56x10^{-26}$
PE.O (16:0/18:1)	0.073	-0.87	$1.56x10^{-26}$
PE.O (16:0/20:1)	0.076	-0.87	$1.56x10^{-26}$
PE.P (18:0/18:1)	0.082	-0.87	$1.76x10^{-26}$

PE.P (18:0/22:5)	0.076	-0.87	$1.76x10^{-26}$
PE.P (18:1/20:4)	0.075	-0.87	$1.80x10^{-26}$
PC (18:2/20:2)	0.076	-0.86	$2.43x10^{-26}$
PE.P (16:0/20:1)	0.082	-0.86	$4.43x10^{-26}$
LPC (18:2)	0.076	-0.86	$4.58x10^{-26}$
PC (18:2/20:3)	0.071	-0.86	$4.64x10^{-26}$
PE.O (18:0/22:5)	0.070	-0.85	$8.09x10^{-26}$
PE.P (16:0/20:4)	0.071	-0.85	$8.09x10^{-26}$
PC (20:0/20:3)	0.074	-0.84	$2.26x10^{-25}$
PE.P (18:0/22:4)	0.068	-0.84	$2.26x10^{-25}$
PE.P (18:2/18:2)	0.075	-0.84	$2.65x10^{-25}$
PC (18:0/20:1)	0.076	-0.84	$2.78x10^{-25}$
PE.O (18:0/22:4)	0.068	-0.84	$2.80x10^{-25}$
PE.P (18:0/20:1)	0.077	-0.84	$4.80x10^{-25}$
LPI (18:1)	0.068	-0.84	$4.94x10^{-25}$
LPI (18:2)	0.059	-0.83	$6.95x10^{-25}$
PE.P (18:1/22:5)	0.074	-0.83	$9.14x10^{-25}$
HCER (26:0)	0.082	-0.83	$1.15x10^{-24}$
PE.P (18:0/20:3)	0.074	-0.83	$1.19x10^{-24}$
PE.P (16:0/18:3)	0.070	-0.83	$1.35x10^{-24}$
PE.P (18:0/20:4)	0.072	-0.83	$1.94x10^{-24}$
PE.O (16:0/22:5)	0.069	-0.82	$2.09x10^{-24}$
PE.P (18:0/20:2)	0.077	-0.82	$2.09x10^{-24}$
HCER (22:0)	0.075	-0.82	$2.46x10^{-24}$
HCER.d (18:0/22:0)	0.074	-0.82	$2.46x10^{-24}$
LPI (20:3)	0.065	-0.82	$2.55x10^{-24}$
PE.P (18:0/18:0)	0.077	-0.82	$2.55x10^{-24}$
PC (20:0/18:1)	0.074	-0.82	$4.27x10^{-24}$
PE.P (18:1/18:1)	0.077	-0.82	$4.36x10^{-24}$
PC (18:2/20:4)	0.078	-0.82	$4.95x10^{-24}$
LPC (20:0)	0.079	-0.82	$5.75x10^{-24}$
HCER.d (18:0/24:0)	0.077	-0.81	$7.44x10^{-24}$
PE.P (18:1/22:4)	0.062	-0.81	$8.83x10^{-24}$
PI (18:1/18:1)	0.072	-0.81	$1.22x10^{-23}$
PE.P (18:0/16:0)	0.076	-0.81	$1.33x10^{-23}$
HCER (24:0)	0.077	-0.81	$1.48x10^{-23}$
PC (18:2/18:3)	0.063	-0.80	$3.23x10^{-23}$

Table S17 - OPLS loadings, Cliff's delta and adjusted p-values of the lipids for the comparison of the controls and SARS-CoV-2 positive patients in severity class D. Only the 50 most significant adjusted p-values are shown.

Metabolite	OPLS loadings	Cliff's delta	Adjusted p-value
PE.O (16:0/20:4)	0.077	-0.97	$1.91x10^{-26}$
PC (18:2/18:2)	0.089	-0.95	$8.63x10^{-26}$
PE.P (18:1/20:4)	0.082	-0.95	$8.63x10^{-26}$
PE.P (18:2/20:4)	0.081	-0.95	$8.63x10^{-26}$
PE.P (18:1/18:2)	0.088	-0.94	$1.56x10^{-25}$
LPC (18:2)	0.085	-0.94	$1.76x10^{-25}$
PE.P (16:0/20:4)	0.079	-0.94	$1.76x10^{-25}$
PE.P (16:0/22:5)	0.079	-0.94	$1.76x10^{-25}$
PC (18:1/18:2)	0.090	-0.93	$2.91x10^{-25}$
PE.O (16:0/18:2)	0.077	-0.93	$2.97x10^{-25}$
PC (18:1/20:2)	0.085	-0.93	$3.67x10^{-25}$
PE.P (18:0/18:2)	0.081	-0.92	$4.58x10^{-25}$
PC (18:2/18:3)	0.069	-0.92	$4.78x10^{-25}$
PE.P (16:0/18:2)	0.085	-0.92	$4.98x10^{-25}$
PE.P (18:0/22:5)	0.080	-0.92	$4.98x10^{-25}$
PC (18:2/20:3)	0.080	-0.92	$5.97x10^{-25}$
PE.P (18:2/18:2)	0.081	-0.91	$1.28x10^{-24}$
LPC (20:0)	0.084	-0.91	$1.85x10^{-24}$
PE.P (18:1/22:5)	0.079	-0.91	$3.30x10^{-24}$
PE.O (16:0/22:5)	0.073	-0.90	$4.92x10^{-24}$
PE.O (16:0/18:1)	0.068	-0.89	$1.46x10^{-23}$
PE.O (18:0/22:5)	0.073	-0.89	$1.71x10^{-23}$
PE.P (18:0/20:4)	0.079	-0.89	$1.84x10^{-23}$
PE.P (18:0/18:1)	0.081	-0.89	$1.87x10^{-23}$
PC (20:0/20:3)	0.074	-0.89	$2.02x10^{-23}$
PE.P (16:0/18:3)	0.069	-0.89	$2.54x10^{-23}$
PC (18:2/20:4)	0.078	-0.88	$8.13x10^{-23}$
HCER.d (18:0/18:0)	0.080	-0.87	$1.08x10^{-22}$
PE.O (16:0/20:1)	0.075	-0.87	$2.15x10^{-22}$
PI (20:0/18:2)	0.078	-0.87	$2.33x10^{-22}$
PE.P (18:1/22:4)	0.075	-0.87	$2.46x10^{-22}$
LPC (20:3)	0.077	-0.86	$4.35x10^{-22}$
PE.P (16:0/20:1)	0.081	-0.86	$4.35x10^{-22}$

PE.P (18:0/16:0)	0.077	-0.86	$5.62x10^{-22}$
PE.P (18:0/22:4)	0.075	-0.86	$5.95x10^{-22}$
PI (18:1/18:1)	0.074	-0.85	$1.11x10^{-21}$
PE.P (18:0/20:3)	0.074	-0.85	$1.70x10^{-21}$
SM (24:0)	0.076	-0.85	$1.70x10^{-21}$
PC (18:0/20:1)	0.074	-0.85	$1.91x10^{-21}$
PE.O (18:0/22:4)	0.057	-0.84	$2.61x10^{-21}$
PE.P (18:1/18:1)	0.078	-0.84	$2.77x10^{-21}$
PE.P (18:0/20:1)	0.077	-0.84	$3.03x10^{-21}$
PE.P (18:0/18:0)	0.076	-0.84	$4.02x10^{-21}$
PE.P (16:0/22:4)	0.073	-0.84	$5.05x10^{-21}$
LPI (20:3)	0.069	-0.83	$6.35x10^{-21}$
PE.P (18:0/20:2)	0.075	-0.83	$1.05x10^{-20}$
LPC (18:3)	0.067	-0.83	$1.09x10^{-20}$
LPC (18:1)	0.076	-0.83	$1.16x10^{-20}$
SM (20:0)	0.067	-0.83	$1.34x10^{-20}$
PC (18:2/22:6)	0.077	-0.82	$3.24x10^{-20}$

Table S18 - OPLS loadings, Cliff's delta and adjusted p-values of the lipids for the comparison of the controls and SARS-CoV-2 positive patients in severity class E. Only the 50 most significant adjusted p values are shown.

Metabolite	OPLS loadings	Cliff's delta	Adjusted p-value
PC (18:1/18:2)	0.085	-0.99	$3.35x10^{-21}$
PC (18:2/18:2)	0.082	-0.99	$3.35x10^{-21}$
PE.O (16:0/20:4)	0.065	-0.98	$4.10x10^{-21}$
PE.P (18:2/20:4)	0.067	-0.98	$4.10x10^{-21}$
PC (20:0/20:3)	0.071	-0.97	$9.70x10^{-21}$
PC (18:2/20:3)	0.073	-0.97	$9.93x10^{-21}$
PC (18:2/20:4)	0.079	-0.97	$9.93x10^{-21}$
PE.P (18:1/20:4)	0.071	-0.96	$1.19x10^{-20}$
PE.P (16:0/18:2)	0.077	-0.96	$1.52x10^{-20}$
LPC (18:2)	0.069	-0.96	$2.03x10^{-20}$
PE.P (16:0/22:5)	0.073	-0.95	$2.95x10^{-20}$
PE.P (18:1/18:2)	0.074	-0.94	$6.91x10^{-20}$
PE.P (16:0/20:4)	0.068	-0.94	$7.16x10^{-20}$

PE.P (18:0/22:5)	0.074	-0.94	$8.07x10^{-20}$
PC (18:0/20:1)	0.078	-0.94	$1.03x10^{-19}$
PE.P (18:2/18:2)	0.069	-0.93	$1.41x10^{-19}$
PE.P (18:0/20:3)	0.069	-0.93	$1.58x10^{-19}$
PE.P (18:0/20:4)	0.069	-0.93	$1.58x10^{-19}$
PE.P (18:0/18:2)	0.074	-0.93	$1.62x10^{-19}$
PE.O (16:0/22:5)	0.065	-0.93	$1.73x10^{-19}$
HCER (22:0)	0.075	-0.92	$3.49x10^{-19}$
PC (18:2/22:5)	0.073	-0.92	$3.49x10^{-19}$
HCER.d (18:0/22:0)	0.074	-0.92	$4.19x10^{-19}$
LPC (20:0)	0.073	-0.91	$5.04x10^{-19}$
PE.P (18:0/22:4)	0.071	-0.91	$5.84x10^{-19}$
PI (20:0/18:2)	0.063	-0.91	$6.05x10^{-19}$
PE.O (16:0/18:2)	0.064	-0.91	$8.49x10^{-19}$
PE.P (16:0/18:3)	0.058	-0.91	$8.82x10^{-19}$
PE.P (16:0/20:1)	0.074	-0.90	$1.28x10^{-18}$
PC (18:2/22:6)	0.071	-0.90	$1.39x10^{-18}$
PC (18:2/20:2)	0.065	-0.90	$1.45x10^{-18}$
PE.O (16:0/20:1)	0.069	-0.90	$1.75x10^{-18}$
PE.P (18:1/22:4)	0.066	-0.90	$1.90x10^{-18}$
PE.P (18:0/16:0)	0.073	-0.90	$2.14x10^{-18}$
PE.P (18:1/22:5)	0.069	-0.89	$2.15x10^{-18}$
PE.P (16:0/22:4)	0.067	-0.89	$2.17x10^{-18}$
PE.P (18:0/20:1)	0.070	-0.89	$2.19x10^{-18}$
HCER.d (18:0/18:0)	0.070	-0.89	$2.47x10^{-18}$
SM (20:0)	0.059	-0.89	$2.90x10^{-18}$
CER (26:0)	0.077	-0.89	$3.00x10^{-18}$
HCER (26:0)	0.082	-0.89	$3.00x10^{-18}$
HCER.d (18:0/24:0)	0.078	-0.89	$3.00x10^{-18}$
SM (24:0)	0.073	-0.89	$3.28x10^{-18}$
HCER (24:0)	0.078	-0.89	$3.99x10^{-18}$
PE.P (18:0/20:2)	0.069	-0.89	$4.05x10^{-18}$
PC (14:0/20:4)	0.068	-0.88	$4.26x10^{-18}$
PI (18:1/20:4)	0.072	-0.88	$4.65x10^{-18}$
PC (16:1/18:2)	0.070	-0.88	$6.09x10^{-18}$
PE.P (18:0/18:1)	0.072	-0.88	$6.42x10^{-18}$
PI (20:0/20:4)	0.066	-0.87	$9.72x10^{-18}$

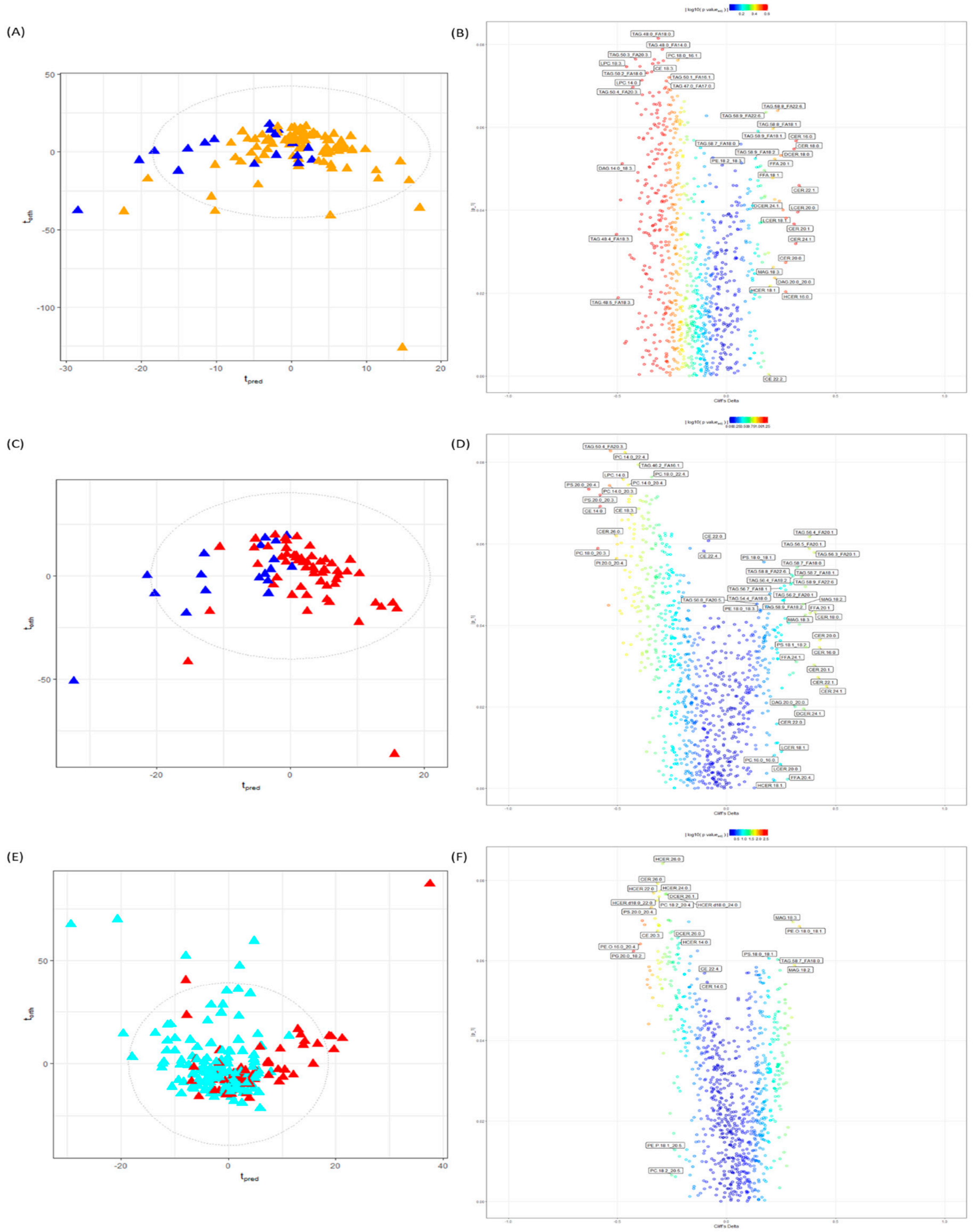

Figure S10 - Orthogonal Partial Least Squared (O-PLS-DA) and eruption plots between different severity classes of SARS-CoV-2 positive patients for the lipids. (A) O-PLS-DA SARS-CoV-2 severity group B (blue) and SARS-CoV-2 severity group D (orange) (R_2X=0.16, AUROC=0.64). (B) Eruption plot of severity group B vs severity group D. (C) O-

PLS-DA SARS-CoV-2 severity group B (blue) and SARS-CoV-2 severity group E (red) (R_2X=0.15, AUROC=0.79). (D) Eruption plot of severity group B vs severity group E. (E) O-PLS-DA SARS-CoV-2 severity group C (cyan) and SARS-CoV-2 severity group E (red) (R_2X=0.13, AUROC=0.72). Significant models could not be produced for severity group B vs C, group C vs D or D vs E.

Table S19 - OPLS loadings, Cliff's delta and adjusted p-values of the lipids for the comparison of severity class B and E. Only metabolites with significant p-values are shown in the table.

Lipid	OPLS loadings	Cliff's delta	Adjusted p-value
CE (14:0)	0.069	-0.58	$4.96x10^{-2}$
PC (18:0/20:3)	0.059	-0.59	$4.96x10^{-2}$
PS (20:0/20:3)	0.072	-0.58	$4.96x10^{-2}$
PC (20:0/20:4)	0.073	-0.63	$4.96x10^{-2}$

Table S20 - OPLS loadings, Cliff's delta and adjusted p-values of the lipids for the comparison of severity class C and E. Only metabolites with significant p-values are shown in the table.

Lipid	OPLS loadings	Cliff's delta	Adjusted p-value
PG (20:0/18:2)	0.062	-0.43	$2.39x10^{-3}$
PE.O (16:0/20:4)	0.064	-0.40	$6.14x10^{-3}$
PE.P (18:0/20:3)	0.070	-0.39	$6.18x10^{-3}$
PI (20:0/20:4)	0.069	-0.38	$7.94x10^{-3}$
PC (18:0/20:3)	0.054	-0.36	$9.36x10^{-3}$
PE.P (16:0/20:3)	0.055	-0.36	$9.36x10^{-3}$
PG (20:0/20:3)	0.044	-0.36	$9.83x10^{-3}$
PS (20:0/22:6)	0.058	-0.36	$9.83x10^{-3}$
PI (20:0/16:1)	0.058	-0.35	$9.99x10^{-3}$
PI (20:0/18:2)	0.053	-0.35	$9.99x10^{-3}$
PS (20:0/20:4)	0.073	-0.34	$1.21x10^{-2}$
PE.P (18:1/20:3)	0.056	-0.34	$1.30x10^{-2}$
PE (18:2/16:1)	0.051	-0.34	$1.33x10^{-2}$
HCER (22:0)	0.077	-0.33	$1.46x10^{-2}$
PE.O (18:0/18:1)	0.069	0.33	$1.46x10^{-2}$
LPC (20:0)	0.071	-0.33	$1.68x10^{-2}$
CE (16:0)	0.062	-0.31	$2.00x10^{-2}$
CE (20:3)	0.067	-0.32	$2.00x10^{-2}$
CER (26:0)	0.079	-0.32	$2.00x10^{-2}$
HCER (24:0)	0.077	-0.31	$2.00x10^{-2}$

HCER.d (18:0/22:0)	0.075	-0.32	$2.00x10^{-2}$
LPC (14:0)	0.069	-0.31	$2.00x10^{-2}$
LPC (16:0)	0.059	-0.31	$2.00x10^{-2}$
LPC (18:0)	0.069	-0.31	$2.00x10^{-2}$
MAG (18:2)	0.059	0.31	$2.00x10^{-2}$
PC (18:2/18:2)	0.069	-0.31	$2.00x10^{-2}$
PC (18:2/20:4)	0.076	-0.31	$2.00x10^{-2}$
PC (18:2/22:6)	0.060	-0.32	$2.00x10^{-2}$
PE.P (18:0/22:6)	0.049	-0.31	$2.00x10^{-2}$
SM (22:1)	0.056	-0.32	$2.00x10^{-2}$
MAG (18:3)	0.070	0.30	$2.15x10^{-2}$
PC (18:2/20:3)	0.054	-0.31	$2.15x10^{-2}$
PE.P (18:2/20:4)	0.058	-0.31	$2.15x10^{-2}$
PS (20:0/20:3)	0.055	-0.30	$2.15x10^{-2}$
PC (18:0/18:2)	0.060	-0.30	$2.45x10^{-2}$
TAG (56:3)_FA (20:1)	0.046	0.30	$2.45x10^{-2}$
PI (18:0/20:4)	0.049	-0.30	$2.54x10^{-2}$
SM (20:1)	0.053	-0.30	$2.45x10^{-2}$
PG (20:0/20:1)	0.053	-0.30	$2.58x10^{-2}$
HCER (26:0)	0.084	-0.29	$2.77x10^{-2}$
PC (14:0/22:6)	0.061	-0.29	$2.83x10^{-2}$
PG (20:0/18:1)	0.037	-0.29	$2.94x10^{-2}$
TAG (56:4)_FA (20:1)	0.048	0.29	$2.94x10^{-2}$
TAG (56:2)_FA (18:1)	0.042	0.28	$3.37x10^{-2}$
SM (24:0)	0.062	-0.28	$3.42x10^{-2}$
PE.P (16:0/22:6)	0.055	-0.28	$3.45x10^{-2}$
CER (16:0)	0.018	0.28	$3.50x10^{-2}$
HCER.d (18:0/24:0)	0.077	-0.28	$3.50x10^{-2}$
PC (16:0/22:6)	0.045	-0.28	$3.55x10^{-2}$
TAG (56:4)_FA (18:2)	0.044	0.28	$3.55x10^{-2}$
CER (24:1)	0.009	0.28	$3.93x10^{-2}$
DCER (26:1)	0.077	-0.28	$3.93x10^{-2}$
PE.P (18:0/20:4)	0.061	-0.28	$3.93x10^{-2}$
TAG (56:2)_FA (18:0)	0.035	0.28	$3.93x10^{-2}$
TAG (55:4)_FA (18:1)	0.030	0.27	$4.15x10^{-2}$
CER (20:0)	0.017	0.27	$4.18x10^{-2}$
FFA (20:1)	0.050	0.27	$4.18x10^{-2}$

HCER (20:0)	0.063	-0.27	4.18×10^{-2}
MAG (18:1)	0.054	0.27	4.18×10^{-2}
PC (18:1/20:4)	0.059	-0.27	4.18×10^{-2}
PE (16:0/18:1)	0.047	0.27	4.18×10^{-2}
TAG (54:3)_FA (18:0)	0.039	0.27	4.18×10^{-2}
TAG (56:3)_FA (18:0)	0.043	0.27	4.18×10^{-2}
TAG (56:7)_FA (18:1)	0.054	0.27	4.18×10^{-2}
TAG (58:7)_FA (18:1)	0.049	0.27	4.18×10^{-2}
PS (18:1/18:2)	0.050	0.27	4.19×10^{-2}
PC (18:0/22:4)	0.070	-0.27	4.28×10^{-2}
CE (22:5)	0.070	-0.26	4.47×10^{-2}
TAG (58:3)_FA (18:1)	0.043	0.27	4.47×10^{-2}
HCER.d (18:0/20:0)	0.060	-0.26	4.48×10^{-2}
CER (22:1)	0.012	0.26	4.54×10^{-2}
PE.P (18:1/20:4)	0.062	-0.26	4.74×10^{-2}
TAG (55:3)_FA (18:1)	0.029	0.26	4.74×10^{-2}
LPC (20:4)	0.054	-0.26	4.84×10^{-2}
PE.P (16:0/20:4)	0.061	-0.26	4.84×10^{-3}
PE.P (16:0/20:5)	0.022	-0.26	4.84×10^{-3}
PC (14:0/20:4)	0.070	-0.30	4.89×10^{-3}
PC (20:0/20:4)	0.067	-0.26	4.89×10^{-3}
PC (18:0/20:2)	0.036	0.26	4.89×10^{-3}
PE.P (18:2/18:2)	0.045	-0.26	4.89×10^{-3}

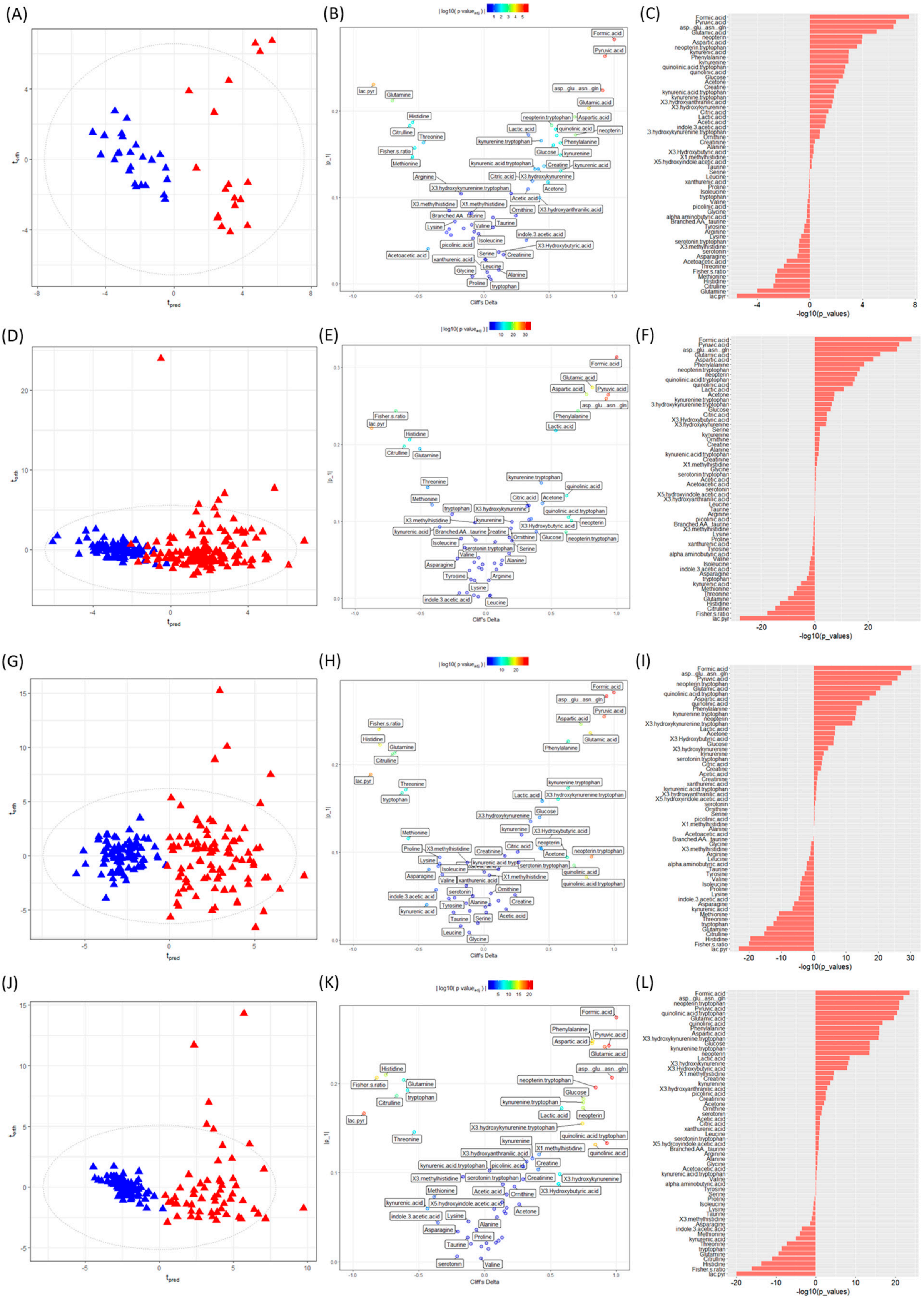
(A)
(B)
(C)
(D)
(E)
(F)
(G)
(H)
(I)
(J)
(K)
(L)
Cliff's Delta
-log10(p_values)

Figure S11 - Orthogonal Partial Least Squared (O-PLS-DA), eruption plots and −log 10 (p-values) between controls and SARS-CoV-2 positive patients of each severity group for the low molecular weight metabolites. (A) O-PLS-DA of controls (blue triangles) and SARS-CoV-2 group B severity patients (red triangles) (R_2X=0.22, AUROC=0.98).(B) Eruption plot of the controls vs group B severity group. (C) −log 10 (p-values) of the controls vs group B severity group. (D) O-PLS-DA of controls (blue triangles) and SARS-CoV-2 group C severity patients (red triangles) (R_2X=0.15, AUROC=0.99). (E) Eruption plot of the controls vs group C severity group. (F) −log 10 (p-values) of the controls vs group C severity group. (G) O-PLS-DA of controls (blue triangles) and SARS-CoV-2 group D severity patients (red triangles) (R_2X=0.19, AUROC=1.00). (H) Eruption plot of the controls vs group D severity group. (I) −log 10 (p-values) of the controls vs group D severity group. (J) O-PLS-DA of controls (blue triangles) and SARS-CoV-2 group E severity patients (red triangles) (R_2X=0.21, AUROC=1.00). (K) Eruption plot of the controls vs group E severity group. (L) −log 10 (p-values) of the controls vs group E severity group. Eruption plots are formed from the Cliff's delta (abscissa) and O-PLS-DA loadings (ordinate). Coloured by significance.

Table S21 - OPLS loadings, Cliff's delta and adjusted p-values of the low molecular weight metabolites for the comparison of the controls and SARS-CoV-2 positive patients in severity class B. Only metabolites with significant p-values are shown in the table.

Metabolite	OPLS loadings	Cliff's delta	Adjusted p-value
Formic acid	0.283	1.00	$1.68x10^{-6}$
Pyruvic acid	0.264	0.93	$8.01x10^{-6}$
Asp/Glu:Asn/Gln	0.224	0.91	$8.50x10^{-6}$
Lactate/pyruvate	0.231	-0.84	$4.14x10^{-5}$
Glutamic acid	0.204	0.80	$9.52x10^{-5}$
Neopterin	0.172	0.70	$7.91x10^{-4}$
Aspartic acid	0.193	0.70	$7.91x10^{-4}$
Glutamine	0.212	-0.70	$7.91x10^{-4}$
Neopterin/tryptophan	0.184	0.52	$1.66x10^{-3}$
Kynurenic acid	0.131	0.59	$5.49x10^{-3}$
Kynurenine	0.149	0.47	$1.66x10^{-3}$
Phenylalanine	0.163	0.59	$5.49x10^{-3}$
Citrulline	0.183	-0.57	$7.13x10^{-3}$
Quinolinic acid/tryptophan	0.172	0.56	$7.80x10^{-3}$
Quinolinic acid	0.176	0.55	$8.25x10^{-3}$
Histidine	0.187	-0.55	$8.25x10^{-3}$
Methionine	0.147	-0.55	$8.25x10^{-3}$
Glucose	0.161	0.53	$9.92x10^{-3}$
Fisher's ratio	0.157	-0.53	$1.02x10^{-2}$
Acetone	0.118	0.49	$1.84x10^{-2}$
Creatine	0.135	0.46	$2.71x10^{-2}$
Threonine	0.164	-0.46	$2.71x10^{-2}$

Kynurenic acid/tryptophan	0.133	0.39	$3.44x10^{-2}$
Kynurenine/tryptophan	0.166	0.44	$3.50x10^{-2}$
Acetoacetic acid	0.040	-0.43	$3.88x10^{-2}$
3-hydroxyanthranilic acid	0.100	0.43	$3.88x10^{-2}$
3-hydroxykynurenine	0.133	0.42	$4.49x10^{-2}$

Table S22 - OPLS loadings, Cliff's delta and adjusted p-values of the low molecular weight metabolites for the comparison of the controls and SARS-CoV-2 positive patients in severity class C. Only metabolites with significant p-values are shown in the table.

Metabolite	OPLS loadings	Cliff's delta	Adjusted p-value
Formic acid	0.313	1.00	$2.46x10^{-35}$
Pyruvic acid	0.265	0.93	$4.95x10^{-31}$
Asp/Glu:Asn/Gln	0.259	0.92	$2.23x10^{-30}$
Lactate/pyruvate	0.221	-0.88	$9.32x10^{-28}$
Glutamic acid	0.274	0.81	$3.79x10^{-24}$
Aspartic acid	0.265	0.77	$1.24x10^{-21}$
Phenylalanine	0.243	0.70	$2.64x10^{-18}$
Fisher's ratio	0.243	-0.69	$1.08x10^{-17}$
Neopterin/tryptophan	0.086	0.61	$8.43x10^{-17}$
Neopterin	0.101	0.65	$6.01x10^{-16}$
Quinolinic acid/tryptophan	0.106	0.63	$4.95x10^{-15}$
Citrulline	0.197	-0.63	$7.53x10^{-15}$
Quinolinic acid	0.134	0.62	$2.02x10^{-14}$
Histidine	0.206	-0.59	$3.70x10^{-13}$
Lactic acid	0.218	0.53	$3.92x10^{-11}$
Glutamine	0.194	-0.51	$3.95x10^{-10}$
Threonine	0.144	-0.45	$4.12x10^{-8}$
Acetone	0.123	0.43	$1.35x10^{-7}$
Kynurenine/tryptophan	0.150	0.42	$2.42x10^{-7}$
Methionine	0.121	-0.41	$4.40x10^{-7}$
3-hydroxykynurenine/tryptophan	0.103	0.35	$8.87x10^{-7}$
Glucose	0.087	0.38	$2.50x10^{-6}$
Kynurenic acid	0.093	-0.35	$1.55x10^{-5}$
Citric acid	0.120	0.33	$6.50x10^{-5}$
3-Hydroxybutyric acid	0.102	0.33	$6.85x10^{-5}$
3-Hydroxykynurenine	0.120	0.31	$1.45x10^{-4}$
Tryptophan	0.109	-0.26	$2.07x10^{-3}$

Asparagine	0.052	-0.22	$1.05x10^{-2}$
Indole-3-acetic acid	0.007	-0.21	$1.71x10^{-2}$
Serine	0.075	0.20	$2.17x10^{-2}$
Kynurenine	0.099	0.20	$2.28x10^{-2}$
Isoleucine	0.078	-0.19	$2.71x10^{-2}$
Ornithine	0.091	0.19	$2.89x10^{-2}$
Creatine	0.079	0.18	$4.08x10^{-2}$
Alanine	0.058	0.18	$4.22x10^{-2}$

Table S23 - OPLS loadings, Cliff's delta and adjusted p-values of the low molecular weight metabolites for the comparison of the controls and SARS-CoV-2 positive patients in severity class D. Only metabolites with significant p-values are shown in the table.

Metabolite	OPLS loadings	Cliff's delta	Adjusted p-value
Formic acid	0.282	1.00	$3.01x10^{-29}$
Asp/Glu:Asn/Gln	0.278	0.94	$2.41x10^{-26}$
Pyruvic acid	0.254	0.92	$2.01x10^{-25}$
Neopterin/tryptophan	0.095	0.82	$8.77x10^{-24}$
Lactate/pyruvate	0.189	-0.87	$7.12x10^{-23}$
Glutamic acid	0.236	0.82	$2.67x10^{-20}$
Fisher's ratio	0.240	-0.80	$7.12x10^{-20}$
Histidine	0.222	-0.80	$1.72x10^{-19}$
Quinolinic acid/tryptophan	0.072	0.79	$3.62x10^{-19}$
Aspartic acid	0.246	0.74	$2.95x10^{-17}$
Citrulline	0.212	-0.70	$2.68x10^{-15}$
Quinolinic acid	0.085	0.69	$4.10x10^{-15}$
Glutamine	0.213	-0.68	$9.50x10^{-15}$
Phenylalanine	0.227	0.65	$2.36x10^{-13}$
Kynurenine/tryptophan	0.173	0.64	$2.82x10^{-13}$
Neopterin	0.094	0.64	$4.01x10^{-13}$
Tryptophan	0.168	-0.63	$1.04x10^{-12}$
3-hydroxykynurenine/tryptophan	0.161	0.57	$3.21x10^{-12}$
Threonine	0.171	-0.60	$1.08x10^{-11}$
Methionine	0.116	-0.58	$4.99x10^{-11}$
Lactic acid	0.158	0.45	$5.41x10^{-7}$
Acetone	0.104	0.44	$7.24x10^{-7}$
Kynurenic acid	0.040	-0.44	$8.95x10^{-7}$
3-hydroxybutyric acid	0.105	0.43	$1.33x10^{-6}$

Glucose	0.140	0.43	$1.59x10^{-6}$
Asparagine	0.080	-0.42	$2.19x10^{-6}$
Indole-3-acetic acid	0.057	-0.36	$4.73x10^{-5}$
3-hydroxykynurenine	0.135	0.36	$6.30x10^{-5}$
Lysine	0.084	-0.35	$1.03x10^{-4}$
Proline	0.094	-0.34	$1.54x10^{-4}$
Isoleucine	0.087	-0.34	$1.60x10^{-4}$
Valine	0.075	-0.32	$4.01x10^{-4}$
Kynurenine	0.120	0.29	$1.40x10^{-3}$
Tyrosine	0.047	-0.27	$3.02x10^{-3}$
Serotonin/tryptophan	0.089	0.27	$3.02x10^{-3}$
Citric acid	0.100	0.26	$4.09x10^{-3}$
Creatine	0.052	0.24	$8.30x10^{-3}$
Taurine	0.032	-0.23	$1.00x10^{-2}$
Alpha-aminobutyric acid	0.048	-0.23	$1.29x10^{-2}$

Table S24 - OPLS loadings, Cliff's delta and adjusted p-values of the low molecular weight metabolites for the comparison of the controls and SARS-CoV-2 positive patients in severity class E. Only metabolites with significant p-values are shown in the table.

Metabolite	OPLS loadings	Cliff's delta	Adjusted p-value
Formic acid	0.274	1.00	$1.38x10^{-22}$
Asp/Glu:Asn/Gln	0.207	0.97	$2.62x10^{-21}$
Pyruvic acid	0.243	0.94	$1.43x10^{-20}$
Neopterin/tryptophan	0.196	0.84	$1.43x10^{-20}$
Quinolinic acid/tryptophan	0.133	0.93	$3.93x10^{-20}$
Lactate/pyruvate	0.167	-0.92	$9.10x10^{-20}$
Glutamic acid	0.242	0.91	$1.79x10^{-19}$
Quinolinic acid	0.131	0.84	$1.04x10^{-16}$
Fisher's ratio	0.207	-0.82	$4.71x10^{-16}$
Phenylalanine	0.248	0.82	$5.29x10^{-16}$
Aspartic acid	0.246	0.82	$5.32x10^{-16}$
3-hydroxykynurenine/tryptophan	0.155	0.74	$6.96x10^{-16}$
Histidine	0.210	-0.75	$8.42x10^{-14}$
Glucose	0.183	0.75	$8.35x10^{-12}$
Neopterin	0.173	0.75	$1.01x10^{-13}$
Kynurenine/tryptophan	0.179	0.75	$1.01x10^{-13}$
Citrulline	0.187	-0.67	$3.78x10^{-11}$

Glutamine	0.204	-0.61	$1.25x10^{-9}$
Lactic acid	0.172	0.58	$7.98x10^{-9}$
Tryptophan	0.192	-0.58	$7.98x10^{-9}$
3-hydroxykynurenine	0.098	0.57	$1.82x10^{-8}$
3-Hydroxybutyric acid	0.087	0.56	$3.21x10^{-8}$
Threonine	0.145	-0.53	$1.31x10^{-7}$
Kynurenic acid	0.060	-0.44	$2.81x10^{-5}$
1-methylhistidine	0.120	0.41	$5.82x10^{-5}$
Creatine	0.104	0.41	$8.15x10^{-5}$
Methionine	0.073	-0.38	$2.02x10^{-4}$
Kynurenine	0.116	0.36	$4.63x10^{-4}$
Indole-3-acetic acid	0.044	-0.35	$6.06x10^{-4}$
3-hydroxyanthranilic acid	0.112	0.32	$2.35x10^{-3}$
Creatinine	0.093	0.29	$5.29x10^{-3}$
Picolinic acid	0.107	0.29	$5.29x10^{-3}$
Acetone	0.065	0.26	$1.32x10^{-2}$
Ornithine	0.084	0.23	$3.55x10^{-2}$

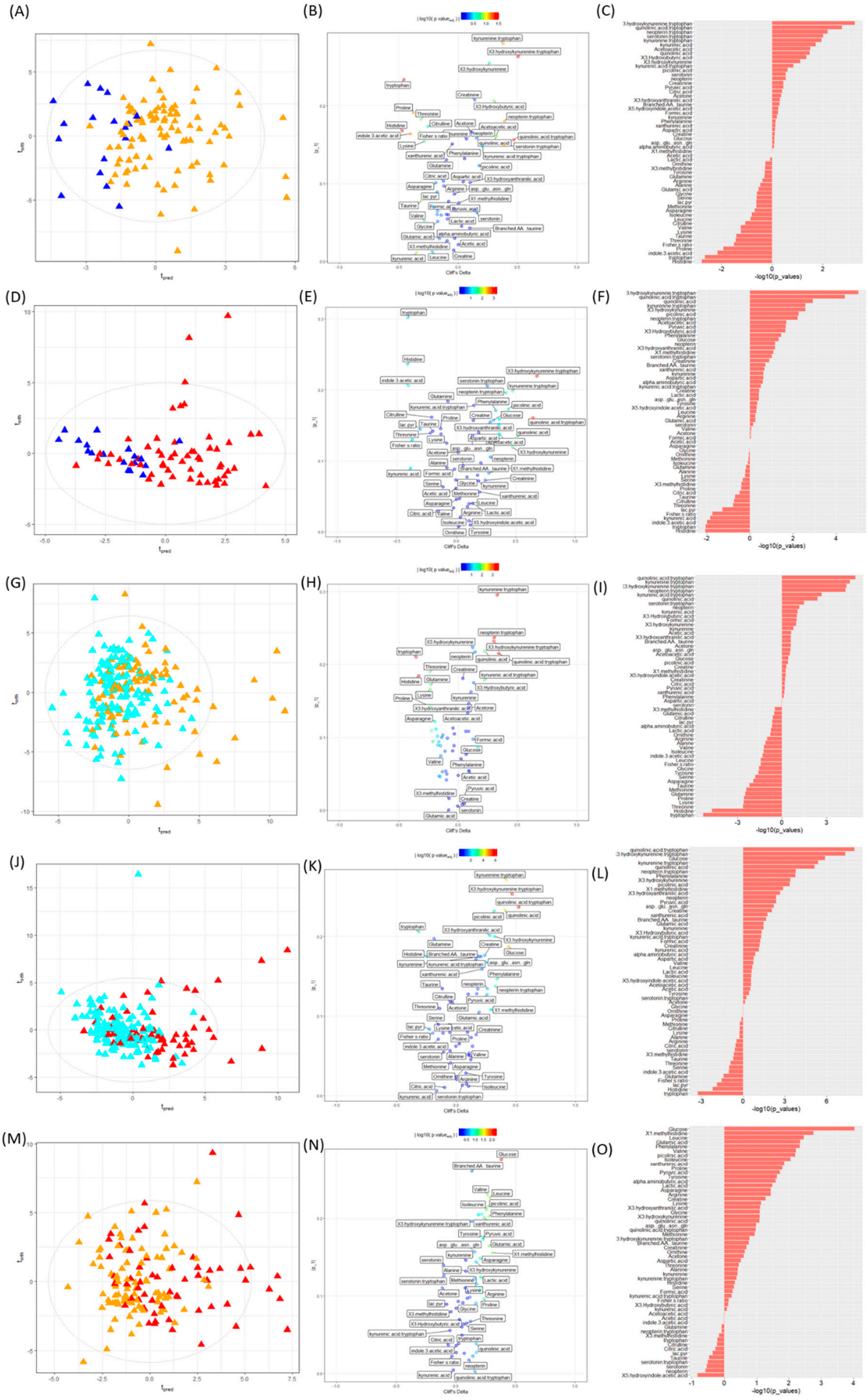
(A)
(B)
(C)
(D)
(E)
(F)
(G)
(H)
(I)
(J)
(K)
(L)
(M)
(N)
(O)
Cliff's Delta
-log10(p_values)

Figure S12 - Orthogonal Partial Least Squared (O-PLS-DA), eruption plots and −log 10 (p-values) between different severity classes of SARS-CoV-2 positive patients for the low molecular weight metabolites. (A) O-PLS-DA of SARS-CoV-2 severity group B (blue triangles) and SARS-CoV-2 severity group D (orange triangles) (R_2X=0.11, AUROC=0.76). (B) Eruption plot of group B vs group D. (C) −log 10 (p-values) of group B vs group D. (D) O-PLS-DA of SARS-CoV-2 severity group B (blue) and SARS-CoV-2 severity group E (red) (R_2X=0.09, AUROC=0.78). (E) Eruption plot of group B vs group E. (F) −log 10 (p-values) of group B vs group E. (G) O-PLS-DA of SARS-CoV-2 severity group C (cyan) and SARS-CoV-2 severity group D (orange) (R_2X=0.15, AUROC=0.65). (H) Eruption plot of group C vs group D. (I) −log 10 (p-values) of group C vs group D. (J) O-PLS-DA of SARS-CoV-2 severity group C (cyan) and SARS-CoV-2 severity group E (red) (R_2X=0.13, AUROC=0.74). (K) Eruption plot of group C vs group E. (I) −log 10 (p-values) of group C vs group E. (M) O-PLS-DA of SARS-CoV-2 severity group D (orange) and SARS-CoV-2 severity group E (red) (R_2X=0.12, AUROC=0.62). (N) Eruption plot of group D vs group E. (I) −log 10 (p-values) of group D vs group E. Comparison of severity group B vs severity group C produced no model (AUROC=0.58). Eruption plots are formed from the Cliff's delta (abscissa) and O-PLS-DA loadings (ordinate). Coloured by significance.

Table S25 - OPLS loadings, Cliff's delta and adjusted p-values for the low molecular weight metabolites for the comparison of severity class B and D. Only metabolites with significant p-values are shown in the table.

Metabolite	OPLS loadings	Cliff's delta	Adjusted p-value
3-hydroxykynurenine/tryptophan	0.263	0.50	$3.14x10^{-2}$
Tryptophan	0.234	-0.46	$3.22x10^{-2}$
Histidine	0.168	-0.47	$3.22x10^{-2}$
Quinolinic acid/tryptophan	0.156	0.47	$3.22x10^{-2}$

Table S26 - OPLS loadings, Cliff's delta and adjusted p-values for the low molecular weight metabolites for the comparison of severity class B and E. Only metabolites with significant p-values are shown in the table.

Metabolite	OPLS loadings	Cliff's delta	Adjusted p-value
3-hydroxykynurenine/tryptophan	0.220	0.68	$5.40x10^{-4}$
Quinolinic acid/tryptophan	0.161	0.65	$1.14x10^{-3}$
Quinolinic acid	0.133	0.51	$2.29x10^{-2}$
3-hydroxykynurenine	0.119	0.47	$3.05x10^{-2}$
Kynurenine/tryptophan	0.199	0.47	$3.05x10^{-2}$

Table S27 - OPLS loadings, Cliff's delta and adjusted p-values for the low molecular weight metabolites for the comparison of severity class C and D. Only metabolites with significant p-values are shown in the table.

Metabolite	OPLS loadings	Cliff's delta	Adjusted p-value

Tryptophan	0.232	-0.36	$2.68x10^{-4}$
Quinolinic acid/tryptophan	0.215	0.34	$3.14x10^{-4}$
Histidine	0.185	-0.33	$3.46x10^{-4}$
Kynurenine/tryptophan	0.296	0.33	$3.73x10^{-4}$
3-hydroxykynurenine/tryptophan	0.232	0.30	$4.80x10^{-4}$
Neopterin/tryptophan	0.237	0.30	$4.80x10^{-4}$
Lysine	0.166	-0.23	$1.58x10^{-2}$
Proline	0.142	-0.23	$1.58x10^{-2}$
Threonine	0.190	-0.24	$1.58x10^{-2}$
Kynurenic acid/tryptophan	0.179	0.22	$1.58x10^{-2}$
Glutamine	0.173	-0.23	$1.68x10^{-2}$
Quinolinic acid	0.219	0.22	$1.78x10^{-2}$
Methionine	0.109	-0.23	$1.78x10^{-2}$
Taurine	0.094	-0.21	$2.60x10^{-2}$
Asparagine	0.122	-0.20	$4.35x10^{-2}$

Table S28 - OPLS loadings, Cliff's delta and adjusted p-values for the low molecular weight metabolites for the comparison of severity class C and E. Only metabolites with significant p-values are shown in the table.

Metabolite	OPLS loadings	Cliff's delta	Adjusted p-value
Quinolinic acid/tryptophan	0.237	0.52	$5.79x10^{-7}$
3-hydroxykynurenine/tryptophan	0.253	0.47	$1.34x10^{-6}$
Glucose	0.187	0.44	$2.52x10^{-5}$
Kynurenine/tryptophan	0.271	0.42	$5.88x10^{-5}$
Quinolinic acid	0.234	0.41	$8.53x10^{-5}$
Phenylalanine	0.147	0.34	$1.46x10^{-3}$
Neopterin/tryptophan	0.138	0.33	$1.46x10^{-3}$
3-hydroxykynurenine	0.200	0.32	$2.77x10^{-3}$
Picolinic acid	0.232	0.32	$2.77x10^{-3}$
Tryptophan	0.206	-0.31	$2.77x10^{-3}$
1-methylhistidine	0.108	0.29	$6.86x10^{-3}$
3-hydroxyanthranilic acid	0.201	0.28	$1.01x10^{-2}$
Pyruvic acid	0.127	0.26	$1.75x10^{-2}$
Neopterin	0.135	0.26	$1.75x10^{-2}$
Asp/Glu:Asn/Gln	0.168	0.25	$2.28x10^{-2}$
Histidine	0.175	-0.25	$2.33x10^{-2}$
Creatine	0.176	0.24	$2.82x10^{-2}$

Lactate/pyruvate	0.084	-0.22	$4.32x10^{-2}$

Table S29 - OPLS loadings, Cliff's delta and adjusted p-values for the low molecular weight metabolites for the comparison of severity class D and E. Only metabolites with significant p-values are shown in the table.

Metabolite	OPLS loadings	Cliff's delta	Adjusted p-value
Glucose	0.276	0.38	$5.40x10^{-3}$

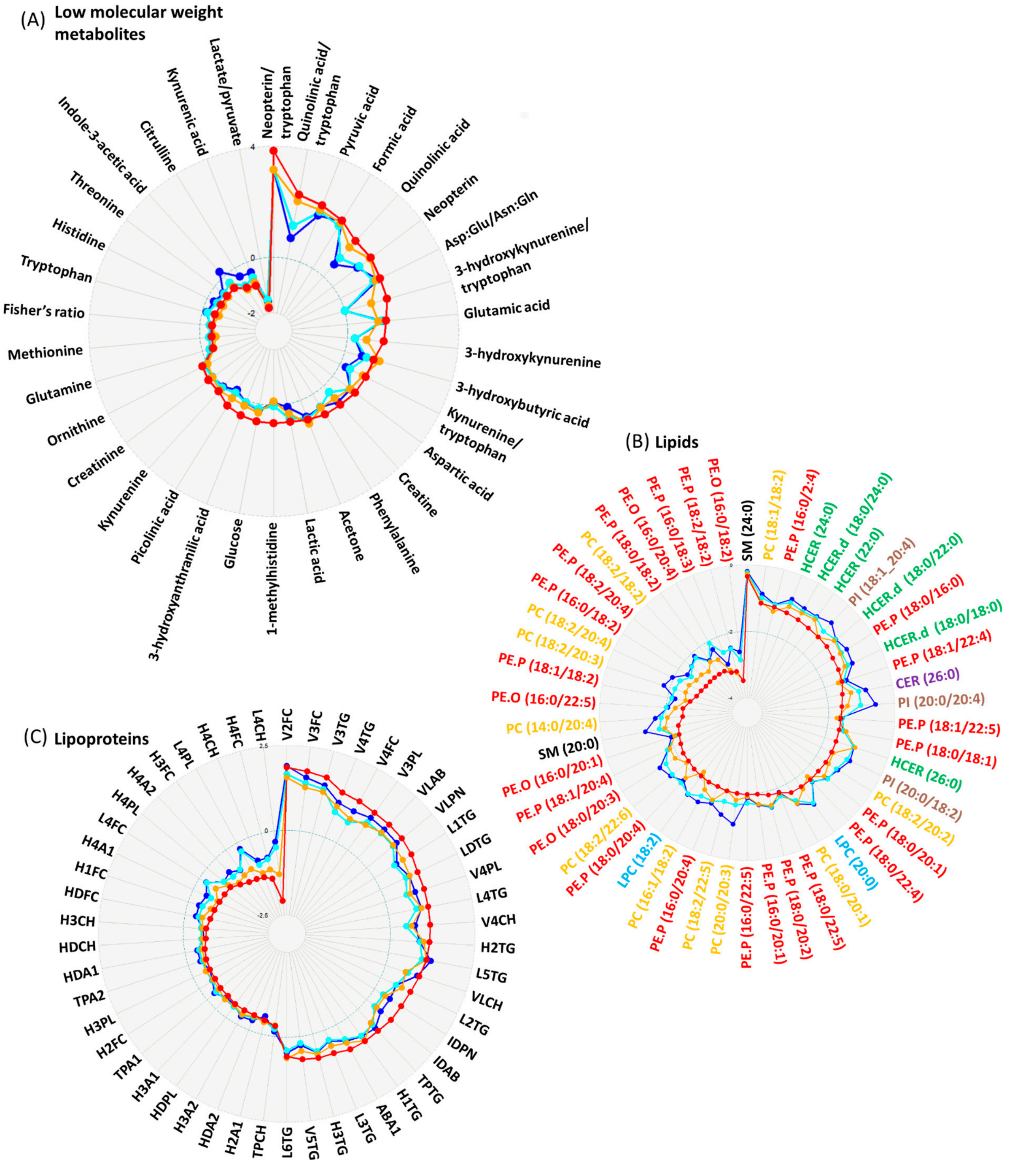

Figure S13– Radar plots showing the $\log_2$ fold change between controls and SARS-CoV-2 severity group E patients of the most significant ($p<0.05$) metabolites, lipids and lipoproteins ordered by $\log_2$ fold change. All radar plots are coloured in relation to fold change of each severity class in comparison to the controls. Controls vs group B (blue), controls vs group C severity (cyan), controls vs group D severity (orange) and controls vs group E severity (red). (A) Radar plot of the metabolites with a significant p-value from the controls vs group E severity model for the low molecular weight metabolites. (B) Radar plot of the top 50 most significant lipids from the controls vs group E severity model. (C) Radar plot of the top 50 most significant lipoproteins from the controls vs group E severity model.

Table S30 - Demographics for the severity group E patients who survived and who did not.

Patient	Sex	Age	Blood collection date	Date of death	Days between blood collection and death
1	Male	73	01/04/2020	NA	
2	Female	75	01/04/2020	NA	
3	Male	65	01/04/2020	NA	
4	Female	74	01/04/2020	NA	
5	Female	68	01/04/2020	NA	
6	Male	77	01/04/2020	NA	
7	Male	77	01/04/2020	NA	
8	Male	73	01/04/2020	NA	
9	Male	73	01/04/2020	NA	
10	Male	72	01/04/2020	NA	
11	Female	72	01/04/2020	NA	
12	Female	66	01/04/2020	NA	
13	Male	76	01/04/2020	NA	
14	Female	70	01/04/2020	17/04/2020	16
15	Male	77	01/04/2020	14/04/2020	13
16	Male	77	01/04/2020	21/05/2020	50
17	Male	73	01/04/2020	28/04/2020	27
18	Male	73	01/04/2020	10/04/2020	9
19	Female	79	01/04/2020	02/05/2020	31
20	Female	75	01/04/2020	02/05/2020	31
21	Male	70	01/04/2020	01/06/2020	61
22	Female	68	07/04/2020	21/04/2020	14
23	Female	80	09/04/2020	unknown	-
24	Male	66	09/04/2020	17/04/2020	8
25	Female	79	09/04/2020	08/05/2020	29
26	Male	79	09/04/2020	03/05/2020	24

Table S31 - OPLS loadings, Cliff's delta and adjusted p-values of the combined model (low molecular weight metabolites, lipoproteins and lipids) for the comparison group E severity patients comparing who survived and who died, samples were age matched. Only metabolites with significant p-values are shown in the table.

Metabolite	OPLS loadings	Cliff's delta	Adjusted p-value
HCER (16:0)	0.057	0.92	$9.15x10^{-3}$
HCER (20:0)	0.055	0.92	$9.15x10^{-3}$
HCER (24:0)	0.063	0.91	$9.15x10^{-3}$
HCER (24:1)	0.062	0.91	$9.15x10^{-3}$
HCER (26:0)	0.061	0.95	$9.15x10^{-3}$
HCER (26:1)	0.063	0.92	$9.15x10^{-3}$
HCER.d (18:0/24:0)	0.063	0.91	$9.15x10^{-3}$
HCER.d (18:0/24:1)	0.062	0.94	$9.15x10^{-3}$
PE.O (18:0/18:1)	0.056	-0.98	$9.15x10^{-3}$
PE.P (16:0/18:0)	0.053	-0.93	$9.15x10^{-3}$
HCER (22:0)	0.063	0.89	$9.41x10^{-3}$
HCER.d (18:0/22:0)	0.063	0.89	$9.41x10^{-3}$
PE (16:0/18:1)	0.061	-0.88	$1.07x10^{-2}$
TAG (54:1)_FA (16:0)	0.051	-0.87	$1.07x10^{-2}$
TAG (54:1)_FA (20:0)	0.053	-0.87	$1.07x10^{-2}$
TAG (56:1)_FA (16:0)	0.055	-0.87	$1.07x10^{-2}$
H3FC	0.057	0.83	$1.19x10^{-2}$
HCER (18:0)	0.052	0.83	$1.19x10^{-2}$
PE (18:0/18:0)	0.059	-0.85	$1.19x10^{-2}$
PE (18:0/18:1)	0.058	-0.83	$1.19x10^{-2}$
PE (18:1/18:1)	0.059	-0.82	$1.19x10^{-2}$
PE (18:1/18:3)	0.044	-0.82	$1.19x10^{-2}$
PI (16:0/20:2)	0.040	-0.82	$1.19x10^{-2}$
SM (26:0)	0.060	0.86	$1.19x10^{-2}$
TAG (50:1)_FA (16:0)	0.053	-0.82	$1.19x10^{-2}$
TAG (50:2)_FA (16:0)	0.048	-0.82	$1.19x10^{-2}$
TAG (52:2)_FA (18:1)	0.055	-0.82	$1.19x10^{-2}$
TAG (54:2)_FA (20:0)	0.036	-0.82	$1.19x10^{-2}$
TAG (56:1)_FA (18:1)	0.053	-0.82	$1.19x10^{-2}$
TAG (56:2)_FA (16:0)	0.054	-0.83	$1.19x10^{-2}$
TAG (56:2)_FA (20:0)	0.053	-0.83	$1.19x10^{-2}$
TAG (58:2)_FA (18:1)	0.038	-0.83	$1.19x10^{-2}$

HCER.d (18:0/20:0)	0.050	0.81	$1.25x10^{-2}$
PE (16:0/16:1)	0.050	-0.81	$1.25x10^{-2}$
PE (16:0/18:2)	0.056	-0.81	$1.25x10^{-2}$
PE (18:0/18:3)	0.048	-0.81	$1.25x10^{-2}$
PG (18:1/18:1)	0.047	-0.81	$1.25x10^{-2}$
HDCH	0.050	0.80	$1.30x10^{-2}$
VLPN	0.050	-0.79	$1.30x10^{-2}$
VLAB	0.050	-0.79	$1.30x10^{-2}$
DAG (16:0/16:0)	0.040	-0.79	$1.30x10^{-2}$
DAG (18:0/18:3)	0.038	-0.79	$1.30x10^{-2}$
HCER (14:0)	0.049	0.79	$1.30x10^{-2}$
MAG (18:3)	0.040	-0.79	$1.30x10^{-2}$
PC (18:1/18:3)	0.048	-0.79	$1.30x10^{-2}$
PI (18:0/18:3)	0.025	-0.80	$1.30x10^{-2}$
TAG (50:1)_FA (18:1)	0.056	-0.79	$1.30x10^{-2}$
TAG (51:2)_FA (16:0)	0.037	-0.80	$1.30x10^{-2}$
TAG (52:1)_FA (18:1)	0.060	-0.79	$1.30x10^{-2}$
TAG (55:1)_FA (16:0)	0.048	-0.80	$1.30x10^{-2}$
TAG (55:1)_FA (18:1)	0.048	-0.80	$1.30x10^{-2}$
TAG (56:3)_FA (16:0)	0.043	-0.80	$1.30x10^{-2}$
MAG (18:1)	0.041	-0.78	$1.36x10^{-2}$
PC (16:0/18:3)	0.044	-0.78	$1.36x10^{-2}$
PC (18:0/20:4)	0.053	0.78	$1.36x10^{-2}$
PE (16:0/18:3)	0.048	-0.78	$1.36x10^{-2}$
TAG (51:1)_FA (16:0)	0.042	-0.78	$1.36x10^{-2}$
TAG (52:4)_FA (16:0)	0.046	-0.78	$1.36x10^{-2}$
TAG (53:0)_FA (16:0)	0.039	-0.78	$1.36x10^{-2}$
TAG (58:3)_FA (18:1)	0.050	-0.78	$1.36x10^{-2}$
L6FC	0.055	0.75	$1.51x10^{-2}$
FFA (24:0)	0.041	-0.76	$1.51x10^{-2}$
TAG (50:5)_FA (18:1)	0.035	-0.76	$1.54x10^{-2}$
TAG (52:0)_FA (20:0)	0.042	-0.76	$1.54x10^{-2}$
LPE (18:3)	0.041	-0.75	$1.66x10^{-2}$
MAG (20:3)	0.040	-0.75	$1.66x10^{-2}$
PC (18:1/18:1)	0.040	-0.75	$1.66x10^{-2}$
SM (26:1)	0.043	0.75	$1.66x10^{-2}$
TAG (52:3)_FA (18:3)	0.031	-0.75	$1.66x10^{-2}$

TAG (54:0)_FA (16:0)	0.050	-0.75	$1.66x10^{-2}$
TAG (54:4)_FA (18:1)	0.053	-0.75	$1.66x10^{-2}$
DAG (16:0/18:0)	0.037	-0.74	$1.72x10^{-2}$
DAG (16:0/18:1)	0.047	-0.74	$1.72x10^{-2}$
LPE (18:1)	0.051	-0.74	$1.72x10^{-2}$
PC (20:0/20:4)	0.052	0.74	$1.72x10^{-2}$
PE (18:0/20:1)	0.047	-0.74	$1.72x10^{-2}$
TAG (51:1)_FA (18:1)	0.044	-0.74	$1.72x10^{-2}$
TAG (52:1)_FA (16:0)	0.057	-0.74	$1.72x10^{-2}$
TAG (52:2)_FA (20:0)	0.047	-0.74	$1.72x10^{-2}$
TAG (53:1)_FA (16:0)	0.038	-0.74	$1.72x10^{-2}$
TAG (54:2)_FA (16:0)	0.046	-0.74	$1.72x10^{-2}$
TAG (54:5)_FA (18:1)	0.051	-0.74	$1.72x10^{-2}$
V5TG	0.048	-0.73	$1.79x10^{-2}$
H1FC	0.054	0.73	$1.79x10^{-2}$
DCER (26:1)	0.055	0.73	$1.79x10^{-2}$
MAG (18:0)	0.036	-0.73	$1.79x10^{-2}$
PC (18:0/18:3)	0.035	-0.73	$1.79x10^{-2}$
PS (20:0/18:1)	0.041	-0.73	$1.79x10^{-2}$
TAG (51:1)_FA (17:0)	0.039	-0.73	$1.79x10^{-2}$
TAG (52:4)_FA (18:3)	0.039	-0.73	$1.79x10^{-2}$
TAG (54:4)_FA (18:3)	0.038	-0.73	$1.79x10^{-2}$
TAG (54:5)_FA (18:0)	0.035	-0.73	$1.79x10^{-2}$
TAG (54:6)_FA (18:1)	0.045	-0.73	$1.79x10^{-2}$
TAG (57:10)_FA (22:6)	0.034	-0.73	$1.79x10^{-2}$
TPTG	0.046	-0.72	$1.84x10^{-2}$
VLCH	0.043	-0.72	$1.84x10^{-2}$
V4FC	0.050	-0.72	$1.84x10^{-2}$
CE (16:0)	0.051	0.72	$1.84x10^{-2}$
DAG (16:0/18:3)	0.036	-0.72	$1.84x10^{-2}$
HCER.d (18:0/26:1)	0.048	0.72	$1.84x10^{-2}$
PE (18:1/20:1)	0.044	-0.72	$1.84x10^{-2}$
PS (18:0/18:1)	0.057	-0.72	$1.84x10^{-2}$
TAG (49:1)_FA (18:1)	0.032	-0.72	$1.84x10^{-2}$
TAG (50:3)_FA (18:3)	0.035	-0.72	$1.84x10^{-2}$
TAG (52:3)_FA (18:1)	0.052	-0.72	$1.84x10^{-2}$
TAG (52:7)_FA (18:1)	0.034	-0.72	$1.84x10^{-2}$

TAG (54:2)_FA (18:1)	0.054	-0.72	$1.84x10^{-2}$
TAG (55:5)_FA (18:1)	0.042	-0.72	$1.84x10^{-2}$
TAG (57:2)_FA (18:1)	0.051	-0.72	$1.84x10^{-2}$
Branched AA/Taurine	0.036	-0.70	$1.94x10^{-2}$
HDFC	0.053	0.70	$1.94x10^{-2}$
DAG (18:1/20:1)	0.051	-0.70	$1.94x10^{-2}$
SM (20:1)	0.048	0.70	$1.94x10^{-2}$
TAG (48:1)_FA (18:1)	0.040	-0.70	$1.94x10^{-2}$
TAG (50:4)_FA (16:0)	0.031	-0.70	$1.94x10^{-2}$
TAG (50:5)_FA (16:0)	0.033	-0.70	$1.94x10^{-2}$
TAG (52:6)_FA (16:0)	0.033	-0.70	$1.94x10^{-2}$
TAG (52:6)_FA (18:1)	0.035	-0.70	$1.94x10^{-2}$
TAG (53:1)_FA (18:1)	0.040	-0.70	$1.94x10^{-2}$
TAG (53:2)_FA (16:0)	0.044	-0.70	$1.94x10^{-2}$
TAG (54:2)_FA (20:1)	0.048	-0.70	$1.94x10^{-2}$
TAG (54:3)_FA (18:3)	0.032	-0.70	$1.94x10^{-2}$
TAG (54:7)_FA (18:1)	0.038	-0.70	$1.94x10^{-2}$
LDCH	0.054	0.69	$2.04x10^{-2}$
V4TG	0.043	-0.69	$2.04x10^{-2}$
MAG (20:2)	0.029	-0.69	$2.04x10^{-2}$
PE (16:0/22:5)	0.049	-0.69	$2.04x10^{-2}$
PE (18:0/18:2)	0.050	-0.69	$2.04x10^{-2}$
PE (18:1/18:2)	0.046	-0.69	$2.04x10^{-2}$
PI (16:0/20:3)	0.035	-0.69	$2.04x10^{-2}$
TAG (48:4)_FA (18:1)	0.042	-0.69	$2.04x10^{-2}$
TAG (49:0)_FA (16:0)	0.031	-0.69	$2.04x10^{-2}$
TAG (49:0)_FA (17:0)	0.030	-0.69	$2.04x10^{-2}$
TAG (49:1)_FA (16:0)	0.033	-0.69	$2.04x10^{-2}$
TAG (49:3)_FA (16:0)	0.023	-0.69	$2.04x10^{-2}$
TAG (51:2)_FA (17:0)	0.026	-0.69	$2.04x10^{-2}$
TAG (52:1)_FA (18:0)	0.055	-0.69	$2.04x10^{-2}$
TAG (52:3)_FA (18:2)	0.040	-0.69	$2.04x10^{-2}$
Taurine	0.033	0.68	$2.18x10^{-2}$
L6PN	0.046	0.68	$2.18x10^{-2}$
V2FC	0.041	-0.68	$2.18x10^{-2}$
V4PL	0.046	-0.68	$2.18x10^{-2}$
L6AB	0.046	0.68	$2.18x10^{-2}$

MAG (22:6)	0.032	-0.68	$2.18x10^{-2}$
PG (20:0/18:2)	0.052	0.68	$2.18x10^{-2}$
TAG (51:0)_FA (16:0)	0.034	-0.68	$2.18x10^{-2}$
TAG (52:1)_FA (20:0)	0.033	-0.68	$2.18x10^{-2}$
TAG (52:5)_FA (16:0)	0.037	-0.68	$2.18x10^{-2}$
TAG (53:2)_FA (17:0)	0.046	-0.68	$2.18x10^{-2}$
TAG (53:2)_FA (18:1)	0.050	-0.68	$2.18x10^{-2}$
TAG (54:5)_FA (18:3)	0.039	-0.68	$2.18x10^{-2}$
TAG (56:3)_FA (20:0)	0.047	-0.68	$2.18x10^{-2}$
H4FC	0.052	0.67	$2.22x10^{-2}$
IDTG	0.039	-0.67	$2.31x10^{-2}$
V3FC	0.040	-0.67	$2.31x10^{-2}$
CE (22:4)	0.049	0.67	$2.31x10^{-2}$
DAG (16:0/20:5)	0.024	-0.67	$2.31x10^{-2}$
LPE (18:2)	0.038	-0.67	$2.31x10^{-2}$
MAG (16:0)	0.037	-0.67	$2.31x10^{-2}$
MAG (18:2)	0.040	-0.67	$2.31x10^{-2}$
MAG (22:4)	0.037	-0.67	$2.31x10^{-2}$
PI (16:0/20:4)	0.036	-0.67	$2.31x10^{-2}$
TAG (48:4)_FA (16:0)	0.029	-0.67	$2.31x10^{-2}$
TAG (51:1)_FA (18:0)	0.029	-0.67	$2.31x10^{-2}$
TAG (51:2)_FA (16:1)	0.032	-0.67	$2.31x10^{-2}$
TAG (51:4)_FA (18:3)	0.025	-0.67	$2.31x10^{-2}$
TAG (53:4)_FA (16:0)	0.029	-0.67	$2.31x10^{-2}$
TAG (54:2)_FA (18:0)	0.055	-0.67	$2.31x10^{-2}$
L6CH	0.048	0.66	$2.46x10^{-2}$
H1CH	0.040	0.66	$2.46x10^{-2}$
DAG (18:0/18:1)	0.051	-0.66	$2.46x10^{-2}$
MAG (20:4)	0.036	-0.66	$2.46x10^{-2}$
PC (16:1/18:1)	0.037	-0.66	$2.46x10^{-2}$
PE (16:0/20:1)	0.050	-0.66	$2.46x10^{-2}$
PE (18:0/16:0)	0.045	-0.66	$2.46x10^{-2}$
PE (18:1/20:3)	0.046	-0.66	$2.46x10^{-2}$
PG (20:0/20:1)	0.047	0.66	$2.46x10^{-2}$
TAG (48:1)_FA (16:0)	0.039	-0.66	$2.46x10^{-2}$
TAG (49:1)_FA (16:1)	0.025	-0.66	$2.46x10^{-2}$
TAG (49:2)_FA (18:1)	0.027	-0.66	$2.46x10^{-2}$

TAG (50:2)_FA (18:1)	0.047	-0.66	$2.46x10^{-2}$
TAG (51:2)_FA (18:1)	0.043	-0.66	$2.46x10^{-2}$
TAG (52:5)_FA (22:5)	0.029	-0.66	$2.46x10^{-2}$
TAG (53:1)_FA (17:0)	0.036	-0.66	$2.46x10^{-2}$
TAG (54:4)_FA (16:0)	0.037	-0.66	$2.46x10^{-2}$
TAG (56:3)_FA (18:2)	0.040	-0.66	$2.46x10^{-2}$
α-aminobutyric acid	0.029	-0.64	$2.46x10^{-2}$
Isoleucine	0.023	-0.64	$2.60x10^{-2}$
DAG (18:1/20:5)	0.026	-0.64	$2.60x10^{-2}$
MAG (22:5)	0.037	-0.64	$2.60x10^{-2}$
PC (16:0/18:1)	0.037	-0.64	$2.60x10^{-2}$
PI (18:0/20:5)	0.023	-0.64	$2.60x10^{-2}$
PI (18:1/18:1)	0.031	-0.64	$2.60x10^{-2}$
TAG (46:3)_FA (16:0)	0.030	-0.64	$2.60x10^{-2}$
TAG (48:1)_FA (14:0)	0.036	-0.64	$2.60x10^{-2}$
TAG (48:2)_FA (16:0)	0.022	-0.64	$2.60x10^{-2}$
TAG (49:2)_FA (16:0)	0.022	-0.64	$2.60x10^{-2}$
TAG (50:0)_FA (18:0)	0.035	-0.64	$2.60x10^{-2}$
TAG (50:2)_FA (16:1)	0.043	-0.64	$2.60x10^{-2}$
TAG (52:0)_FA (16:0)	0.045	-0.64	$2.60x10^{-2}$
TAG (52:1)_FA (20:1)	0.039	-0.64	$2.60x10^{-2}$
TAG (52:2)_FA (20:2)	0.034	-0.64	$2.60x10^{-2}$
TAG (52:3)_FA (20:2)	0.025	-0.64	$2.60x10^{-2}$
TAG (53:1)_FA (18:0)	0.036	-0.64	$2.60x10^{-2}$
TAG (54:1)_FA (18:0)	0.053	-0.64	$2.60x10^{-2}$
TAG (54:1)_FA (18:1)	0.053	-0.64	$2.60x10^{-2}$
TAG (56:7)_FA (20:5)	0.032	-0.64	$2.60x10^{-2}$
VLFC	0.038	-0.63	$2.70x10^{-2}$
V5FC	0.042	-0.63	$2.70x10^{-2}$
H2TG	0.049	-0.63	$2.70x10^{-2}$
CE (20:3)	0.039	0.63	$2.70x10^{-2}$
DAG (14:0/18:3)	0.029	-0.63	$2.70x10^{-2}$
DAG (16:0/20:4)	0.033	-0.63	$2.70x10^{-2}$
DAG (18:0/18:2)	0.034	-0.63	$2.70x10^{-2}$
DAG (18:1/18:1)	0.043	-0.63	$2.70x10^{-2}$
PE (18:0/20:2)	0.047	-0.63	$2.70x10^{-2}$
PE.P (16:0/22:6)	0.034	0.63	$2.70x10^{-2}$

PE.P (18:0/20:4)	0.040	0.63	$2.70x10^{-2}$
PI (16:0/18:1)	0.041	-0.63	$2.70x10^{-2}$
TAG (47:1)_FA (16:0)	0.025	-0.63	$2.70x10^{-2}$
TAG (49:0)_FA (18:0)	0.024	-0.63	$2.70x10^{-2}$
TAG (49:3)_FA (18:3)	0.021	-0.63	$2.70x10^{-2}$
TAG (50:0)_FA (16:0)	0.041	-0.63	$2.70x10^{-2}$
TAG (50:3)_FA (16:0)	0.029	-0.63	$2.70x10^{-2}$
TAG (51:3)_FA (18:3)	0.023	-0.63	$2.70x10^{-2}$
TAG (52:7)_FA (16:0)	0.030	-0.63	$2.70x10^{-2}$
TAG (54:3)_FA (16:0)	0.036	-0.63	$2.70x10^{-2}$
TAG (54:3)_FA (18:1)	0.051	-0.63	$2.70x10^{-2}$
TAG (54:3)_FA (20:2)	0.038	-0.63	$2.70x10^{-2}$
TAG (54:8)_FA (18:2)	0.031	-0.63	$2.70x10^{-2}$
TAG (56:2)_FA (18:0)	0.044	-0.63	$2.70x10^{-2}$
TAG (56:4)_FA (16:0)	0.036	-0.63	$2.70x10^{-2}$
TAG (56:7)_FA (18:1)	0.041	-0.63	$2.70x10^{-2}$
Tyrosine	0.032	-0.62	$2.90x10^{-2}$
V5CH	0.042	-0.62	$2.90x10^{-2}$
L3FC	0.047	0.62	$2.90x10^{-2}$
CE (20:0)	0.046	0.62	$2.90x10^{-2}$
FFA (20:0)	0.038	-0.62	$2.90x10^{-2}$
LPE (20:1)	0.046	-0.62	$2.90x10^{-2}$
PC (14:0/18:1)	0.038	-0.62	$2.90x10^{-2}$
PC (18:1/20:2)	0.037	-0.62	$2.90x10^{-2}$
PC (18:2/18:3)	0.042	-0.62	$2.90x10^{-2}$
TAG (45:1)_FA (16:0)	0.025	-0.62	$2.90x10^{-2}$
TAG (48:1)_FA (16:1)	0.035	-0.62	$2.90x10^{-2}$
TAG (52:2)_FA (14:0)	0.028	-0.62	$2.90x10^{-2}$
TAG (52:3)_FA (20:3)	0.035	-0.62	$2.90x10^{-2}$
TAG (52:5)_FA (20:5)	0.030	-0.62	$2.90x10^{-2}$
TAG (53:4)_FA (18:3)	0.026	-0.62	$2.90x10^{-2}$
TAG (54:1)_FA (20:1)	0.041	-0.62	$2.90x10^{-2}$
TAG (54:2)_FA (20:2)	0.032	-0.62	$2.90x10^{-2}$
TAG (54:6)_FA (20:5)	0.031	-0.62	$2.90x10^{-2}$
TAG (55:2)_FA (18:2)	0.028	-0.62	$2.90x10^{-2}$
TAG (56:5)_FA (18:1)	0.044	-0.62	$2.90x10^{-2}$
LDFC	0.051	0.61	$3.08x10^{-2}$

HDA1	0.045	0.61	$3.08x10^{-2}$
V5PL	0.038	-0.61	$3.08x10^{-2}$
L6PL	0.047	0.61	$3.08x10^{-2}$
H4A1	0.045	0.61	$3.08x10^{-2}$
DAG (16:1/18:0)	0.035	-0.61	$3.08x10^{-2}$
DAG (18:2/18:3)	0.031	-0.61	$3.08x10^{-2}$
PE (18:1/22:6)	0.047	-0.61	$3.08x10^{-2}$
PE.P (16:0/16:1)	0.034	-0.61	$3.08x10^{-2}$
PE.P (16:0/20:4)	0.042	0.61	$3.08x10^{-2}$
PI (16:0/22:4)	0.019	-0.61	$3.08x10^{-2}$
TAG (44:2)_FA (16:0)	0.020	-0.61	$3.08x10^{-2}$
TAG (44:2)_FA (18:1)	0.025	-0.61	$3.08x10^{-2}$
TAG (46:3)_FA (18:1)	0.024	-0.61	$3.08x10^{-2}$
TAG (47:0)_FA (16:0)	0.025	-0.61	$3.08x10^{-2}$
TAG (52:3)_FA (14:0)	0.024	-0.61	$3.08x10^{-2}$
TAG (54:4)_FA (20:3)	0.036	-0.61	$3.08x10^{-2}$
TAG (54:5)_FA (16:0)	0.041	-0.61	$3.08x10^{-2}$
TAG (54:5)_FA (20:5)	0.029	-0.61	$3.08x10^{-2}$
TAG (54:5)_FA (22:5)	0.033	-0.61	$3.08x10^{-2}$
TAG (54:8)_FA (18:3)	0.035	-0.61	$3.08x10^{-2}$
TAG (56:2)_FA (20:1)	0.047	-0.61	$3.08x10^{-2}$
TAG (56:3)_FA (18:1)	0.049	-0.61	$3.08x10^{-2}$
TAG (56:5)_FA (16:0)	0.033	-0.61	$3.08x10^{-2}$
TPA1	0.047	0.60	$3.30x10^{-2}$
LDPL	0.049	0.60	$3.30x10^{-2}$
DAG (16:0/22:5)	0.029	-0.60	$3.30x10^{-2}$
FFA (20:5)	0.024	-0.60	$3.30x10^{-2}$
HCER.d (18:0/26:0)	0.036	0.60	$3.30x10^{-2}$
PC (16:0/16:1)	0.035	-0.60	$3.30x10^{-2}$
PE (16:0/20:3)	0.045	-0.60	$3.30x10^{-2}$
PE (16:0/20:4)	0.048	-0.60	$3.30x10^{-2}$
PE (18:0/22:5)	0.046	-0.60	$3.30x10^{-2}$
PG (18:2/18:2)	0.028	-0.60	$3.30x10^{-2}$
PI (16:0/22:5)	0.041	-0.60	$3.30x10^{-2}$
PI (18:1/18:2)	0.030	-0.60	$3.30x10^{-2}$
PS (20:0/18:3)	0.034	-0.60	$3.30x10^{-2}$
SM (18:0)	0.032	0.60	$3.30x10^{-2}$

SM (22:1)	0.046	0.60	$3.30x10^{-2}$
TAG (48:0)_FA (16:0)	0.036	-0.60	$3.30x10^{-2}$
TAG (48:2)_FA (14:0)	0.021	-0.60	$3.30x10^{-2}$
TAG (48:3)_FA (18:3)	0.025	-0.60	$3.30x10^{-2}$
TAG (51:0)_FA (18:0)	0.031	-0.60	$3.30x10^{-2}$
TAG (51:3)_FA (17:0)	0.020	-0.60	$3.30x10^{-2}$
TAG (54:3)_FA (20:3)	0.037	-0.60	$3.30x10^{-2}$
TAG (56:3)_FA (18:0)	0.039	-0.60	$3.30x10^{-2}$
TAG (56:3)_FA (20:2)	0.043	-0.60	$3.30x10^{-2}$
TAG (56:4)_FA (22:4)	0.030	-0.60	$3.30x10^{-2}$
CE (22:6)	0.048	0.59	$3.59x10^{-2}$
DAG (18:1/20:2)	0.039	-0.59	$3.59x10^{-2}$
DAG (18:2/20:5)	0.024	-0.59	$3.59x10^{-2}$
PE.O (18:0/18:2)	0.038	-0.59	$3.59x10^{-2}$
PE.P (18:1/20:4)	0.043	0.59	$3.59x10^{-2}$
TAG (42:1)_FA (16:0)	0.021	-0.59	$3.59x10^{-2}$
TAG (47:1)_FA (16:1)	0.023	-0.59	$3.59x10^{-2}$
TAG (48:3)_FA (16:0)	0.018	-0.59	$3.59x10^{-2}$
TAG (49:1)_FA (17:0)	0.026	-0.59	$3.59x10^{-2}$
TAG (49:2)_FA (14:0)	0.023	-0.59	$3.59x10^{-2}$
TAG (49:2)_FA (16:1)	0.023	-0.59	$3.59x10^{-2}$
TAG (50:1)_FA (16:1)	0.031	-0.59	$3.59x10^{-2}$
TAG (50:4)_FA (18:1)	0.032	-0.59	$3.59x10^{-2}$
TAG (52:1)_FA (16:1)	0.034	-0.59	$3.59x10^{-2}$
TAG (52:5)_FA (18:1)	0.035	-0.59	$3.59x10^{-2}$
TAG (54:3)_FA (18:0)	0.047	-0.59	$3.59x10^{-2}$
TAG (54:5)_FA (20:4)	0.038	-0.59	$3.59x10^{-2}$
TAG (57:3)_FA (18:2)	0.034	-0.59	$3.59x10^{-2}$
TAG (58:7)_FA (18:1)	0.038	-0.59	$3.59x10^{-2}$
H3TG	0.047	-0.58	$3.83x10^{-2}$
IDPN	0.044	-0.57	$3.90x10^{-2}$
IDAB	0.044	-0.57	$3.90x10^{-2}$
V3PL	0.036	-0.57	$3.90x10^{-2}$
L1TG	0.044	-0.57	$3.90x10^{-2}$
DAG (18:1/20:4)	0.031	-0.57	$3.90x10^{-2}$
LPE (16:1)	0.038	-0.57	$3.90x10^{-2}$
LPE (20:3)	0.037	-0.57	$3.90x10^{-2}$

LPE (22:5)	0.043	-0.57	$3.90x10^{-2}$
MAG (16:1)	0.038	-0.57	$3.90x10^{-2}$
PE.P (18:1/22:6)	0.040	0.57	$3.90x10^{-2}$
PI (18:0/18:1)	0.031	-0.57	$3.90x10^{-2}$
PI (18:1/16:1)	0.036	-0.57	$3.90x10^{-2}$
PI (18:1/20:3)	0.037	-0.57	$3.90x10^{-2}$
TAG (46:0)_FA (14:0)	0.028	-0.57	$3.90x10^{-2}$
TAG (50:1)_FA (20:1)	0.024	-0.57	$3.90x10^{-2}$
TAG (50:2)_FA (18:2)	0.035	-0.57	$3.90x10^{-2}$
TAG (51:0)_FA (17:0)	0.032	-0.57	$3.90x10^{-2}$
TAG (53:3)_FA (17:0)	0.031	-0.57	$3.90x10^{-2}$
TAG (54:4)_FA (20:4)	0.035	-0.57	$3.90x10^{-2}$
H4CH	0.043	0.57	$4.15x10^{-2}$
TPCH	0.046	0.56	$4.17x10^{-2}$
VLPL	0.034	-0.56	$4.17x10^{-2}$
DAG (18:0/22:6)	0.028	-0.56	$4.17x10^{-2}$
DAG (18:1/22:4)	0.036	-0.56	$4.17x10^{-2}$
DAG (18:1/22:6)	0.030	-0.56	$4.17x10^{-2}$
LPC (18:3)	0.028	-0.56	$4.17x10^{-2}$
PE (18:2/18:3)	0.037	-0.56	$4.17x10^{-2}$
TAG (44:1)_FA (16:0)	0.022	-0.56	$4.17x10^{-2}$
TAG (46:0)_FA (16:0)	0.028	-0.56	$4.17x10^{-2}$
TAG (46:1)_FA (16:0)	0.022	-0.56	$4.17x10^{-2}$
TAG (49:1)_FA (14:0)	0.025	-0.56	$4.17x10^{-2}$
TAG (50:2)_FA (20:2)	0.023	-0.56	$4.17x10^{-2}$
TAG (51:2)_FA (18:2)	0.022	-0.56	$4.17x10^{-2}$
TAG (52:0)_FA (18:0)	0.042	-0.56	$4.17x10^{-2}$
TAG (52:2)_FA (18:0)	0.042	-0.56	$4.17x10^{-2}$
TAG (52:4)_FA (18:0)	0.028	-0.56	$4.17x10^{-2}$
TAG (52:4)_FA (20:4)	0.035	-0.56	$4.17x10^{-2}$
TAG (52:8)_FA (18:2)	0.032	-0.56	$4.17x10^{-2}$
TAG (54:2)_FA (18:2)	0.034	-0.56	$4.17x10^{-2}$
TAG (54:4)_FA (22:4)	0.030	-0.56	$4.17x10^{-2}$
TAG (54:6)_FA (16:0)	0.034	-0.56	$4.17x10^{-2}$
TAG (54:6)_FA (18:3)	0.027	-0.56	$4.17x10^{-2}$
TAG (55:3)_FA (18:1)	0.047	-0.56	$4.17x10^{-2}$
TAG (56:3)_FA (20:1)	0.047	-0.56	$4.17x10^{-2}$

TAG (56:5)_FA (22:4)	0.032	-0.56	$4.17x10^{-2}$
TAG (56:8)_FA (18:1)	0.029	-0.56	$4.17x10^{-2}$
3-hydroxyanthranilic acid	0.031	-0.55	$4.43x10^{-2}$
HDTG	0.049	-0.55	$4.43x10^{-2}$
IDFC	0.043	-0.55	$4.43x10^{-2}$
V1CH	0.029	-0.55	$4.43x10^{-2}$
V4CH	0.041	-0.55	$4.43x10^{-2}$
CE (18:0)	0.039	0.55	$4.43x10^{-2}$
CE (20:1)	0.035	0.55	$4.43x10^{-2}$
CE (22:5)	0.044	0.55	$4.43x10^{-2}$
DAG (16:0/16:1)	0.034	-0.55	$4.43x10^{-2}$
DAG (18:1/22:5)	0.035	-0.55	$4.43x10^{-2}$
DCER (22:1)	0.024	-0.55	$4.43x10^{-2}$
LPE (16:0)	0.035	-0.55	$4.43x10^{-2}$
PE.O (16:0/20:4)	0.034	0.55	$4.43x10^{-2}$
PE.P (18:0/22:6)	0.037	0.55	$4.43x10^{-2}$
PE.P (18:1/18:0)	0.035	-0.55	$4.43x10^{-2}$
PI (18:1/20:4)	0.030	-0.55	$4.43x10^{-2}$
SM (24:1)	0.043	0.55	$4.43x10^{-2}$
TAG (46:2)_FA (16:0)	0.021	-0.55	$4.43x10^{-2}$
TAG (46:2)_FA (18:1)	0.025	-0.55	$4.43x10^{-2}$
TAG (47:2)_FA (18:1)	0.024	-0.55	$4.43x10^{-2}$
TAG (50:4)_FA (18:3)	0.025	-0.55	$4.43x10^{-2}$
TAG (52:2)_FA (20:1)	0.023	-0.55	$4.43x10^{-2}$
TAG (52:3)_FA (18:0)	0.032	-0.55	$4.43x10^{-2}$
TAG (53:2)_FA (18:2)	0.023	-0.55	$4.43x10^{-2}$
TAG (53:3)_FA (16:0)	0.031	-0.55	$4.43x10^{-2}$
TAG (55:2)_FA (18:1)	0.048	-0.55	$4.43x10^{-2}$
TAG (55:4)_FA (18:1)	0.046	-0.55	$4.43x10^{-2}$
TAG (56:4)_FA (18:0)	0.039	-0.55	$4.43x10^{-2}$
TAG (56:6)_FA (16:0)	0.033	-0.55	$4.43x10^{-2}$
TAG (56:6)_FA (18:1)	0.041	-0.55	$4.43x10^{-2}$
L5FC	0.046	0.54	$4.56x10^{-2}$
Acetone	0.008	0.53	$4.66x10^{-2}$
Glucose	0.031	-0.54	$4.88x10^{-2}$
Xanthurenic acid	0.029	-0.54	$4.88x10^{-2}$
V3CH	0.035	-0.54	$4.88x10^{-2}$

DAG (16:0/20:3)	0.032	-0.54	4.88×10^{-2}
DCER (26:0)	0.044	0.54	4.88×10^{-2}
LPE (22:4)	0.036	-0.54	4.88×10^{-2}
PC (18:0/18:1)	0.036	-0.54	4.88×10^{-2}
PE (18:0/20:3)	0.040	-0.54	4.88×10^{-2}
PE (18:0/20:5)	0.031	-0.54	4.88×10^{-2}
TAG (45:0)_FA (16:0)	0.023	-0.54	4.88×10^{-2}
TAG (48:5)_FA (18:2)	0.031	-0.54	4.88×10^{-2}
TAG (50:2)_FA (14:0)	0.040	-0.54	4.88×10^{-2}
TAG (50:5)_FA (20:5)	0.028	-0.54	4.88×10^{-2}
TAG (52:2)_FA (16:1)	0.041	-0.54	4.80×10^{-3}
TAG (54:4)_FA (18:2)	0.036	-0.54	4.81×10^{-3}
TAG (54:7)_FA (18:3)	0.028	-0.54	4.89×10^{-2}

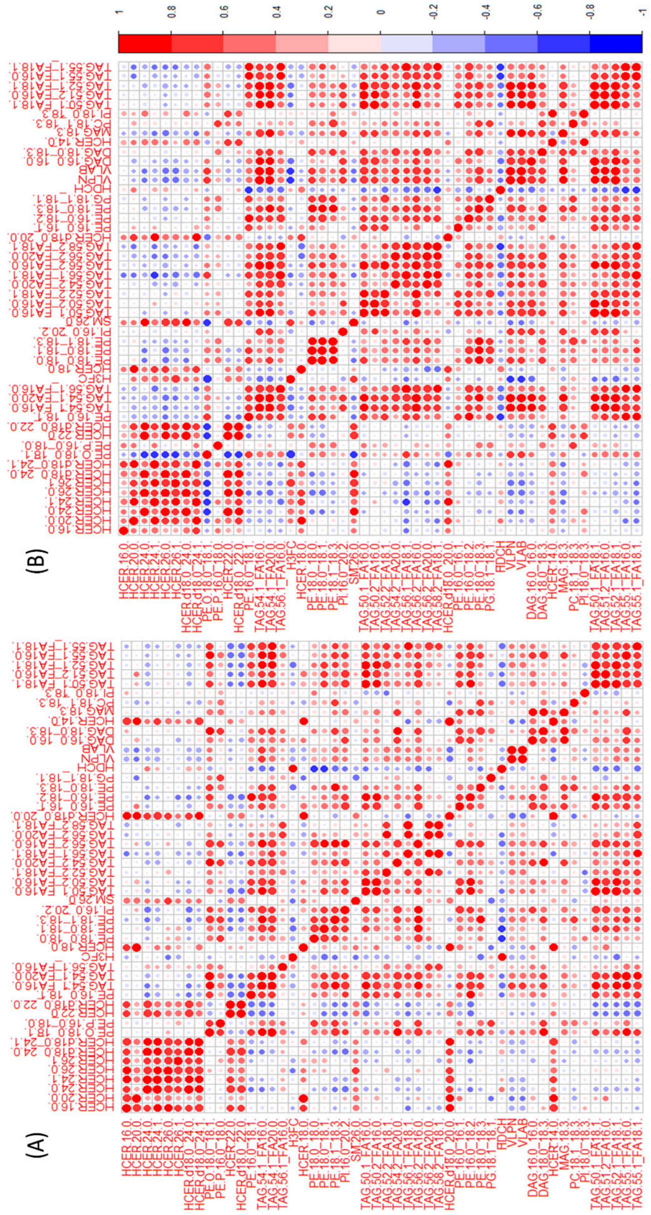

Figure S14 - Correlation plot of the (A) survived model and (B) Did not survive model. The top 50 metabolites are shown according to adjusted p-values.

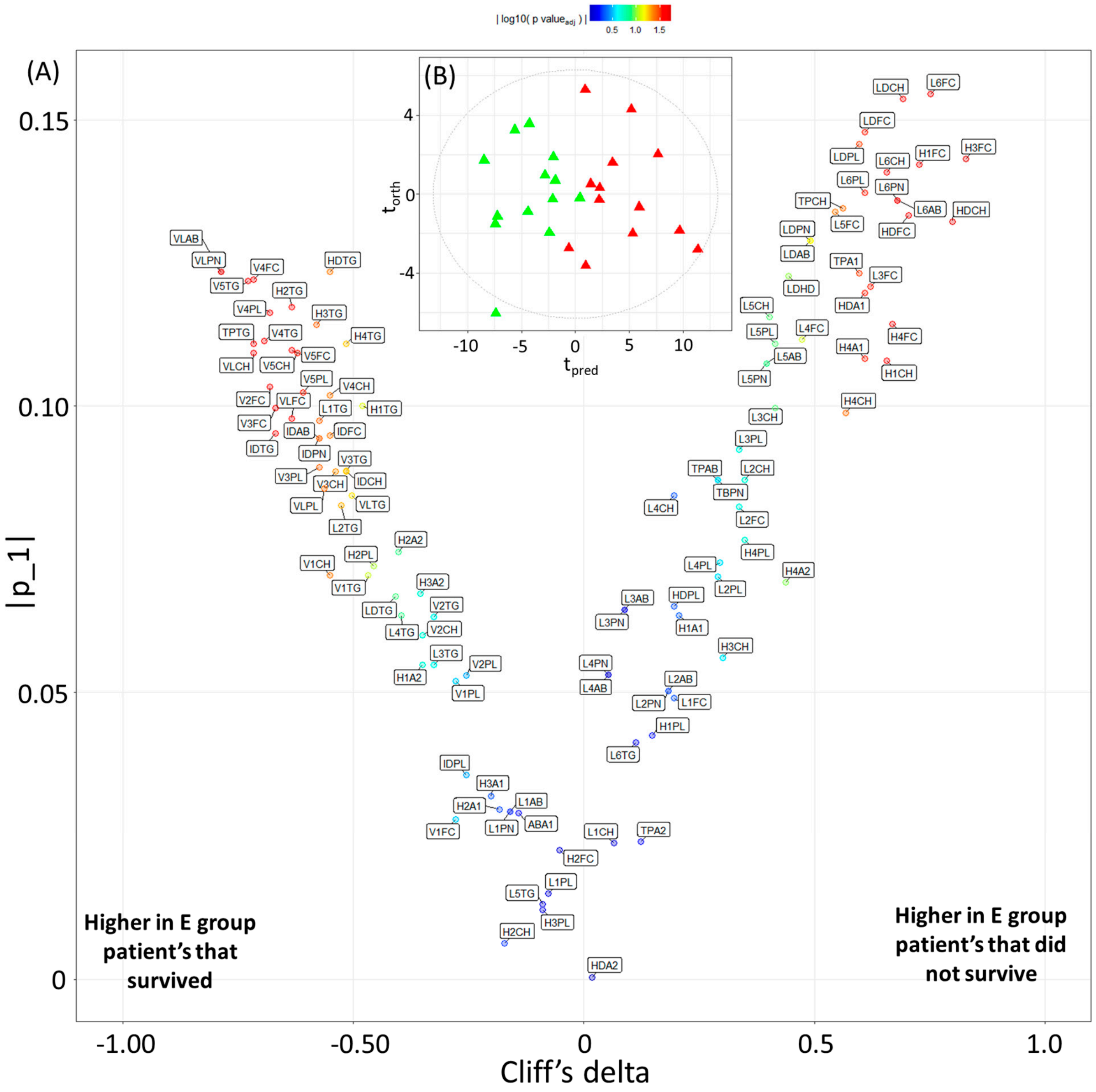

Figure S15 – Modeling COVID-19 mortality prediction using only the lipoproteins (A) Eruption plot of the severity group E patients who survived vs the severity group E patients who died. (B) O-PLS-DA of severity group E patients who survived (green triangles) and severity group E patients who died (red triangles), $R_2X=0.32$, AUROC=0.81. Cliff's delta, OPLS loadings values and the adjusted p values for this model can be found in table S30.

Table S32 - OPLS loadings, Cliff's delta and adjusted p-values of lipoprotein model for the comparison group E severity patients comparing who survived and who died, samples were age matched. Only metabolites with significant p-values are shown in the table.

Metabolite	OPLS loadings	Cliff's delta	Adjusted p-value
HDCH	0.132	0.80	$1.82x10^{-2}$
VLPN	0.123	-0.79	$1.82x10^{-2}$
VLAB	0.123	-0.79	$1.82x10^{-2}$
H3FC	0.143	0.83	$1.82x10^{-2}$
L6FC	0.154	0.75	$1.98x10^{-2}$
TPTG	0.111	-0.72	$2.04x10^{-2}$
LDCH	0.154	0.69	$2.04x10^{-2}$
L6PN	0.136	0.68	$2.04x10^{-2}$
VLCH	0.109	-0.72	$2.04x10^{-2}$
HDFC	0.133	0.70	$2.04x10^{-2}$
V4TG	0.111	-0.69	$2.04x10^{-2}$
V5TG	0.122	-0.73	$2.04x10^{-2}$
V2FC	0.103	-0.68	$2.04x10^{-2}$
V4FC	0.122	-0.72	$2.04x10^{-2}$
V4PL	0.116	-0.68	$2.04x10^{-2}$
L6AB	0.136	0.68	$2.04x10^{-2}$
H1FC	0.412	0.73	$2.04x10^{-2}$
H4FC	0.114	0.67	$2.04x10^{-2}$
IDTG	0.095	-0.67	$2.11x10^{-2}$
V3FC	0.100	-0.67	$2.11x10^{-2}$
L6CH	0.141	0.66	$2.16x10^{-2}$
H1CH	0.108	0.66	$2.25x10^{-2}$
VLFC	0.098	-0.63	$2.72x10^{-2}$
V5FC	0.110	-0.63	$2.72x10^{-2}$
H2TG	0.117	-0.63	$2.72x10^{-2}$
LDFC	0.148	0.61	$2.89x10^{-2}$
HDA1	0.120	0.61	$2.89x10^{-2}$
V5CH	0.109	-0.62	$2.89x10^{-2}$
V5PL	0.102	-0.61	$2.89x10^{-2}$
L3FC	0.121	0.62	$2.89x10^{-2}$
L6PL	0.137	0.61	$2.89x10^{-2}$
H4A1	0.108	0.61	$2.89x10^{-2}$
TPA1	0.123	0.60	$3.16x10^{-2}$

LDPL	0.146	0.60	3.16×10^{-2}
IDPN	0.094	-0.57	3.70×10^{-2}
IDAB	0.094	-0.57	3.70×10^{-2}
V3PL	0.089	-0.57	3.70×10^{-2}
L1TG	0.097	-0.57	3.70×10^{-2}
H3TG	0.114	-0.58	3.70×10^{-2}
H4CH	0.099	0.57	3.85×10^{-2}
TPCH	0.134	0.56	3.96×10^{-2}
VLPL	0.086	-0.56	3.96×10^{-2}
HDTG	0.123	-0.55	4.16×10^{-2}
IDFC	0.095	-0.55	4.16×10^{-2}
V1CH	0.071	-0.55	4.16×10^{-2}
V4CH	0.102	-0.55	4.16×10^{-2}
L5FC	0.135	0.54	4.20×10^{-2}
V3CH	0.089	-0.54	4.58×10^{-2}

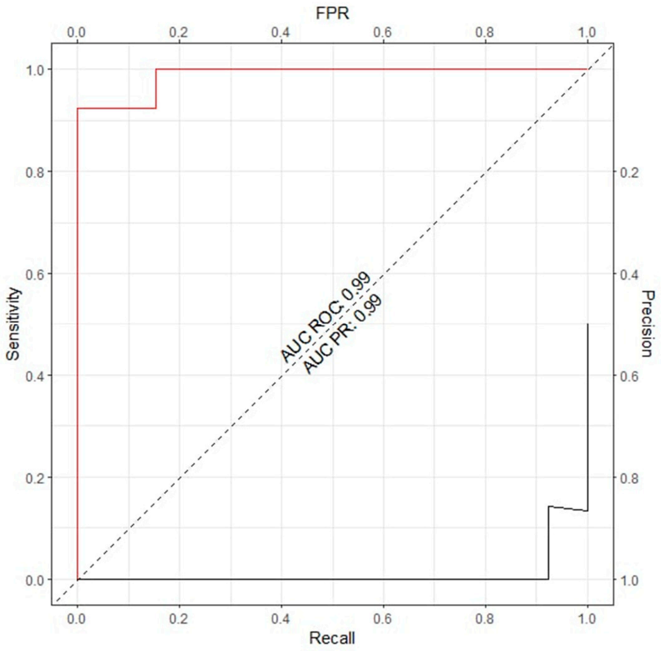

Figure S16 - AUROC showing prediction of mortality (AUCROC = 0.99) built using HCer 16:0 and PEO 18:0_18:1
